# Arf1 is involved in *Neisseria meningitidis*-induced cortical branched F-actin network reorganization

**DOI:** 10.1038/s44319-026-00843-z

**Published:** 2026-06-25

**Authors:** Margot Sahnine, Morgane Crochet, Stéphane Tachon, Sylvie Goussard, Esthel Pénard, Cyril Scandola, Magalie Duchateau, Quentin Giai Gianetto, Audrey Salles, Gaël Moneron, Léa Swistak, Arthur Charles-Orszag, Adeline Mallet, Mariette Matondo, Jean-Yves Tinevez, Daria Bonazzi, Anna Sartori-Rupp, Guillaume Duménil, Dorian Obino

**Affiliations:** 1 Institut Pasteur, Université Paris Cité, INSERM U1225, Pathogenesis of Vascular Infections Unit, F-75015 Paris, France; 2 Institut Pasteur, Université Paris Cité, NanoImaging Core Facility, F-75015 Paris, France; 3 Institut Pasteur, Université Paris Cité, Ultrastructural BioImaging Core Facility, F-75015 Paris, France; 4 Institut Pasteur, Université Paris Cité, Proteomics Core Facility, Mass Spectrometry for Biology Unit, UAR CNRS 2024, F-75015 Paris, France; 5 Institut Pasteur, Université Paris Cité, Bioinformatics and Biostatistics HUB, F-75015 Paris, France; 6 Institut Pasteur, Université Paris Cité, Photonic Bio-Imaging Unit, Centre de Ressources et Recherches Technologiques (UTechS-PBI, C2RT), F-75015 Paris, France; 7 Institut Pasteur, Université Paris Cité, CNRS UMR3571, Signaling and Receptors Dynamics Unit, F-75015 Paris, France; 8 Institut Pasteur, Université Paris Cité, CNRS UMR3691, Dynamics of Host-Pathogen Interactions Unit, F-75015 Paris, France; 9 Institut Pasteur, Université Paris Cité, Image Analysis Hub, F-75015 Paris, France; 10 Present Address: Department of Microbiology and Molecular Genetics, University of California, Davis, CA 95616 USA

**Keywords:** Cell Adhesion, Polarity & Cytoskeleton, Microbiology, Virology & Host Pathogen Interaction, Signal Transduction

## Abstract

Cells experience external forces that deform the plasma membrane to which they adapt by reorganizing their actin cytoskeleton. Here, using the extracellular bacterium *Neisseria meningitidis* as a model system, we explore how this bacterium reorganizes the cortical actin cytoskeleton subsequently to mechanical membrane deformations. Meningococci trigger the formation of tubular cellular plasma membrane protrusions by a previously described adhesion-driven process known as one-dimensional wetting. Cryo-electron tomography reveals that in epithelial cells such a deformation of the plasma membrane leads to the formation of F-actin bundles. In contrast, in endothelial cells a branched F-actin network is formed. By combining high resolution photonic microscopy approaches with genetic and drug perturbations in endothelial cells, we demonstrate that Arp2/3 activity is necessary for forming this branched network. We demonstrate the role of the nucleating-promoting factor N-WASP downstream of Cdc42. Proteomic analyses reveal the contribution of the small GTPase Arf1. Taken together, our results delineate an Arf1-Cdc42-N-WASP-Arp2/3 pathway that links mechanical plasma membrane deformation to the subsequent reorganization of a cortical branched F-actin network in endothelial cells.

## Synopsis

The small GTPase Arf1 regulates the establishment of a branched F-actin network within *N. meningitidis*-induced plasma membrane tubular protrusions in endothelial cells through the Cdc42-N-WASP-Arp2/3 molecular axis.

- A branched actin network is assembled within *N. meningitidis*-induced endothelial plasma membrane tubular protrusions.
- In epithelial cells, *N. meningitidis*-induced plasma membrane tubular protrusions encompass parallel actin filaments.
- F-actin reorganization within meningococci-induced endothelial protrusions relies on Cdc42-N-WASP-Arp2/3 activity.
- Arf1 regulates the Cdc42-N-WASP-Arp2/3 pathway, linking mechanical membrane deformation to F-actin reorganization.

## Abstract (web summary)

The small GTPase Arf1 regulates the establishment of a branched F-actin network within *N. meningitidis*-induced plasma membrane tubular protrusions in endothelial cells through the Cdc42-N-WASP-Arp2/3 molecular axis.

## Graphical abstract

**Figure:**
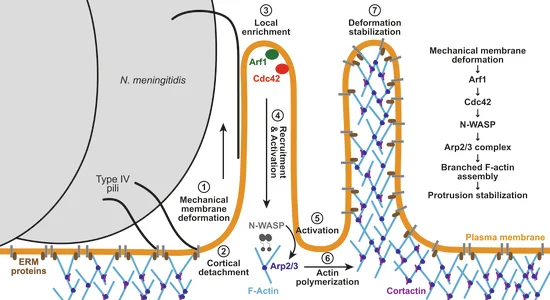

**Figure:**
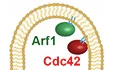

## Introduction

During interaction with their environment, the plasma membrane of eukaryotic cells is constantly submitted to external mechanical stimuli. This occurs during cell migration, adhesion, cell-to-cell interactions, and microenvironment probing (McMahon and Gallop, 2005). Adaptation to these mechanical cues is essential for the maintenance of cellular integrity and proper physiological responses. The cortical actin cytoskeleton is among the first structures to reorganize in response to these mechanical cues, thus initiating the appropriate cellular response. External mechanical perturbations and subsequent responses can also be triggered in pathological conditions, such as infections, as illustrated by the ability of certain pathogens to reshape the plasma membrane of their host cells to favor their entry, dissemination, and/or immune evasion (Ham et al, 2011; Rossman and Lamb, 2013; Sheppard and Filler, 2014). Beyond providing a better understanding of the underlying pathological processes, the use of such pathogens as tools to probe cellular functions has proven useful to decipher basic cell biological processes. This particularly applies to the extracellular bacterial pathogen *Neisseria meningitidis*, the causative agent of meningitis and/or septic shock-like systemic infections (Brandtzaeg and van Deuren, 2012; van de Beek et al, 2006). These extracellular bacteria adhere to the surface of the host cells through their type IV pili (T4P), filamentous organelles extruding from the bacterial body (Imhaus and Dumenil, 2014; Kennouche et al, 2019). T4P mechanically deform the host cell plasma membrane leading to the formation of thin filopodium-shaped protrusions that interdigitate between adhering bacteria (Dumenil, 2011; Soyer et al, 2014). This key event of meningococcal physiopathology occurs both in the nasopharynx and in blood vessels, where bacteria respectively interact with epithelial and endothelial cells (Charles-Orszag et al, 2018; Join-Lambert et al, 2013; Melican et al, 2013; Stephens, 2007; Stephens et al, 1983).

Plasma membrane protrusions are generally supported by the reorganization of the actin cytoskeleton, which provides the forces required to push the membrane forward, as observed in filopodia or lamellipodia (Le Clainche and Carlier, 2008; Ridley and Heald, 2011). Accordingly, it has been previously proposed that signaling events triggered downstream of *N. meningitidis* adhesion at the surface of endothelial cells led to F-actin reorganization, hence supporting the growth of the host cell plasma membrane protrusions (Bernard et al, 2014; Coureuil et al, 2010; Dos Santos Souza et al, 2020; Eugene et al, 2002). In contrast to this initial hypothesis, our previous results showed that *N. meningitidis*-induced plasma membrane protrusions are rather formed by external mechanical forces followed by the reorganization of the cortical actin cytoskeleton. Several arguments are in favor of this sequence of events. First, actin reorganization only occurs seconds after plasma membrane deformation (Soyer et al, 2014). Second, chemical inhibition of intracellular ATP or actin polymerization does not prevent plasma membrane remodeling (Charles-Orszag et al, 2018; Soyer et al, 2014). Accordingly, we have recently shown that *N. meningitidis* triggers the formation of these membrane protrusions through a physical process referred to as one-dimensional wetting (1DW). 1DW is a general adhesion-driven process that promotes the formation and elongation of membrane tubular structures along adhesive nano-scale fibers (≤ 6 nm in diameter) (Charles-Orszag et al, 2018). In the context of meningococcal infections, this process is initiated at the scale of a single bacterium through the interaction of meningococcal type IV pili with the host cell plasma membrane, and is subsequently amplified as bacteria proliferate and auto-aggregate (Charles-Orszag et al, 2018; Kennouche et al, 2019). Accordingly, blocking the interaction of pili with the host cell surface with antibodies prevents plasma membrane remodeling and the subsequent reorganization of the actin cytoskeleton (Charles-Orszag et al, 2018). Interestingly, the closely related pathogen *Neisseria gonorrhoeae* was also shown to induce cortical actin reorganization by exerting mechanical forces on host cells through pilus retraction (Merz et al, 1999). Consequently, this called to revisit the early sequence of events induced upon *N. meningitidis* adhesion. In particular, how the extrinsic remodeling of the plasma membrane by 1DW leads to cortical F-actin reorganization remains to be elucidated.

It should be emphasized that the massive remodeling of the host cell plasma membrane induced by *N. meningitidis* has an important impact on meningococcal intravascular pathogenesis. First, membrane remodeling allows bacteria to resist high levels of shear stress while proliferating at the surface of the infected endothelial cells (Mikaty et al, 2009). Importantly, it is the membrane remodeling itself rather than the associated F-actin reorganization that is key to resistance to shear stress, as actin polymerization inhibitors such as Latrunculin A have no effect on bacteria-induced plasma membrane reorganization and bacterial resistance to flow (Mairey et al, 2006; Mikaty et al, 2009). In addition, adhesion of meningococci at the surface of endothelial cells has been shown to be associated with the local relocalization of endothelial junctional proteins, thus weakening cell-cell junctions and facilitating bacterial dissemination into the host (Coureuil et al, 2009). Deciphering the intricate interplay between bacteria, the deformation of the host cell plasma membrane, and the subsequent actin reorganization is not only important to better understand how bacteria manipulate host cells in pathological contexts, but beyond infection, it also allows us to explore how cells reorganize in response to plasma membrane deformation induced by external mechanical cues.

In this study, we used *N. meningitidis* as a model to trigger the formation of plasma membrane protrusions by 1DW in both human primary nasopharyngeal epithelial cells and venular endothelial cells. By combining high-resolution light and electron microscopy approaches, we shed light on the ultrastructural organization of F-actin networks within these tubular plasma membrane protrusions formed following *N. meningitidis* adhesion at the surface of both epithelial and endothelial cells. Strikingly, our data show that tubular membrane protrusions induced by meningococci in epithelial cells lead to the assembly of linear F-actin filaments, whereas in endothelial cells this results in the polymerization of a branched F-actin network, thus highlighting distinct mechanisms in both cell types. By coupling chemical and genetic perturbation experiments to proteomic analysis, we deciphered the molecular axis regulating the subcellular activity of the Arp2/3 complex in endothelial tubular protrusions and shed light on the key role of the small GTPases Cdc42 and Arf1. This study, therefore, paves the way to our understanding of how cells react to local external mechanical cues in complex environments under various pathophysiological conditions.

## Results

### *Neisseria meningitidis*-induced membrane deformation in epithelial and endothelial cells results in protrusions of distinct morphologies

**Figure EV1:**
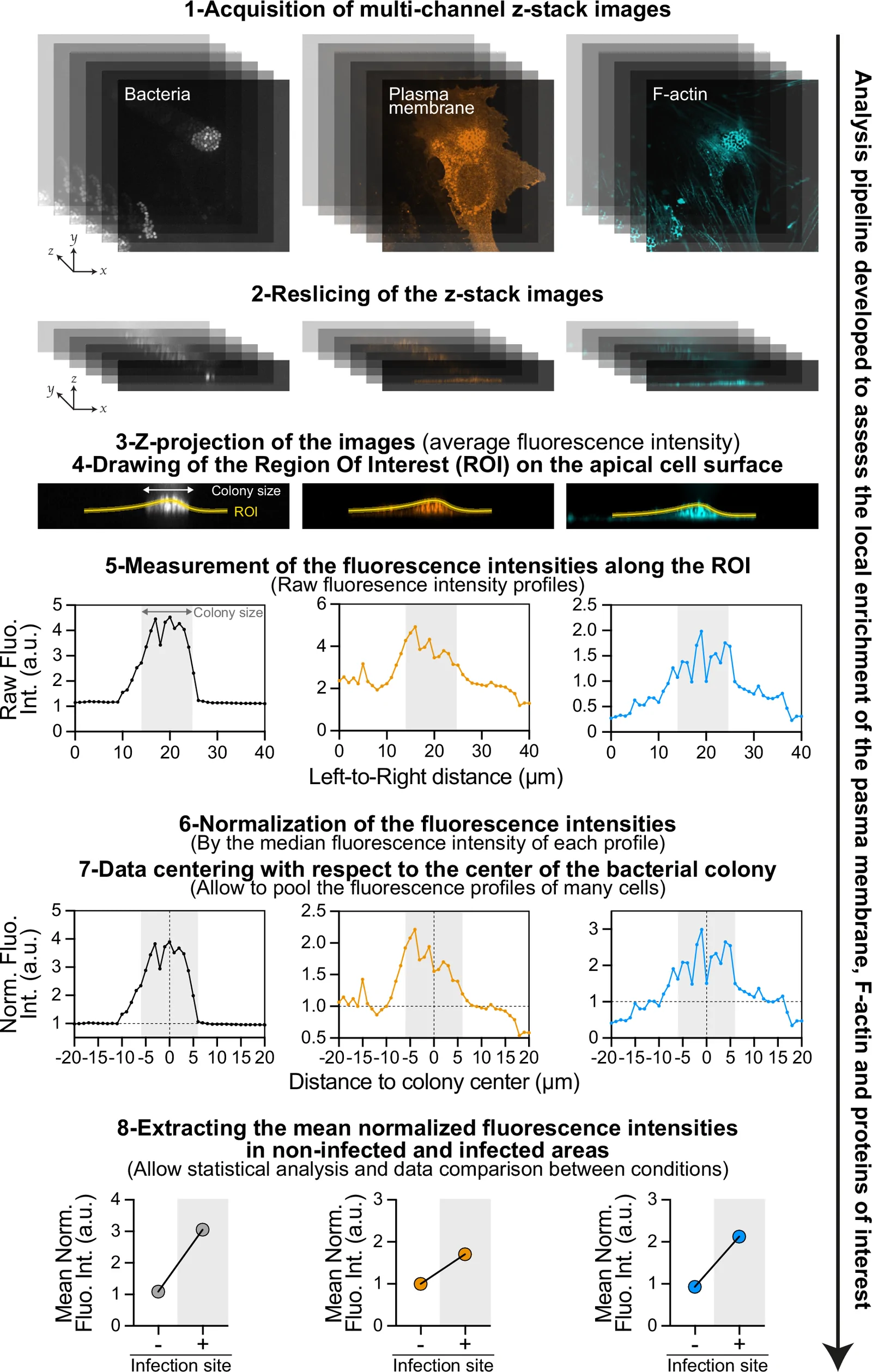
Analysis pipeline used to quantify the enrichment of markers of interest underneath *N. meningitidis* aggregates. To accurately quantify the local enrichment of the different markers of interest underneath bacterial aggregates on the apical surface of infected cells we developed a sequential approach. The multi-channel z-stack images (1) were resliced (2) and the focal planes containing bacteria-derived fluorescent signal were averaged projected (3). Following the manual drawing of the region of interest (ROI) along the apical surface of the cells (4), measurements of the raw fluorescence intensities in the different fluorescent channels were performed using Fiji (5). Data were then normalized with respect to the median value of the fluorescence intensity profile on the per cell basis (6) and centered according to the geometric center of the bacterial aggregate (7), hence allowing to average the profiles of various cells. Finally, the mean value of the normalized fluorescence intensities per channel and per cell in the non-infected and infected areas were extracted and plotted as paired observations (8).

**Figure 1:**
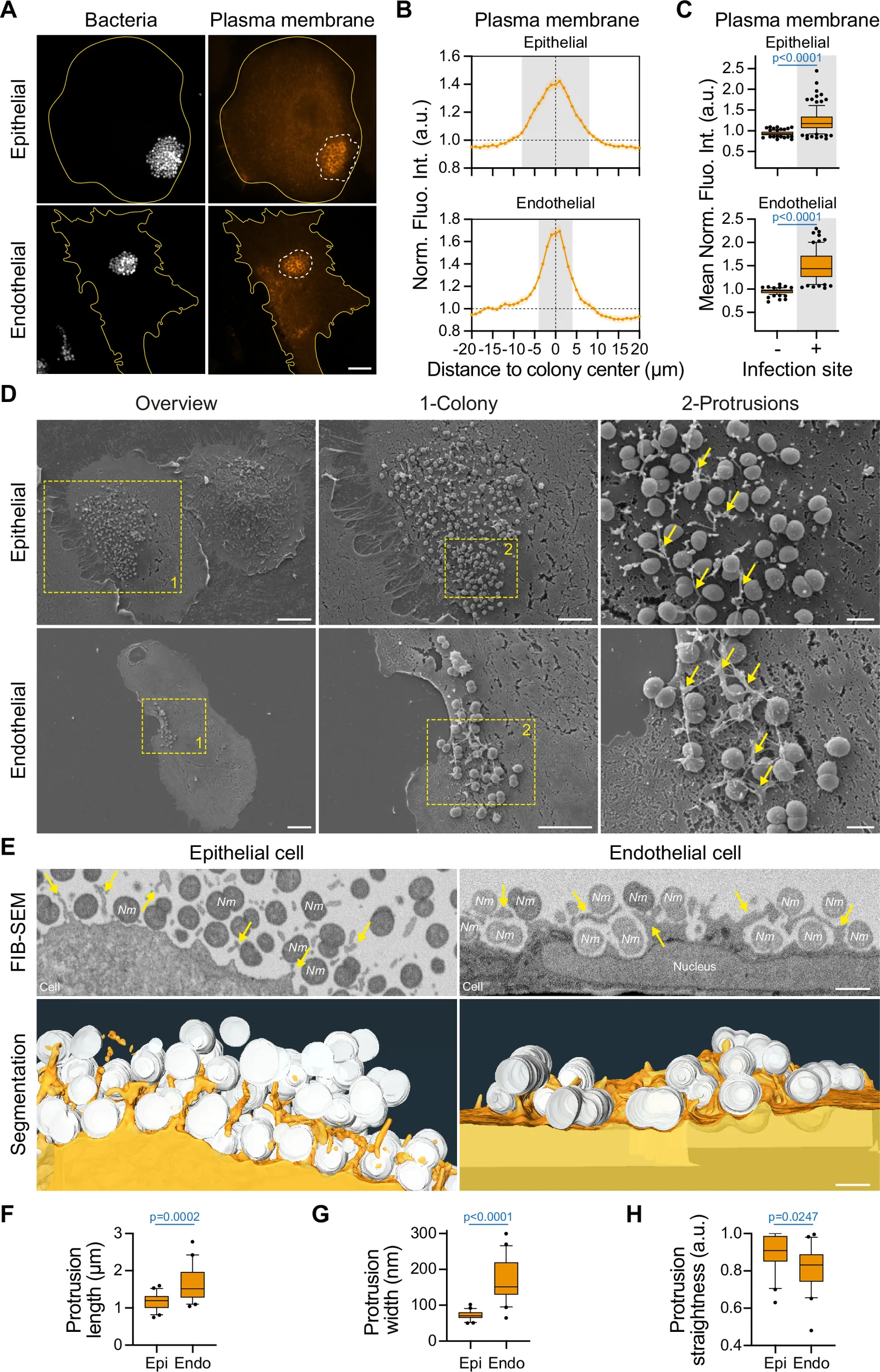
Morphological analysis of membrane protrusions formed following plasma membrane deformation by *N. meningitidis* in human epithelial and endothelial cells. (**A**) Representative images (maximum intensity projections) of cells infected with iRFP-expressing *N. meningitidis* (gray). The plasma membrane (orange) of epithelial cells was stained using anti-CD326/EpCAM antibody and that of endothelial cells visualized thanks to the transient expression of a plasmid encoding the plasma membrane probe PM-EGFP. The yellow lines delineate the cell contour. The white dashed lines delineate the contour of bacterial aggregates. Scale bar: 10 µm. (**B**) Normalized fluorescence intensity profiles of the plasma membrane in epithelial (top) and endothelial (bottom) cells infected with iRFP-expressing *N. meningitidis*. The light gray areas show the average colony size. Data are shown as mean ± SEM. (**C**) Mean value per cell of the normalized fluorescence intensity of the plasma membrane in the non-infected (−) and infected (+) areas in epithelial (top) and endothelial (bottom) cells infected with iRFP-expressing *N. meningitidis*. The Wilcoxon matched-pairs signed-rank test was used to assess statistical significance. (**A**–**C**) *n* = 106 epithelial cells and 80 endothelial cells, pooled from *N* = 3 biological replicates. (**D**) Representative images of epithelial (top) and endothelial (bottom) cells infected with *N. meningitidis* and imaged by scanning electron microscopy (SEM). The yellow dashed squares highlight the zoomed areas successively shown on the right. The yellow arrows show epithelial- and endothelial-derived membrane protrusions induced upon *N. meningitidis* adhesion. Scale bars: 10, 5, and 1 µm, from left to right, respectively. Representative of *n* = 5 *N. meningitidis*-infected cells per cell type, from *N* = 1 biological replicate. (**E**) Top: Representative FIB-SEM images (one slice extracted from the entire volume) of *N. meningitidis*-infected epithelial (left) and endothelial (right) cells. The yellow arrows show epithelial- and endothelial-derived membrane protrusions induced upon *N. meningitidis* infection. *Bottom:* Individual bacteria (white) and the host cell plasma membrane (orange) were segmented on the FIB-SEM volumes, and 3D reconstructed. Scale bars: 1 µm. (**F**–**H**) Quantification of the length (**F**), width (**G**), and straightness (**H**) of membrane protrusions formed by epithelial and endothelial cells upon *N. meningitidis* infection. An unpaired *t*-test was used to assess statistical significance. *n* = 20 protrusions per cell type, analyzed from *N* = 1 FIB-SEM volume per cell type. (**C**,** F**–**H**) Boxes in box plots extend from the 25th to the 75th percentile, with a line at the median, whiskers extend from the 10th to the 90th percentile, and dots show outlier values. *P* values are shown on the graphs. Source data are available online for this figure.

The morphology of membrane protrusions relies on the intrinsic properties of each cell type to deform their plasma membrane. While epithelial cells are known to form microvilli at their surface, endothelial cells rather assemble lamellipodium-like structures known as dactylopodia (Figueiredo et al, 2021; Sauvanet et al, 2015). We therefore first sought to characterize the morphology of *N. meningitidis*-induced plasma membrane protrusions in both human primary epithelial and endothelial cells. To accurately quantify plasma membrane remodeling underneath bacterial microcolonies, i.e., on the apical side of the cells, we developed an image analysis pipeline used throughout the study based on the reorientation of z-stack images along the xz-axis (Fig. EV1). As expected, this analysis confirmed that adhesion of meningococci at the surface of both cell types led to the local enrichment of plasma membrane underneath bacterial aggregates, although to a lesser extent in infected epithelial cells compared to infected endothelial cells (Fig. 1A–C).

To assess the morphology of these membrane protrusions in both epithelial and endothelial cells, we next used electron microscopy-based approaches to obtain ultrastructural information with a nanometer-scale resolution. Scanning electron microscopy (SEM) imaging highlighted that the tubular protrusions formed by endothelial cells were longer and thicker compared to the short protrusions found in epithelial cells (Fig. 1D, yellow arrows). Furthermore, while meningococci were intertwined within a network of protrusions at the surface of endothelial cells, epithelial cell-derived protrusions appeared sparser (Fig. 1D). These observations were confirmed by focused ion beam (FIB)-SEM imaging, allowing the acquisition of large three-dimensional volumes (Fig. 1E; Movies EV1 and EV2). Volume segmentation was then performed to delineate the host cell plasma membrane and individual meningococci and visualize the protrusions (Fig. 1E, yellow arrows; Movies EV1 and EV2). Measurements of protrusion length, width, and straightness underneath entire bacterial microcolonies confirmed that endothelial cells formed longer and thicker sinuous membrane protrusions than epithelial cells (Fig. 1F–H).

These data indicate that the morphology of *N. meningitidis*-induced tubular membrane protrusions is different according to the host cell type, pointing to different underlying mechanisms.

### Cryo-electron tomography reveals distinct F-actin networks in epithelial and endothelial protrusions following bacteria-induced membrane deformation

**Figure 2:**
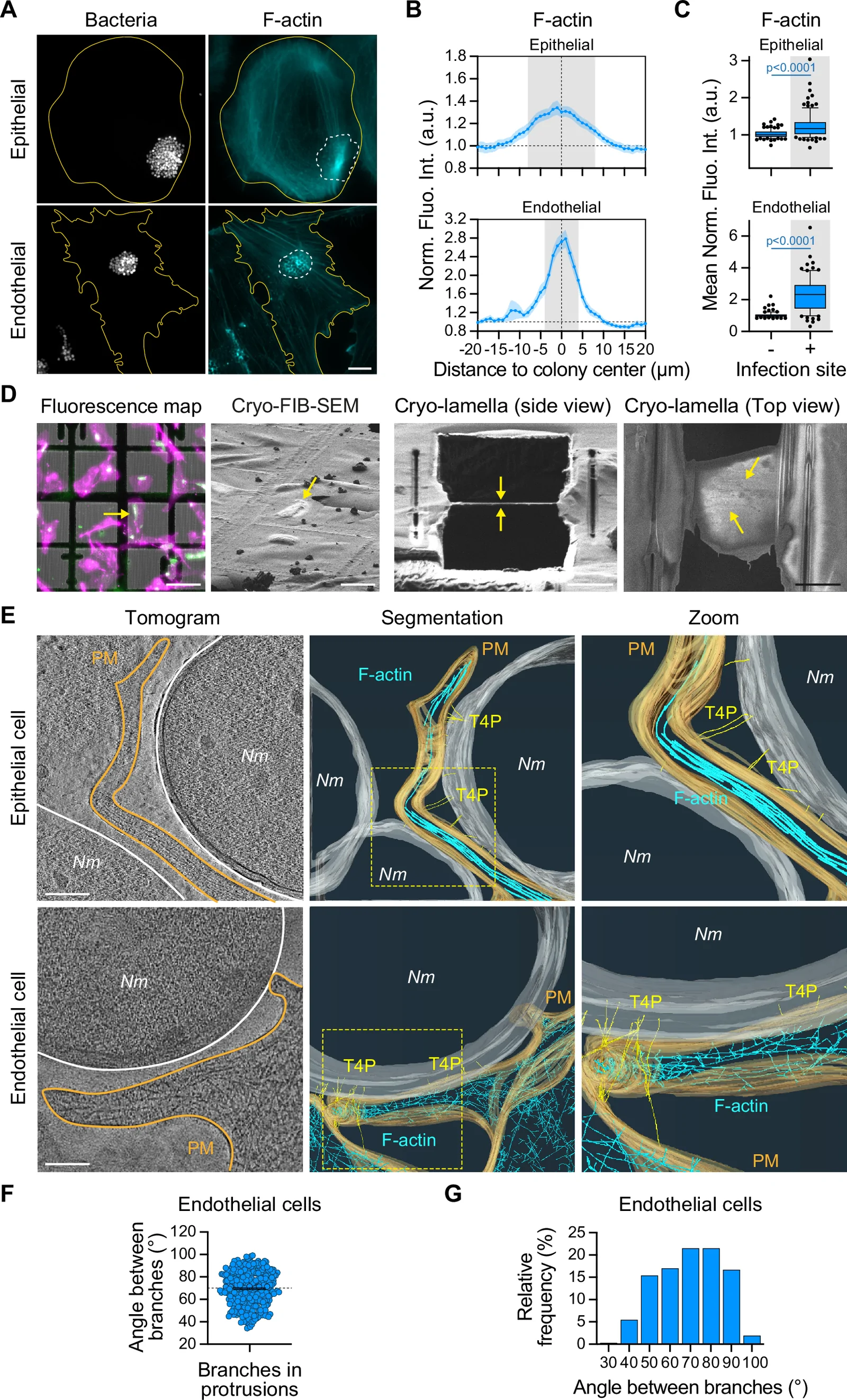
Structural analysis of F-actin within *N. meningitidis*-induced protrusions in epithelial and endothelial cells. (**A**) Representative images (maximum intensity projections) of epithelial (top) and endothelial (bottom) cells infected with iRFP-expressing *N. meningitidis* (gray) and stained for F-actin (phalloidin, cyan). The yellow lines delineate the cell contour. The white dashed lines delineate the contour of bacterial aggregates. Scale bar: 10 µm. Note that pictures of bacteria shown here are the same as in Fig. 1A. (**B**) Normalized fluorescence intensity profiles of F-actin in epithelial (top) and endothelial (bottom) cells infected with iRFP-expressing *N. meningitidis*. The light gray areas show the average colony size. Data were shown as mean ± SEM. (**C**) Mean value per cell of the normalized fluorescence intensity of F-actin in the non-infected (−) and infected (+) areas in epithelial (top) and endothelial (bottom) cells infected with iRFP-expressing *N. meningitidis*. Boxes in box plots extend from the 25th to the 75th percentile, with a line at the median, whiskers extend from the 10th to the 90th percentile, and dots show outlier values. The Wilcoxon matched-pairs signed-rank test was used to assess statistical significance. *P* values are shown on the graphs. (**A**–**C**) *n* = 106 epithelial cells and 80 endothelial cells, pooled from *N* = 3 biological replicates. (**D**) Left: Representative area of endothelial cells seeded on carbon-coated grid with alphanumeric coordinates, infected with GFP-expressing *N. meningitidis* (green) and stained for the plasma membrane (UEA-1 lectin, magenta). Scale bar: 30 µm. The same area imaged at the cryo-FIB-SEM microscope is shown in the middle image. Scale bar: 10 µm. The yellow arrows show the *N. meningitidis*-infected endothelial cell used to generate the cryo-lamella. Right: Side and top views of the corresponding cryo-lamella. The yellow arrows show bacteria. Scale bars: 5 µm. (**E**) Left: Representative TEM images of the cryo-lamellae generated in *N. meningitidis*-infected epithelial (top) and endothelial (bottom) cells. One slice through a tomogram is shown. Scale bars: 100 nm. Middle and Right: Manual segmentation and 3D reconstruction of the different structures observed in *N. meningitidis*-infected epithelial (top) and endothelial (bottom) cells are shown. The yellow dashed squares highlight the zoomed areas shown on the right. The cell plasma membrane is shown in orange, F-actin in cyan, bacteria in white, and T4P in yellow. Representative of *n* = 4 tomograms per cell type, from *N* = 1 and 3 biological replicates for epithelial and endothelial cells, respectively. (**F**,** G**) Measurement (**F**) and frequency distribution (**G**) of angles between F-actin branches in tubular membrane protrusions in *N. meningitidis*-infected endothelial cells. *n* = 311 angles, pooled from *N* = 4 tomograms. Source data are available online for this figure.

We next sought to determine whether the cell type-specific protrusion morphologies we observed also translated into distinct F-actin organizations in epithelial and endothelial tubular membrane protrusions. As expected, immunofluorescence analysis of infected epithelial and endothelial cells showed a local enrichment of F-actin underneath bacterial microcolonies in both cell types, although less important in epithelial cells (Fig. 2A–C).

F-actin networks assemble according to characteristic patterns, either in Formin-dependent linear filaments or in branched networks that are polymerized by the branched actin-nucleating complex Arp2/3 (Chesarone and Goode, 2009; Le Clainche and Carlier, 2008). Therefore, resolving F-actin ultrastructural organizations within these tubular protrusions will provide insightful information on the underlying molecular mechanisms. To do so, we setup a correlative strategy by combining the mapping of infected cells on carbon-coated grids with alphanumeric coordinates by fluorescence microscopy to high-resolution imaging of F-actin ultrastructure within protrusions using electron tomography on cryo-lamellae (Berger et al, 2023) (Fig. 2D). This approach not only allowed to visualize F-actin networks within *N. meningitidis*-induced tubular protrusions, but also to resolve meningococcal T4P connecting the membrane protrusions to the bacterial body with nanometer-scale resolution in infected cells preserved in a quasi-native state (Fig. 2E). Three-dimensional segmentation and annotation of the tomograms revealed that epithelial cell-derived membrane protrusions contained parallel F-actin filaments (Fig. 2E; Movie EV3), suggesting the involvement of proteins of the Formin family, similarly to what has been described in epithelial microvilli or filopodia (Chesarone et al, 2010). Strikingly and in sharp contrast, endothelial protrusions enclosed a branched F-actin network (Fig. 2E; Movie EV4), strongly suggesting a role for the Arp2/3 complex in these cells. Accordingly, measuring the angles between F-actin branches on the segmented volumes revealed a characteristic mean angle value of 70° ± 1° and ranging from 40° to 100° (Fig. 2F,G), as previously described for Arp2/3-mediated F-actin branches in in vitro reconstituted systems (Machesky and Way, 1998; Mullins et al, 1998).

Taken together, these observations argue for the existence of cell type-specific mechanisms supporting F-actin reorganization in *N. meningitidis*-induced tubular plasma membrane protrusions in epithelial and endothelial cells, respectively. Furthermore, while most filopodium-like protrusions encompass linear actin filaments (Le Clainche and Carlier, 2008), the unexpected observation of a branched F-actin network in endothelial protrusions called for further investigations to decipher the molecular mechanisms regulating the nucleation of branched F-actin filaments in these cells.

### F-actin reorganization in *N. meningitidis*-induced endothelial tubular protrusions relies on Arp2/3 activity

**Figure 3:**
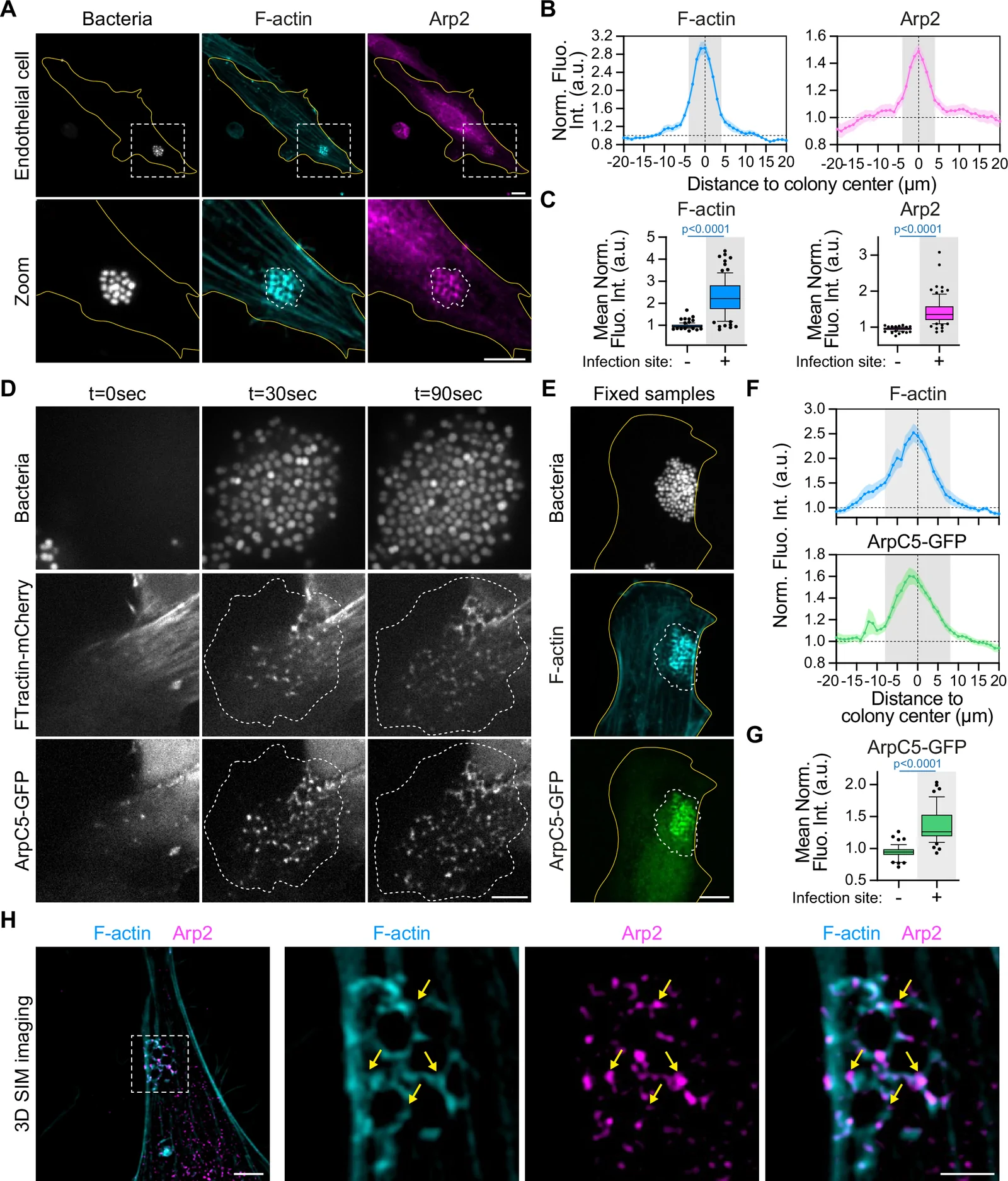
The Arp2/3 complex is locally enriched in endothelial protrusions following *N. meningitidis*-induced plasma membrane deformation. (**A**) Representative images (maximum intensity projections) of endothelial cells infected with BFP-expressing *N. meningitidis* (gray) and stained for F-actin (phalloidin, cyan) and endogenous Arp2 (specific antibody, magenta). The yellow lines delineate the cell contour. The white dashed squares highlight the zoomed area shown on the bottom row. The white dashed lines on the zoomed pictures delineate the contour of bacterial aggregates. Scale bars: 10 µm. (**B**) Normalized fluorescence intensity profiles of F-actin and Arp2 in endothelial cells infected with BFP-expressing *N. meningitidis*. The light gray areas show the average colony size. Data were shown as mean ± SEM. (**C**) Mean value per cell of the normalized fluorescence intensity of F-actin and Arp2 in the non-infected (−) and infected (+) areas in endothelial cells infected with BFP-expressing *N. meningitidis*. The Wilcoxon matched-pairs signed-rank test was used to assess statistical significance. (**A**–**C**) *n* = 86 infected endothelial cells, pooled from *N* = 3 biological replicates. (**D**) Representative snapshot images extracted from the time-lapse acquisition (1 image/6 s) of hTERT-immortalized endothelial cells stably expressing FTractin-mCherry and transiently transfected with ArpC5-GFP prior to being infected with preformed iRFP-expressing *N. meningitidis* aggregates. The white dashed lines delineate the contour of bacterial aggregates. Scale bar: 5 µm. Representative of *n* = 15 infected cells, imaged from *N* = 3 biological replicates. (**E**) Representative images (maximum intensity projections) of endothelial cells transiently expressing ArpC5-GFP, infected with iRFP-expressing *N. meningitidis* (gray) and stained for F-actin (phalloidin, cyan). The yellow lines delineate the cell contour. The white dashed lines delineate the contour of bacterial aggregates. Scale bar: 10 µm. (**F**) Normalized fluorescence intensity profiles of F-actin and (ArpC5-)GFP in ArpC5-GFP-expressing endothelial cells infected with iRFP-expressing *N. meningitidis*. The light gray areas show the average colony size. Data were shown as mean ± SEM. (**G**) Mean value per cell of the normalized fluorescence intensity of (ArpC5-)GFP in the non-infected (−) and infected (+) areas in ArpC5-GFP-expressing endothelial cells infected with iRFP-expressing *N. meningitidis*. The Wilcoxon matched-pairs signed-rank test was used to assess statistical significance. (**E**–**G**) *n* = 48 infected endothelial cells, pooled from *N* = 3 biological replicates. (**C**,** G**) Boxes in box plots extend from the 25th to the 75th percentile, with a line at the median, whiskers extend from the 10th to the 90th percentile, and dots show outlier values. *P* values are shown on the graphs. (**H**) Representative structured illumination microscopy (SIM) images of endothelial cells infected with BFP-expressing *N. meningitidis* (not shown), fixed, and stained for F-actin (phalloidin, cyan) and Arp2 (specific antibody, magenta). The white dashed square shows the zoomed area corresponding to the site of *N. meningitidis* infection. The yellow arrows show Arp2 puncta within membrane protrusions formed by 1D wetting. Scale bars: 2 µm. Representative of *n* = 15 infected cells, acquired over *N* = 3 biological replicates. Source data are available online for this figure.

**Figure EV2:**
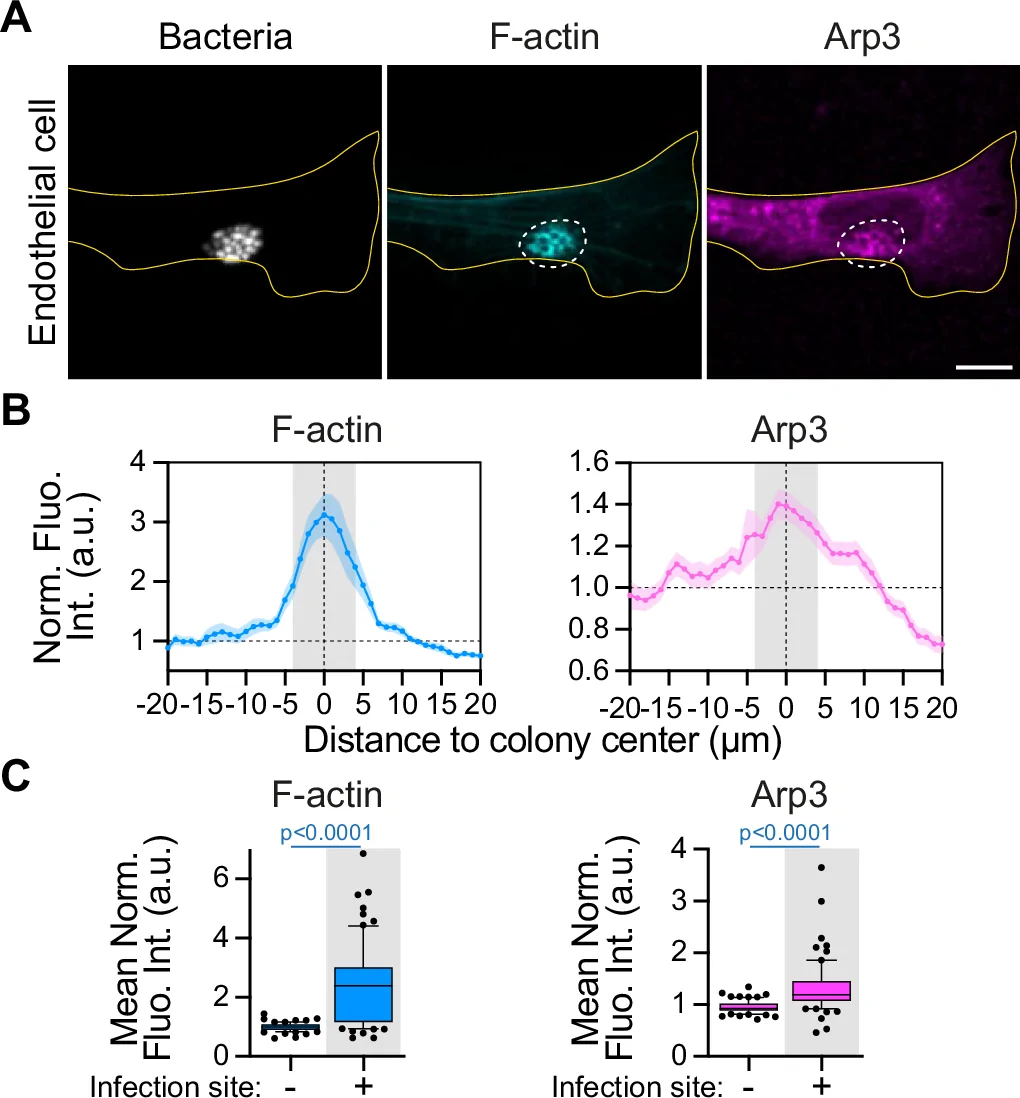
Local enrichment of Arp2/3 in endothelial protrusions formed following *N. meningitidis*-induced plasma membrane deformation. (**A**) Representative images (maximum intensity projections) of endothelial cells infected with BFP-expressing *N. meningitidis* (gray) and stained for F-actin (phalloidin, cyan) and endogenous Arp3 (specific antibody, magenta). The yellow lines delineate the cell contour. The white dashed lines delineate the contour of the bacterial aggregates. Scale bars: 10 µm. (**B**) Normalized fluorescence intensity profiles of F-actin and Arp3 in endothelial cells infected with BFP-expressing *N. meningitidis*. The light gray areas show the average colony size. Data are shown as mean ± SEM. (**C**) Mean value per cell of the normalized fluorescence intensity of F-actin and Arp3 in the non-infected (−) and infected (+) areas in endothelial cells infected with BFP-expressing *N. meningitidis*. Boxes in box plots extend from the 25th to the 75th percentile, with a line at the median, whiskers extend from the 10th to the 90th percentile, and dots show outlier values. The Wilcoxon matched-pairs signed-rank test was used to assess statistical significance. *P* values are shown on the graphs. (**A**–**C**) *n* = 70 infected endothelial cells, pooled from *N* = 3 biological replicates.

We next sought to assess the local recruitment of Arp2/3 within endothelial protrusions formed following *N. meningitidis*-induced membrane deformation. Using our quantitative image analysis pipeline (Fig. EV1) on fixed samples, we found that the endogenous core subunit Arp2 was locally enriched within endothelial tubular protrusions (Fig. 3A–C). Equivalent results were obtained when assessing the local enrichment of endogenous Arp3 (Fig. EV2A–C) or that of the accessory subunit ArpC5-GFP transiently expressed in endothelial cells by live and fixed imaging experiments (Fig. 3D–G; Movie EV5), as previously reported for this subunit (Coureuil et al, 2009). These results confirm the specific enrichment of the Arp2/3 complex in *N. meningitidis*-induced tubular membrane protrusions in endothelial cells.

We next used 3D structured illumination microscopy (SIM) to gain resolution and information on the structural organization and confirmed the presence of both Arp2 and F-actin within endothelial cell-derived membrane protrusions upon meningococcal infection (Fig. 3H). Interestingly, Arp2 fluorescence signal was organized in discrete puncta that decorated actin filaments within the protrusions, occupying spaces devoid of actin signal or at branching points (Fig. 3H, yellow arrows).

**Figure EV3:**
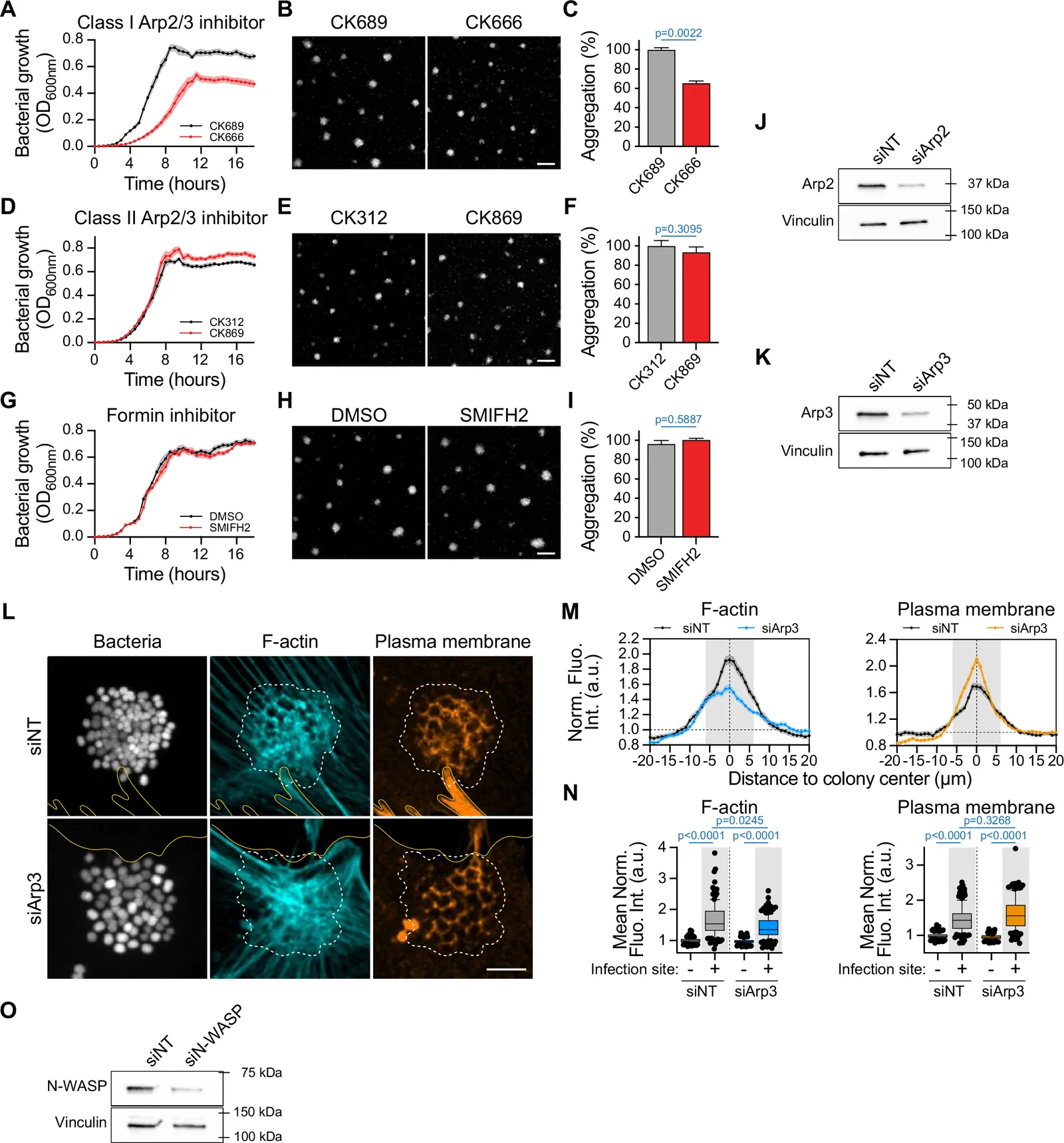
Silencing Arp2/3 expression impairs F-actin reorganization in endothelial tubular protrusions formed following *N. meningitidis*-induced plasma membrane deformation. (**A**,** D**,** G**) Bacterial growth curves of *N. meningitidis* liquid cultures in the presence of (**A**) the Arp2/3 class I inhibitor CK666 or its corresponding inactive control compound CK689 (100 µM final); (**D**) the Arp2/3 class II inhibitor CK869 or its corresponding inactive control compound CK312 (20 µM final); or (**G**) the Formin inhibitor SMIFH2 and its vehicle control DMSO (1 µM final). The optical density at 600 nm of each culture was measured every 30 min for 18 h, each in technical triplicate. Data were pooled from *N* = 3 biological replicates and are displayed as the mean ± SEM. (**B**,** E**,** H**) Representative images of bacterial aggregates formed by GFP-expressing *N. meningitidis* following a 30 min incubation in the presence of either (**B**) CK689 or CK666 (100 µM final); (**E**) CK312 and CK869 (20 µM final); or (**H**) DMSO and SMIFH2 (1 µM final). One representative, out of *N* = 2 biological replicates, is shown. (**C**,** F**,** I**) The surface covered by bacterial aggregates was quantified in each field of view (three images per condition) using an in-house developed Fiji macro and normalized with respect to the mean value of the control condition (CK689, CK312, or DMSO, respectively). The Mann–Whitney test was used to assess statistical significance. Bar graphs show the mean±sem of *n* = 6 measurements per condition, pooled from *N* = 2 biological replicates. *P* values are shown on the graphs. (**J**,** K**) Western blot analysis of the efficiency of (**J**) Arp2 and (**K**) Arp3 silencing. Vinculin was used as a loading control. The immunoblots shown are representative of *N* = 4 biological replicates. (**L**) Representative images (maximum intensity projections) of control (siNT) and Arp3 (siArp3)-silenced endothelial cells infected with iRFP-expressing *N. meningitidis* (gray) and stained for F-actin (phalloidin, cyan) and the plasma membrane (CD31, orange). The yellow lines delineate the cell contour. The white dashed lines delineate the contour of bacterial aggregates. Note that pictures of control (siNT) infected endothelial cells shown here are the same as in Fig. 4G. Scale bar: 5 µm. (**M**) Normalized fluorescence intensity profiles of F-actin and the plasma membrane in control (siNT) and Arp3 (siArp3)-silenced endothelial cells infected with iRFP-expressing *N. meningitidis*. The light gray areas show the average colony size. Data are shown as mean ± SEM. (**N**) Mean value per cell of the normalized fluorescence intensity of F-actin and the plasma membrane in the non-infected (−) and infected (+) areas in control (siNT) and Arp3 (siArp3)-silenced endothelial cells infected with iRFP-expressing *N. meningitidis*. Boxes in box plots extend from the 25th to the 75th percentile, with a line at the median, whiskers extend from the 10th to the 90th percentile, and dots show outlier values. Kruskal–Wallis test was used to assess statistical significance. *P* values are shown on the graphs. (**L**–**N**) *n* = 146 and 138 infected endothelial cells for siNT and siArp3, respectively, pooled from *N* = 4 biological replicates. (**O**) Western blot analysis of the efficiency of N-WASP silencing. Vinculin was used as a loading control. The immunoblot shown is representative of *N* = 2 biological replicates.

**Figure 4:**
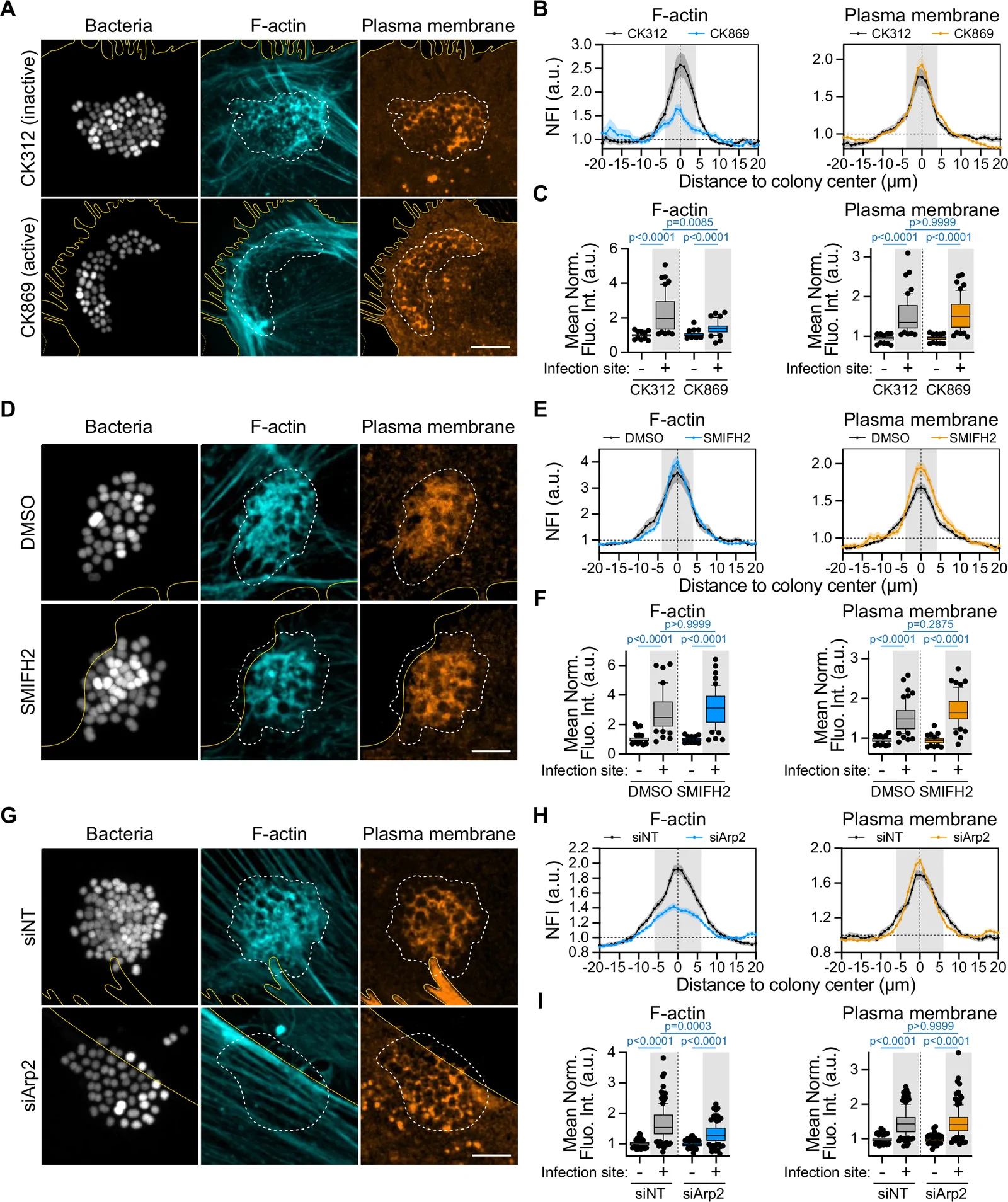
Arp2/3, but not Formin, inhibition impairs F-actin reorganization in endothelial protrusions formed following *N. meningitidis*-induced plasma membrane deformation. (**A**) Representative images (maximum intensity projections) of endothelial cells transiently expressing the plasma membrane reporter PM-GFP (orange), infected with iRFP-expressing *N. meningitidis* (gray), treated prior and during infection with either CK312 (control, inactive compound) or CK869 (Arp2/3 inhibitor, active compound) and stained for F-actin (phalloidin, cyan). The yellow lines delineate the cell contour. The white dashed lines delineate the contour of bacterial aggregates. Scale bar: 5 µm. (**B**) Normalized fluorescence intensity profiles of F-actin and the plasma membrane in PM-GFP-expressing endothelial cells treated with either CK312 or CK869 and infected with iRFP-expressing *N. meningitidis*. The light gray areas show the average colony size. Data were shown as mean ± SEM. (**C**) Mean value per cell of the normalized fluorescence intensity of F-actin and the plasma membrane in the non-infected (−) and infected (+) areas in PM-GFP-expressing endothelial cells treated with either CK312 or CK869 and infected with iRFP-expressing *N. meningitidis*. Kruskal–Wallis test was used to assess statistical significance. (**A**–**C**) *n* = 55 and 42 infected endothelial cells for CK312 and CK869, respectively, pooled from *N* = 3 biological replicates. (**D**) Representative images (maximum intensity projections) of endothelial cells transiently expressing the plasma membrane reporter PM-GFP (orange), infected with iRFP-expressing *N. meningitidis* (gray), treated prior and during infection with either control (DMSO) or SMIFH2 and stained for F-actin (phalloidin, cyan). The yellow lines delineate the cell contour. The white dashed lines delineate the contour of bacterial aggregates. Scale bar: 5 µm. (**E**) Normalized fluorescence intensity profiles of F-actin and the plasma membrane in PM-GFP-expressing endothelial cells treated with either DMSO or SMIFH2 and infected with iRFP-expressing *N. meningitidis*. The light gray areas show the average colony size. Data were shown as mean ± SEM. (**F**) Mean value per cell of the normalized fluorescence intensity of F-actin and the plasma membrane in the non-infected (−) and infected (+) areas in PM-GFP-expressing endothelial cells treated with either DMSO or SMIFH2 and infected with iRFP-expressing *N. meningitidis*. The Kruskal–Wallis test was used to assess statistical significance. (**D**–**F**) *n* = 64 and 58 infected endothelial cells for DMSO and SMIFH2, respectively, pooled from *N* = 3 biological replicates. (**G**) Representative images (maximum intensity projections) of control (siNT) and Arp2 (siArp2)-silenced endothelial cells infected with iRFP-expressing *N. meningitidis* (gray) and stained for F-actin (phalloidin, cyan) and the plasma membrane (CD31, orange). The yellow lines delineate the cell contour. The white dashed lines delineate the contour of bacterial aggregates. Scale bar: 5 µm. (**H**) Normalized fluorescence intensity profiles of F-actin and the plasma membrane in control (siNT) and Arp2 (siArp2)-silenced endothelial cells infected with iRFP-expressing *N. meningitidis*. The light gray areas show the average colony size. Data were shown as mean ± SEM. (**I**) Mean value per cell of the normalized fluorescence intensity of F-actin and the plasma membrane in the non-infected (−) and infected (+) areas in control (siNT) and Arp2 (siArp2)-silenced endothelial cells infected with iRFP-expressing *N. meningitidis*. Kruskal–Wallis test was used to assess statistical significance. (**G**–**I**) *n* = 146 and 134 infected endothelial cells for siNT and siArp2, respectively, pooled from *N* = 4 biological replicates. (**C**,** F**,** I**) Boxes in box plots extend from the 25th to the 75th percentile, with a line at the median, whiskers extend from the 10th to the 90th percentile, and dots show outlier values. *P* values are shown on the graphs. Source data are available online for this figure.

To confirm the involvement of Arp2/3 in regulating F-actin reorganization in endothelial tubular protrusions, we next attempted to chemically inhibit Arp2/3 activity. However, we could not use the well-described class I Arp2/3 inhibitor CK666 (Nolen et al, 2009) in our conditions since it reduced the ability of bacteria to proliferate and auto-aggregate (Fig. EV3A–C). Alternatively, we used the class II Arp2/3 inhibitor CK869, and its non-active control compound CK312 (Nolen et al, 2009), which showed minimal impact on bacteria (Fig. EV3D–F). Treatment of endothelial cells with CK869 prior to and during membrane remodeling led to a drastic local disorganization of F-actin when compared to CK312-treated cells (Fig. 4A). This was confirmed by quantifying F-actin fluorescence intensity profile on the apical side of infected endothelial cells (Fig. 4B,C). Conversely, treating endothelial cells with the Formin inhibitor SMIFH2 (Rizvi et al, 2009) did not impact the local reorganization of the actin cytoskeleton, nor affected bacterial growth or auto-aggregation (Figs. 4D–F and EV3G–I). Importantly, membrane remodeling was not affected by the treatments, as revealed by the local enrichment of the plasma membrane in all conditions (Fig. 4A–F) and in agreement with our previous findings (Soyer et al, 2014).

To circumvent any potential deleterious effect of the drugs, we next treated immortalized endothelial cells (Hayer et al, 2016) with pooled siRNA directed against Arp2 or Arp3, using non-targeting siRNA (siNT) as a control. As expected, decreasing the expression of Arp2 or Arp3 at the protein level phenocopied Arp2/3 inhibition by CK869 and led to a severe impairment of F-actin enrichment within *N. meningitidis*-induced tubular protrusions in infected endothelial cells (Figs. 4G–I and EV3J–N). As previously, Arp2/3 silencing did not affect the formation of membrane protrusions (Figs. 4G–I and EV3L–N).

Together, these data confirm the instrumental role played by the Arp2/3 complex in locally regulating F-actin reorganization following *N. meningitidis*-mediated plasma membrane deformation in endothelial cells.

### Cdc42 regulates Arp2/3 activity through N-WASP in endothelial protrusions induced by *N. meningitidis*

**Figure 5:**
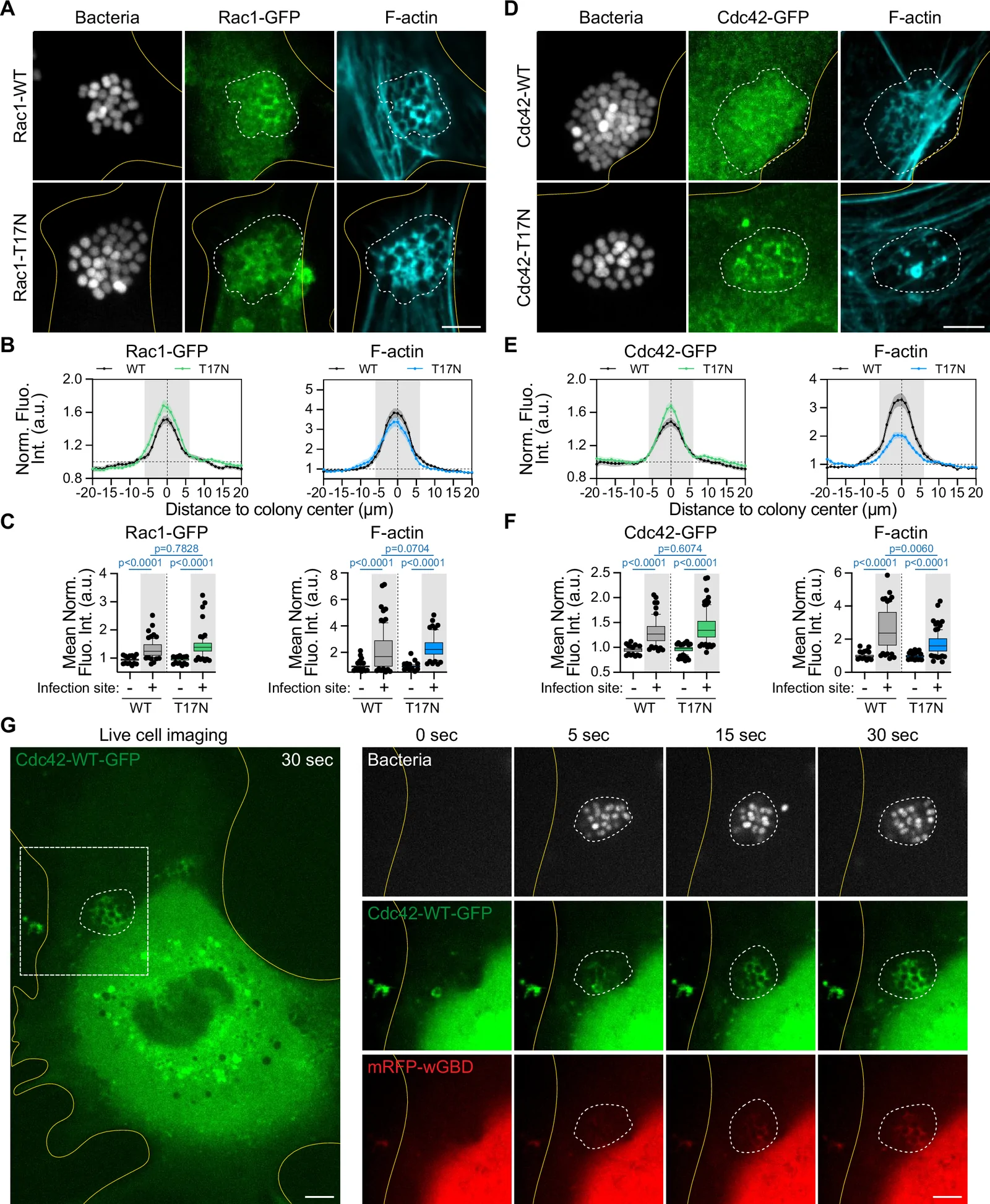
Cdc42, but not Rac1, is required for the proper reorganization of F-actin within endothelial tubular protrusions formed following *N. meningitidis*-induced plasma membrane deformation. (**A**) Representative images (maximum intensity projections) of endothelial cells transiently expressing either the wild-type (WT) or a dominant negative mutant (T17N) version of Rac1 fused to GFP (green), infected with iRFP-expressing *N. meningitidis* (gray), and stained for F-actin (phalloidin, cyan). The yellow lines delineate the cell contour. The white dashed lines delineate the contour of bacterial aggregates. Scale bar: 5 µm. (**B**) Normalized fluorescence intensity profiles of (Rac1-)GFP and F-actin in Rac1-WT and T17N-expressing endothelial cells infected with iRFP-expressing *N. meningitidis*. The light gray areas show the average colony size. Data were shown as mean ± SEM. (**C**) Mean value per cell of the normalized fluorescence intensity of (Rac1-)GFP and F-actin in the non-infected (−) and infected (+) areas in Rac1-WT and T17N-expressing endothelial cells infected with iRFP-expressing *N. meningitidis*. The Kruskal–Wallis test was used to assess statistical significance. (**A**–**C**) *n* = 79 and 74 infected endothelial cells for Rac1-WT and T17N, respectively, pooled from *N* = 3 biological replicates. (**D**) Representative images (maximum intensity projections) of endothelial cells transiently expressing either the wild-type (WT) or a dominant negative mutant (T17N) version of Cdc42 fused to GFP (green), infected with iRFP-expressing *N. meningitidis* (gray), and stained for F-actin (phalloidin, cyan). The yellow lines delineate the cell contour. The white dashed lines delineate the contour of bacterial aggregates. Scale bar: 5 µm. (**E**) Normalized fluorescence intensity profiles of (Cdc42-)GFP and F-actin in Cdc42-WT and T17N-expressing endothelial cells infected with iRFP-expressing *N. meningitidis*. The light gray areas show the average colony size. Data were shown as mean ± SEM. (**F**) Mean value per cell of the normalized fluorescence intensity of (Cdc42-)GFP and F-actin in the non-infected (−) and infected (+) areas in Cdc42-WT and T17N-expressing endothelial cells infected with iRFP-expressing *N. meningitidis*. The Kruskal–Wallis test was used to assess statistical significance. (**D**–**F**) *n* = 75 and 93 infected endothelial cells for Cdc42-WT and T17N, respectively, pooled from *N* = 3 biological replicates. (**C**,** F**) Boxes in box plots extend from the 25th to the 75th percentile, with a line at the median, whiskers extend from the 10th to the 90th percentile, and dots show outlier values. *P* values are shown on the graphs. (**G**) Representative snapshot images (average intensity projections) of endothelial cells transiently co-expressing Cdc42-WT-GFP (green) and the GTP-bound Cdc42 reporter mRFP-wGBD (red) and infected with preformed iRFP-expressing *N. meningitidis* aggregates (gray). The yellow lines delineate the cell contour. The white dashed lines delineate the contour of the bacterial aggregate. The white dashed square in the left picture denotes the zoomed area shown as a time series on the right. Scale bars: 5 µm. *n* = 5 infected cells, from *N* = 1 biological replicate. Source data are available online for this figure.

We next sought to decipher the molecular pathway regulating Arp2/3 activity in endothelial tubular protrusions upon *N. meningitidis*-induced mechanical membrane deformation. Strikingly, while previous reports have suggested the involvement of the small GTPase Cdc42, but not that of Rac1, in regulating F-actin reorganization in endothelial cells upon meningococcal infection (Coureuil et al, 2009; Eugene et al, 2002), others have rather shown a role for Rac1 (Lambotin et al, 2005). We therefore reassessed this question and evaluated which of these small GTPases locally regulated Arp2/3 activity in endothelial tubular protrusions under our conditions. To do so, endothelial cells were transiently transfected with either the wild-type (WT) or a dominant negative mutant (T17N) version of Rac1 or Cdc42 fused to GFP (Kraynov et al, 2000; Nalbant et al, 2004) and infected with meningococci to trigger the remodeling of the host cell plasma membrane. Strikingly, while both versions of Rac1 and Cdc42 were locally enriched within endothelial tubular protrusions, only the expression of the dominant negative mutant version of Cdc42 (Cdc42-T17N) strongly impaired the local reorganization of F-actin (Fig. 5A–F). Accordingly, live-cell imaging of endothelial cells transiently co-expressing both Cdc42-WT-GFP and the GTP-bound Cdc42 reporter mRFP-wGBD (Benink and Bement, 2005) showed the local enrichment of activated Cdc42 within the protrusions (Fig. 5G; Movie EV6).

**Figure 6:**
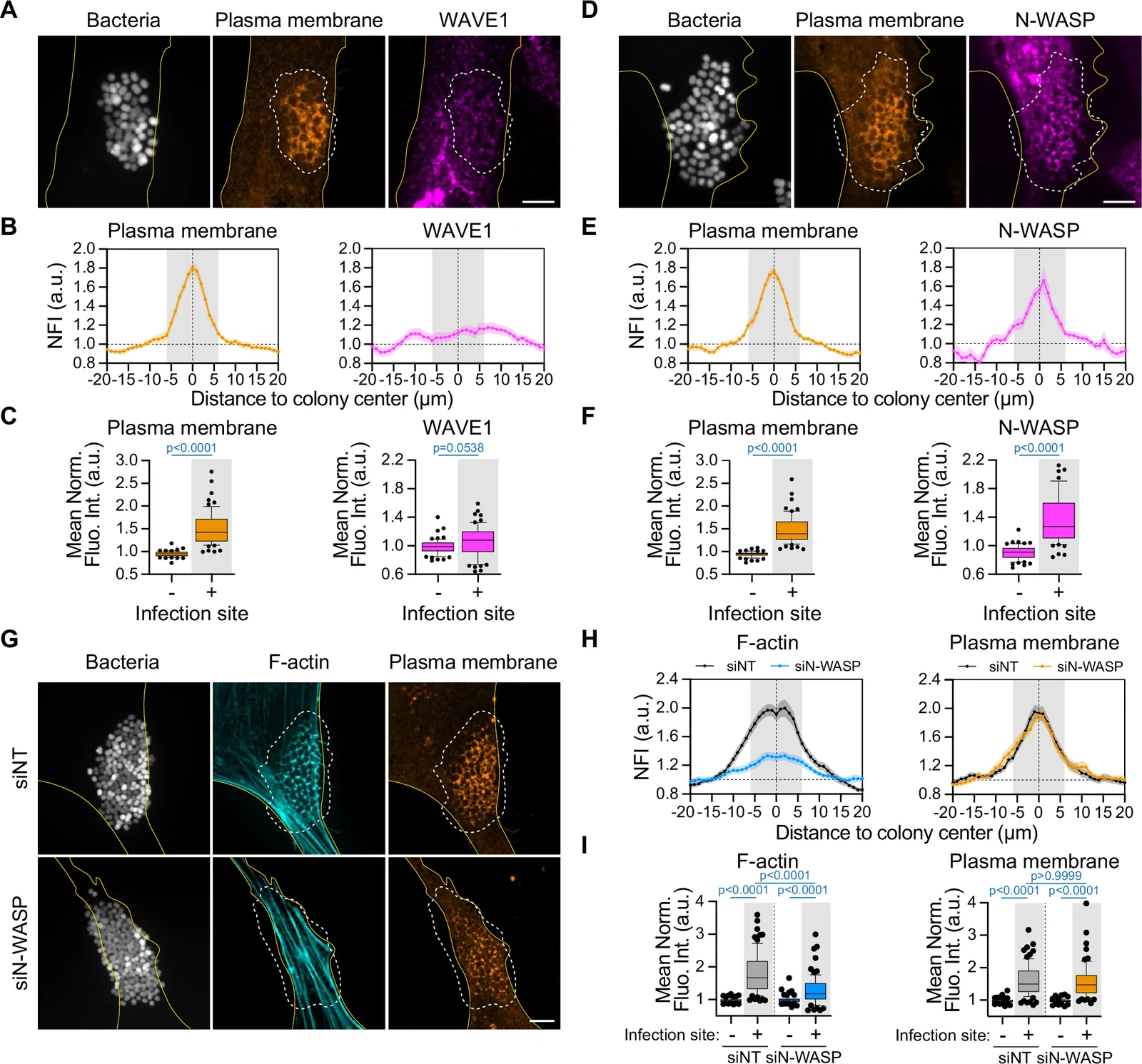
N-WASP, but not WAVE1, is required for the proper reorganization of F-actin within endothelial tubular protrusions formed following *N. meningitidis*-induced plasma membrane deformation. (**A**) Representative images (maximum intensity projections) of endothelial cells transiently expressing the plasma membrane probe PM-GFP (orange), infected with iRFP-expressing *N. meningitidis* (gray) and stained for endogenous WAVE1 (specific antibody, magenta). The yellow lines delineate the cell contour. The white dashed lines delineate the contour of the bacterial aggregates. Scale bar: 5 µm. (**B**) Normalized fluorescence intensity profiles of the plasma membrane and WAVE1 in PM-GFP-expressing endothelial cells infected with iRFP-expressing *N. meningitidis*. The light gray areas show the average colony size. Data were shown as mean ± SEM. (**C**) Mean value per cell of the normalized fluorescence intensity of the plasma membrane and WAVE1 in the non-infected (−) and infected (+) areas in PM-GFP-expressing endothelial cells infected with iRFP-expressing *N. meningitidis*. A paired *t*-test was used to assess statistical significance. (**A**–**C**) *n* = 65 infected endothelial cells, pooled from *N* = 3 biological replicates. (**D**) Representative images (maximum intensity projections) of endothelial cells transiently expressing the plasma membrane probe PM-GFP (orange), infected with iRFP-expressing *N. meningitidis* (gray) and stained for endogenous N-WASP (specific antibody, magenta). The yellow lines delineate the cell contour. The white dashed lines delineate the contour of the bacterial aggregates. Scale bar: 5 µm. (**E**) Normalized fluorescence intensity profiles of the plasma membrane and N-WASP in PM-GFP-expressing endothelial cells infected with iRFP-expressing *N. meningitidis*. The light gray areas show the average colony size. Data were shown as mean ± SEM. (**F**) Mean value per cell of the normalized fluorescence intensity of the plasma membrane and N-WASP in the non-infected (−) and infected (+) areas in PM-GFP-expressing endothelial cells infected with iRFP-expressing *N. meningitidis*. A paired *t*-test was used to assess statistical significance. (**D**–**F**) *n* = 63 infected endothelial cells, pooled from *N* = 3 biological replicates. (**G**) Representative images (maximum intensity projections) of control (siNT) and N-WASP (siN-WASP)-silenced endothelial cells infected with iRFP-expressing *N. meningitidis* (gray) and stained for F-actin (phalloidin, cyan) and the plasma membrane (CD31, orange). The yellow lines delineate the cell contour. The white dashed lines delineate the contour of bacterial aggregates. Scale bar: 5 µm. (**H**) Normalized fluorescence intensity profiles of F-actin and the plasma membrane in control (siNT) and N-WASP (siN-WASP)-silenced endothelial cells infected with iRFP-expressing *N. meningitidis*. The light gray areas show the average colony size. Data were shown as mean ± SEM. (**I**) Mean value per cell of the normalized fluorescence intensity of F-actin and the plasma membrane in the non-infected (−) and infected (+) areas in control (siNT) and N-WASP (siN-WASP)-silenced endothelial cells infected with iRFP-expressing *N. meningitidis*. The Kruskal–Wallis test was used to assess statistical significance. (**G**–**I**) *n* = 77 and 78 infected cells for siNT and siN-WASP, respectively, pooled from *N* = 2 biological replicates. (**C**,** F**,** I**) Boxes in box plots extend from the 25th to the 75th percentile, with a line at the median, whiskers extend from the 10th to the 90th percentile, and dots show outlier values. *P* values are shown on the graphs. Source data are available online for this figure.

In search for the molecular pathway linking Cdc42 to Arp2/3 activity, we next explored the involvement of the nucleating-promoting factors (NPFs) WAVE1 and N-WASP, since both NPFs have been previously shown to regulate the nucleation of Arp2/3-dependent branched F-actin networks in other cellular processes (Campellone and Welch, 2010). Immunofluorescence analysis and quantifications showed the absence of enrichment of endogenous WAVE1 in endothelial tubular protrusions triggered by *N. meningitidis* adhesion in our conditions (Fig. 6A–C). Rather, our analysis revealed that endogenous N-WASP was locally enriched in these protrusions (Fig. 6D–F). Accordingly, silencing N-WASP expression using pooled siRNA impaired F-actin reorganization within endothelial tubular protrusions, while not affecting plasma membrane remodeling (Figs. 6G–I and EV3O).

Taken together, these results show that Arp2/3 activity is locally regulated by the Cdc42-N-WASP molecular axis within membrane protrusions formed following the mechanical deformation of the plasma membrane by *N. meningitidis* in endothelial cells.

### Meningococcal infection modifies Arp2/3 and Cdc42 interactomes in endothelial cells

**Figure 7:**
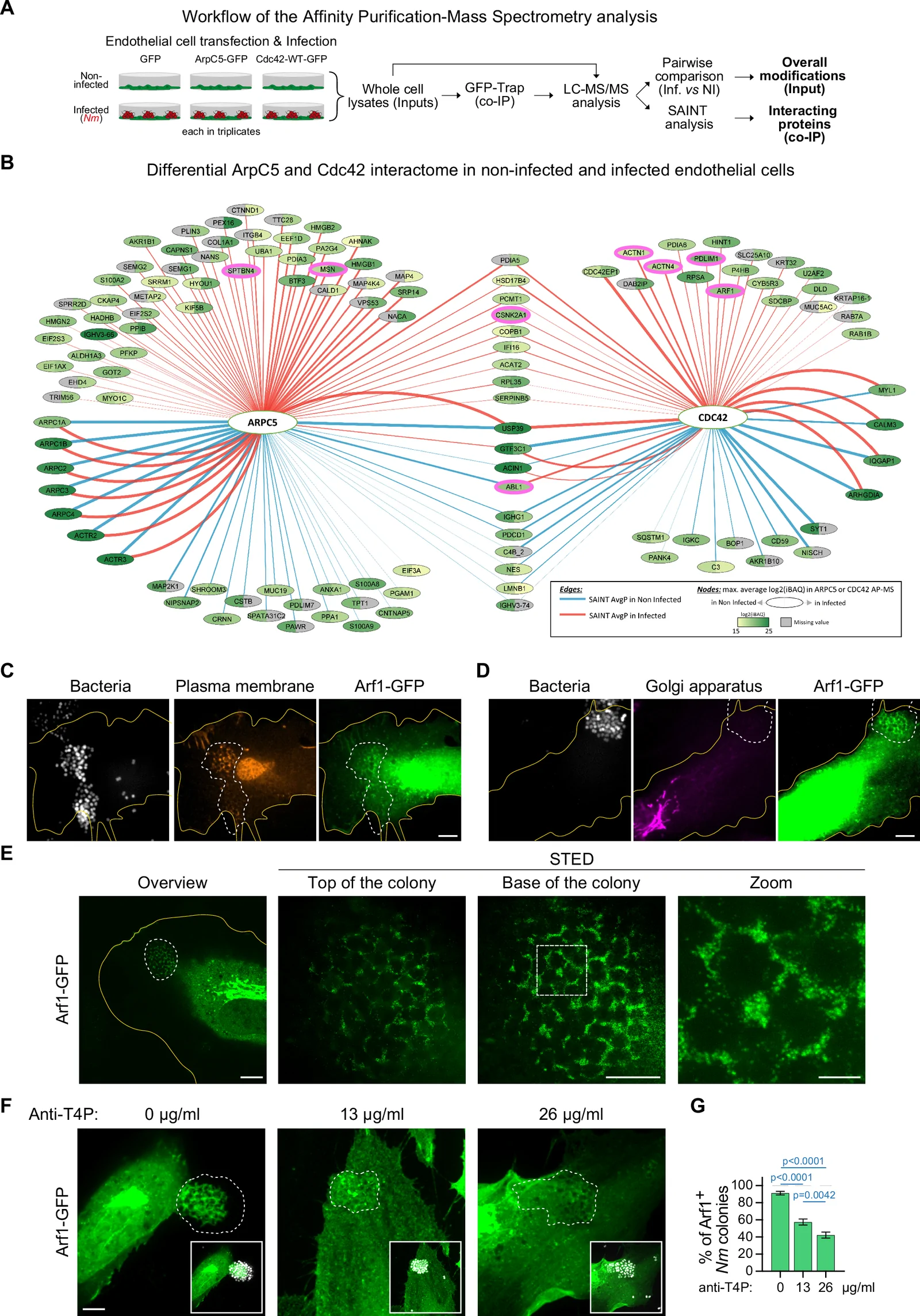
Infection of endothelial cells modifies Arp2/3 and Cdc42 interactomes. (**A**) Schematic representation of the GFP-Trap approach used to identify ArpC5-GFP and Cdc42-WT-GFP-interacting proteins by GFP-based co-immunoprecipitation and mass spectrometry (AP-MS) analysis. (**B**) Network representation of protein–protein interactions identified in non-infected (blue lines) and infected (red lines) endothelial cells using ArpC5-GFP or Cdc42-WT-GFP as bait proteins (highlighted in the center). Results were obtained from three technical replicates per condition. (**C**) Representative images (average intensity projections) of endothelial cells transiently expressing Arf1-GFP (green), infected with iRFP-expressing *N. meningitidis* (gray) and stained for the plasma membrane (CD31, orange). The GFP signal was saturated at the Golgi apparatus to visualize the small fraction of Arf1-GFP at the plasma membrane. The yellow lines delineate the cell contour. The white dashed lines delineate the contour of bacterial aggregates. Scale bar: 5 µm. Representative of *n* = 50 infected cells, pooled from *N* = 2 biological replicates. (**D**) Representative images (average intensity projections) of endothelial cells transiently expressing Arf1-GFP (green), infected with iRFP-expressing *N. meningitidis* (gray) and stained for the Golgi apparatus (GM130, magenta). The GFP signal was saturated at the Golgi apparatus to visualize the small fraction of Arf1-GFP at the plasma membrane. The yellow lines delineate the cell contour. The white dashed lines delineate the contour of bacterial aggregates. Scale bar: 5 µm. Representative of *n* = 63 infected cells, pooled from *N* = 2 biological replicates. (**E**) Representative Stimulated Emission Depletion (STED) images of endothelial cells transiently expressing Arf1-GFP (green), infected with non-fluorescent *N. meningitidis*, and fixed. *Left:* Overview of the cell of interest. The yellow line delineates the cell contour. The white dashed line delineates the contour of the bacterial aggregate. Scale bar: 10 µm. Middle and right: Images extracted from the z-stack showing the top (left) and the base (middle) of the bacterial aggregate. The white dashed square denotes the zoomed area shown on the right. Scale bars: 5 and 1 µm, from left to right, respectively. Representative of *n* = 10 infected cells, acquired over *N* = 1 biological replicate. (**F**) Representative images (maximum intensity projections) of endothelial cells transiently expressing Arf1-GFP (green) and infected with iRFP-expressing *N. meningitidis* (shown in gray in the insets) in the presence of 0, 13, or 26 µg/ml of anti-PilE 20D9 antibody (anti-T4P). The white dashed lines delineate the contour of bacterial aggregates. Scale bar: 5 µm. (**G**) Quantification of the percentage of bacterial aggregates recruiting Arf1. Bar graphs show the mean ± SEM. Kruskal–Wallis test was used to assess statistical significance. (**F**,** G**) *n* = 183, 207, and 208 *N. meningitidis*-infected endothelial cells for 0, 13, and 26 µg/ml of 20D9, respectively, pooled from *N* = 3 biological replicates. *P* values are shown on the graph. Source data are available online for this figure.

**Figure EV4:**
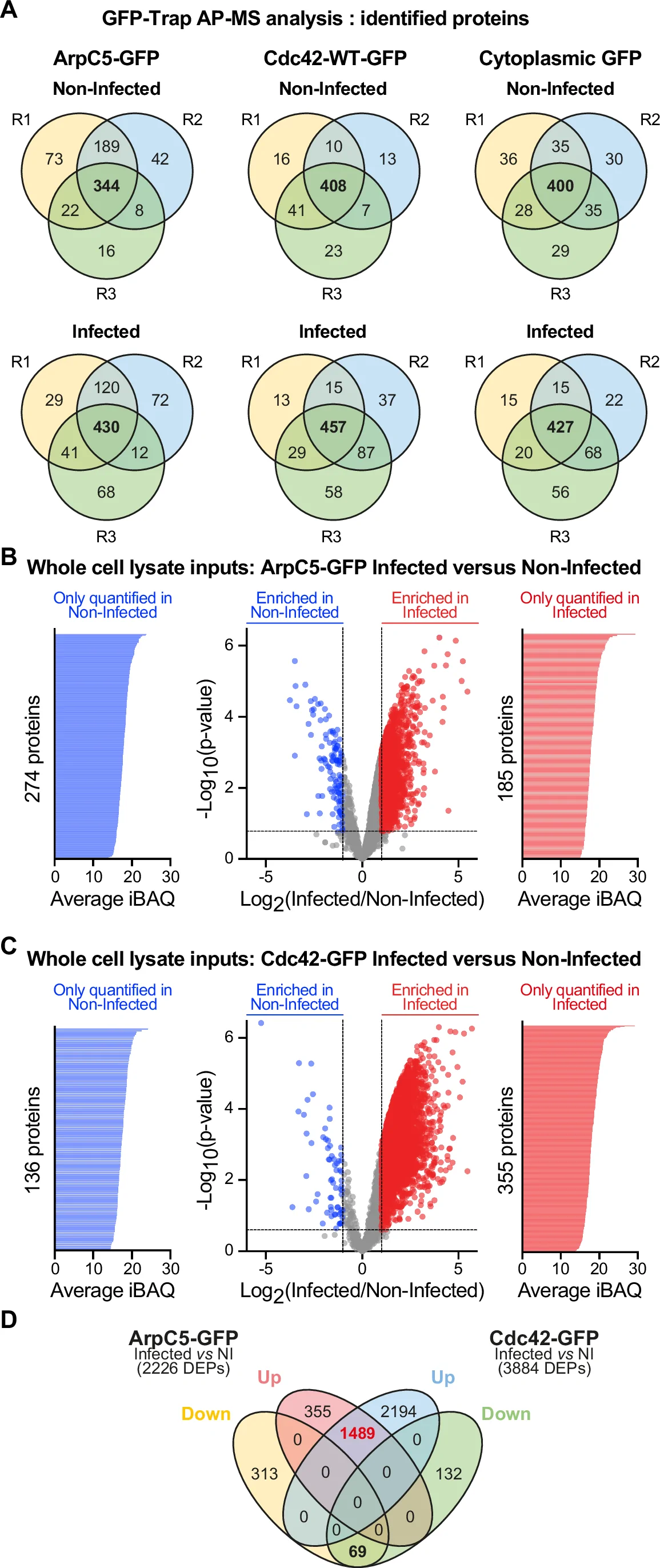
*N. meningitidis* infection modifies the proteomes of endothelial cells. (**A**) Venn diagrams showing the numbers and overlap of the proteins identified in each replicate (R1, R2, and R3) and condition following the GFP-Trap AP-MS analysis in non-infected (top) and *N. meningitidis*-infected (bottom) Arpc5-GFP-, Cdc42-WT-GFP-, or cytoplasmic GFP-expressing endothelial cells. (**B**, **C**) Pairwise comparisons of eukaryotic protein abundance in the whole cell lysates (inputs) of non-infected and *N. meningitidis*-infected ArpC5-GFP- (**B**) or Cdc42-WT-GFP- (**C**) expressing endothelial cells. *Left:* Bar graphs showing the proteins only quantified in the non-infected conditions (blue). Middle: Volcano plots showing the proteins differentially expressed in endothelial cells between non-infected and infected conditions. The horizontal dashed lines represent the threshold used for statistical significance (FDR ≤1%). The vertical dashed lines represent the biological threshold used to select differentially expressed proteins (absolute fold change ≥2). Blue and red dots show proteins whose expression is significantly increased in either the non-infected or the infected conditions, respectively. Right: Bar graphs showing the proteins only quantified in the infected conditions (red). Statistical testing was conducted using the limma *t*-test thanks to the R package limma and an adaptive Benjamini–Hochberg procedure was applied on the resulting *p* values thanks to the function adjust.p of the cp4p R package. (**D**) Venn diagram showing the overlap of the selected proteins (red + blue) among the two pairwise comparisons. (**A**–**D**) Data were obtained from three technical replicates per condition.

**Figure EV5:**
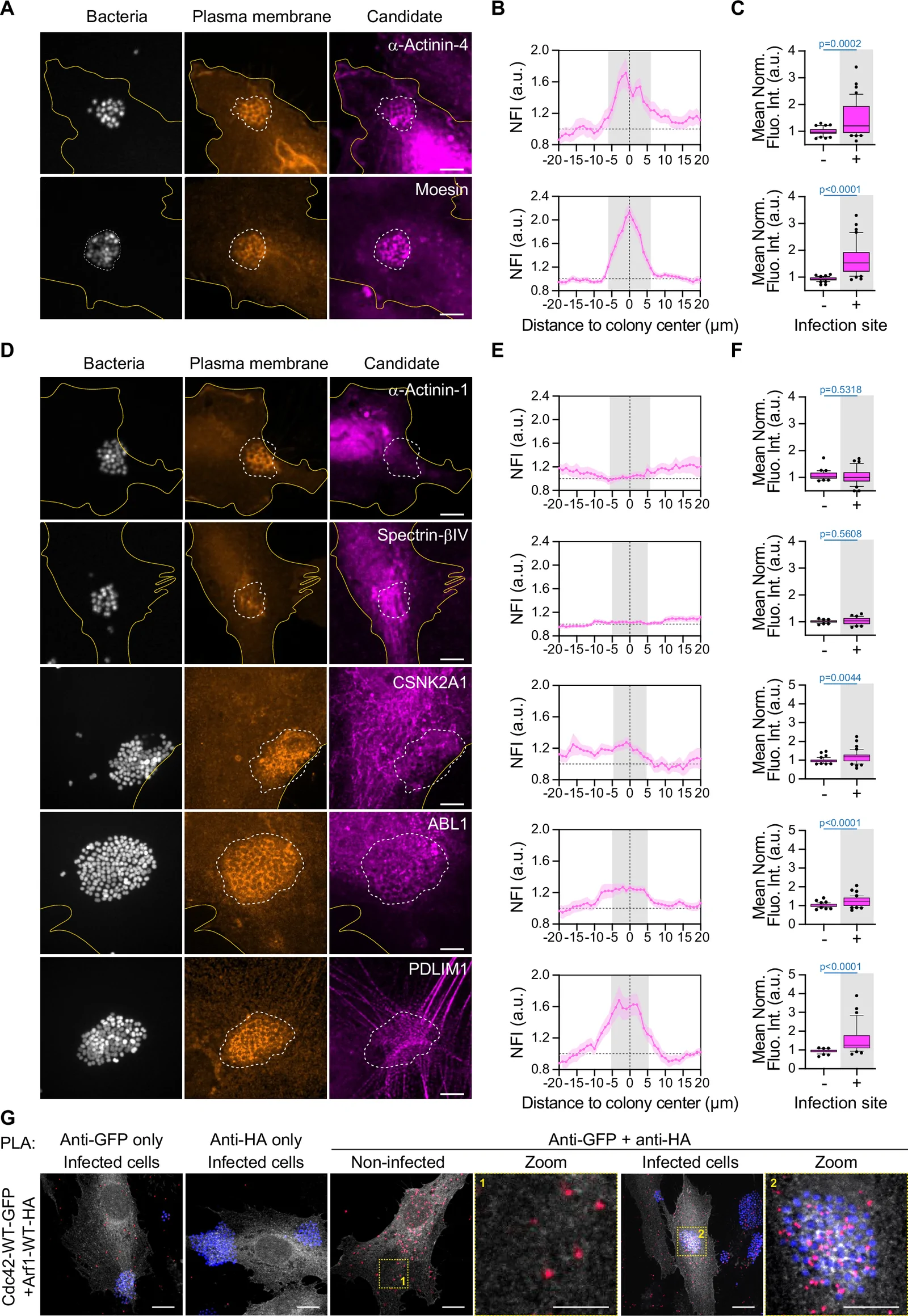
Immunofluorescence analysis of the local enrichment of ArpC5- and Cdc42-interacting protein candidates underneath bacterial microcolonies. (**A**,** D**) Representative images (maximum intensity projections) of endothelial cells transiently expressing the plasma membrane probe PM-GFP (orange), infected with iRFP-expressing *N. meningitidis* (gray) and stained for either endogenous (**A**) α-Actinin-4 or Moesin, and (**D**) α-Actinin-1, Spectrin-βIV, CSNK2A1, ABL1, or PDLIM1 (each using specific antibodies, magenta). The yellow lines delineate the cell contour. The white dashed lines delineate the contour of bacterial aggregates. Scale bars: 5 µm. (**B**,** E**) Normalized fluorescence intensity profiles of (**B**) α-Actinin-4 and Moesin, and (**E**) α-Actinin-1, Spectrin-βIV, CSNK2A1, ABL1, and PDLIM1 in PM-GFP-expressing endothelial cells infected with iRFP-expressing *N. meningitidis*. The light gray areas show the average colony size. Data are shown as mean ± SEM. (**C**,** F**) Mean value per cell of the normalized fluorescence intensity of (**C**) α-Actinin-4 and Moesin, and (**F**) α-Actinin-1, Spectrin-βIV, CSNK2A1, ABL1, and PDLIM1 in the non-infected (−) and infected (+) areas in PM-GFP-expressing endothelial cells infected with iRFP-expressing *N. meningitidis*. Boxes in box plots extend from the 25th to the 75th percentile, with a line at the median, whiskers extend from the 10th to the 90th percentile, and dots show outlier values. The Wilcoxon matched-pairs signed-rank test was used to assess statistical significance. *P* values are shown on the graphs. (**A**–**F**) *n* = 41, 46, 34, 32, 40, 42, and 36 infected endothelial cells for α-Actinin-4, Moesin, α-Actinin-1, Spectrin-βIV, CSNK2A1, ABL1, and PDLIM1, respectively, each pooled from *N* = 2 biological replicates. (**G**) Representative images (maximum intensity projections) of a Proximity Ligation Assay (red) showing the interaction of Cdc42 and Arf1 in endothelial cells transiently co-expressing the wild-type versions of Cdc42 fused to GFP (gray) and Arf1 fused to HA (not displayed), and infected (right) or not (middle right) with iRFP-expressing *N. meningitidis* (blue). Control conditions using only one of the primary antibodies are shown on the middle left (anti-HA) and left (anti-GFP) panels, respectively. The yellow dashed squares delineate the zoomed areas in non-infected (1) and infected (2) cells shown on the right of the corresponding image. Scale bars: 10 and 5 µm, respectively. Representative of *n* = 30 cells per condition, pooled from *N* = 2 biological replicates.

So far, our results show the instrumental role played by Cdc42 in regulating the Arp2/3-dependent nucleation of a branched F-actin network in endothelial protrusions formed in response to plasma membrane deformation by *N. meningitidis*. We next sought to gain insight into molecular players regulating the downstream or upstream activity of both Arp2/3 and Cdc42. We therefore developed a quantitative affinity purification–mass spectrometry (AP-MS) approach to identify proteins differentially interacting with either Arp2/3 or Cdc42 in meningococcal-infected endothelial cells, using non-infected cells as controls. To this end, endothelial cells transiently expressing either ArpC5-GFP or Cdc42-WT-GFP were infected, whole cell lysates (inputs) were prepared, and ArpC5- and Cdc42-WT-GFP-interacting proteins were co-immunoprecipitated using a GFP-pull-down approach, eluted and analyzed by high-resolution mass spectrometry, each condition performed in biological triplicates (Figs. 7A and EV4A). We next used the SAINTexpress (significance analysis of INTeractome) software (Teo et al, 2014) to score protein–protein interactions and define the respective interactomes of ArpC5 and Cdc42 in infected and non-infected endothelial cells, using the corresponding cytoplasmic GFP-expressing cells as controls. Noticeably, 57 and 31 proteins were respectively found to specifically interact with ArpC5 and Cdc42 in infected endothelial cells relative to non-infected cells (Figs. 7B, red lines and EV4B–D; Datasets EV1–3), among which nine were found to interact with both baits. No variation in the association of either the different subunits (ACTR2, ACTR3, ARPC1A, ARPC1B, ARPC2, ARPC3, ARPC4) of the Arp2/3 complex with ArpC5-GFP or known interactors (IQGAP1 and CALM3) of Cdc42 was observed between non-infected and infected endothelial cells (Fig. 7B; Dataset EV3). Interestingly, proteins previously shown to be recruited underneath *N. meningitidis* microcolonies, such as α-actinin-4 (ACTN4) and Moesin (MSN) (Maissa et al, 2017), were also identified and their presence confirmed by immunofluorescence staining (Fig. EV5A–C), hence supporting the robustness of our approach. These results strongly suggest that meningococcal infection induces the specific assembly of a multiproteic complex that encompasses Cdc42 and Arp2/3 to locally regulate the assembly of a branched F-actin network in *N. meningitidis*-induced endothelial tubular protrusions.

### Arf1 is required for the proper nucleation of branched F-actin in endothelial tubular protrusions induced by *N. meningitidis*

We then searched for the presence of Arp2/3- and Cdc42-interacting proteins known, based on their described function in the literature, to either link the plasma membrane to the actin cytoskeleton or crosslink actin filaments and investigated the potential involvement of six candidates: Spectrin-βIV (SPTNB4), α-actinin-1 (ACTN1), PDZ and LIM domain protein 1 (PDLIM1), a known α-actinins-interacting protein (Bauer et al, 2000; Vallenius et al, 2000); the small GTPase Arf1, previously described to regulate Arp2/3-mediated branched actin assembly through Cdc42 (Dubois et al, 2005; Heuvingh et al, 2007); the tyrosine-protein kinase ABL1, and the Casein Kinase 2 α1 (CSNK2A1) (Fig. 7B, circled in magenta). While we were not able to validate the local enrichment of Spectrin-βIV, α-actinin-1, and Casein Kinase 2 α1, immunofluorescence analysis confirmed the local enrichment of PDLIM1 and, to a lesser extent, ABL1, together with the plasma membrane, underneath bacterial microcolonies (Fig. EV5D–F). The local accumulation of Arf1 in *N. meningitidis*-induced endothelial protrusions was confirmed by immunofluorescence analysis following the transient expression of a GFP-tagged version of the protein in endothelial cells prior to cell infection (Fig. 7C,D). Similarly, Arf1 interaction with Cdc42 was confirmed by immunofluorescence using a proximity ligation assay (PLA) (Hegazy et al, 2020) in endothelial cells transiently co-expressing wild-type versions of a GFP-tagged Cdc42 and HA-tagged Arf1 (Fig. EV5G). In agreement with its well-described function at the Golgi apparatus (Adarska et al, 2021), we found that most of the Arf1-GFP signal colocalized with this organelle, but a small fraction of the protein was detected in endothelial protrusions upon adhesion of meningococci (Fig. 7C,D). We next used STimulated emission depletion (STED) microscopy (Hell and Wichmann, 1994) to perform high-resolution imaging of the pool of protrusion-associated Arf1-GFP and confirmed its association with the plasma membrane rather than intracellular vesicles (Fig. 7E). Importantly, the local enrichment of Arf1 within *N. meningitidis*-induced endothelial tubular protrusions was impaired in a dose-dependent manner when infection experiments were conducted in the presence of increasing concentrations of the function-blocking 20D9 monoclonal antibody directed against *N. meningitidis* type IV pili (Pujol et al, 1999 ; Charles-Orszag et al, 2018) (Fig. 7F,G). Arf1 recruitment is therefore triggered by type IV pili.

**Figure 8:**
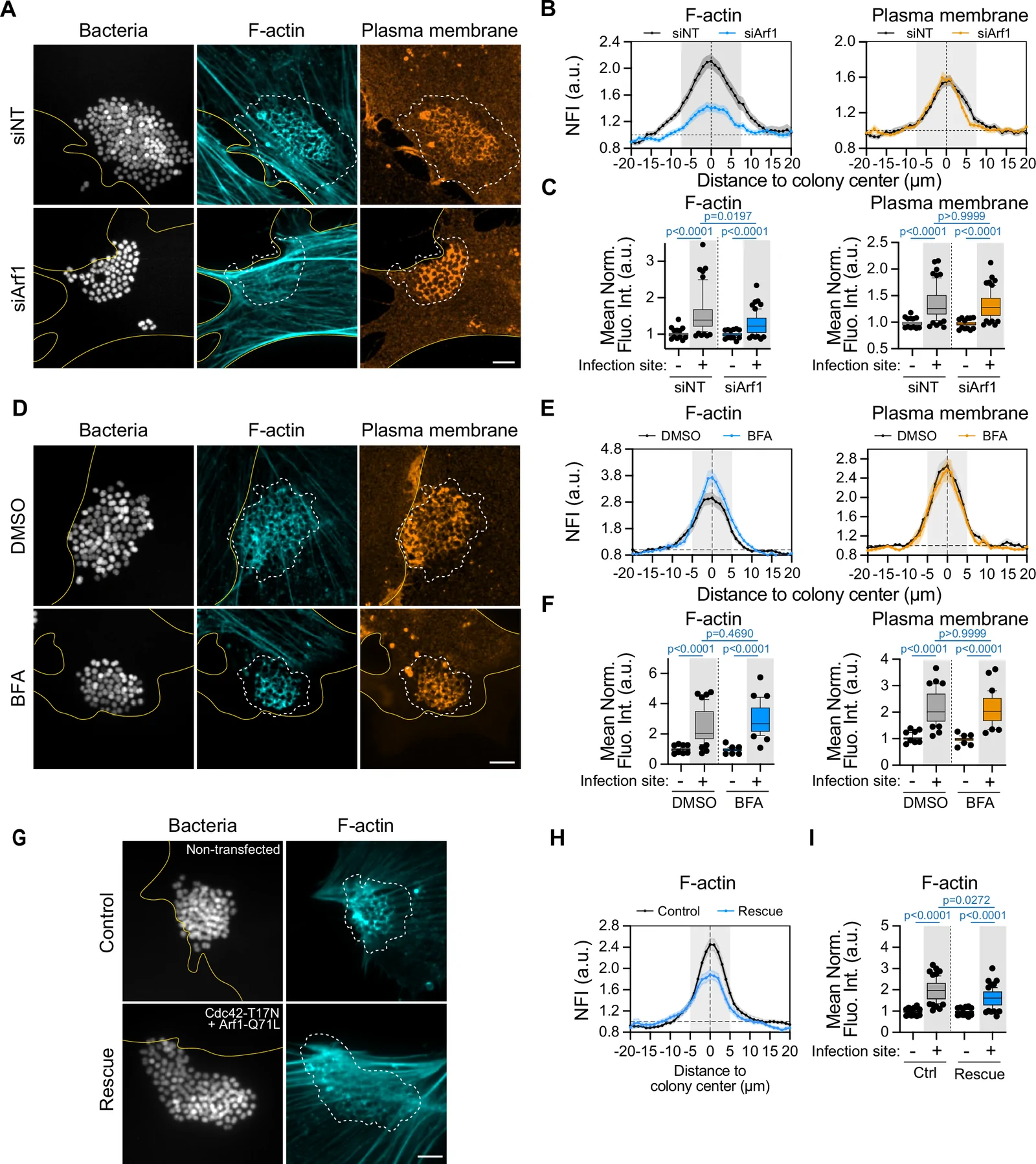
Arf1 inhibition impairs F-actin reorganization in endothelial tubular protrusions formed following *N. meningitidis*-induced plasma membrane deformation. (**A**) Representative images (maximum intensity projections) of control (siNT) and Arf1 (siArf1)-silenced endothelial cells infected with iRFP-expressing *N. meningitidis* (gray) and stained for F-actin (phalloidin, cyan) and the plasma membrane (CD31, orange). The yellow lines delineate the cell contour. The white dashed lines delineate the contour of bacterial aggregates. Scale bar: 5 µm. (**B**) Normalized fluorescence intensity profiles of F-actin and the plasma membrane in control (siNT) and Arf1 (siArf1)-silenced endothelial cells infected with iRFP-expressing *N. meningitidis*. The light gray areas show the average colony size. Data were shown as mean ± SEM. (**C**) Mean value per cell of the normalized fluorescence intensity of F-actin and the plasma membrane in the non-infected (−) and infected (+) areas in control (siNT) and Arf1 (siArf1)-silenced endothelial cells infected with iRFP-expressing *N. meningitidis*. The Kruskal–Wallis test was used to assess statistical significance. (**A**–**C**) *n* = 68 and 60 infected cells for siNT and siArf1, respectively, pooled from *N* = 2 biological replicates. (**D**) Representative images (maximum intensity projections) of endothelial cells infected with iRFP-expressing *N. meningitidis* (gray), treated prior to and during infection with either control (DMSO) or Brefeldin A (BFA) and stained for F-actin (phalloidin, cyan) and the plasma membrane (CD31, orange). The yellow lines delineate the cell contour. The white dashed lines delineate the contour of bacterial aggregates. Scale bar: 5 µm. (**E**) Normalized fluorescence intensity profiles of F-actin and the plasma membrane in endothelial cells treated with either DMSO or BFA and infected with iRFP-expressing *N. meningitidis*. The light gray areas show the average colony size. Data were shown as mean ± SEM. (**F**) Mean value per cell of the normalized fluorescence intensity of F-actin and the plasma membrane in the non-infected (−) and infected (+) areas in endothelial cells treated with either DMSO or BFA and infected with iRFP-expressing *N. meningitidis*. Kruskal–Wallis test was used to assess statistical significance. (**D**–**F**) *n* = 40 and 35 infected endothelial cells for DMSO and BFA, respectively, pooled from *N* = 2 biological replicates. (**G**) Representative images (maximum intensity projections) of control (non-transfected) and endothelial cells transiently co-expressing a dominant negative mutant (T17N) version of Cdc42 fused to GFP and a constitutive active mutant (Q71L) version of Arf1 fused to HA tag (rescue), infected with iRFP-expressing *N. meningitidis* (gray), and stained for F-actin (phalloidin, cyan). The yellow lines delineate the cell contour. The white dashed lines delineate the contour of bacterial aggregates. Scale bar: 5 µm. (**H**) Normalized fluorescence intensity profiles of F-actin in control or Cdc42-T17N and Arf1-Q71L-co-expressing (rescue) endothelial cells infected with iRFP-expressing *N. meningitidis*. The light gray areas show the average colony size. Data were shown as mean ± SEM. (**I**) Mean value per cell of the normalized fluorescence intensity of F-actin in the non-infected (−) and infected (+) areas in control and Cdc42-T17N and Arf1-Q71L-co-expressing (rescue) endothelial cells infected with iRFP-expressing *N. meningitidis*. The Kruskal–Wallis test was used to assess statistical significance. (**G**–**I**) *n* = 69 and 69 infected endothelial cells for control and Cdc42-T17N + Arf1-Q71L co-expression (rescue), respectively, pooled from *N *= 2 biological replicates. (**C**,** F**,** I**) Boxes in box plots extend from the 25th to the 75th percentile, with a line at the median, whiskers extend from the 10th to the 90th percentile, and dots show outlier values. *P* values are shown on the graphs. Source data are available online for this figure.

**Figure EV6:**
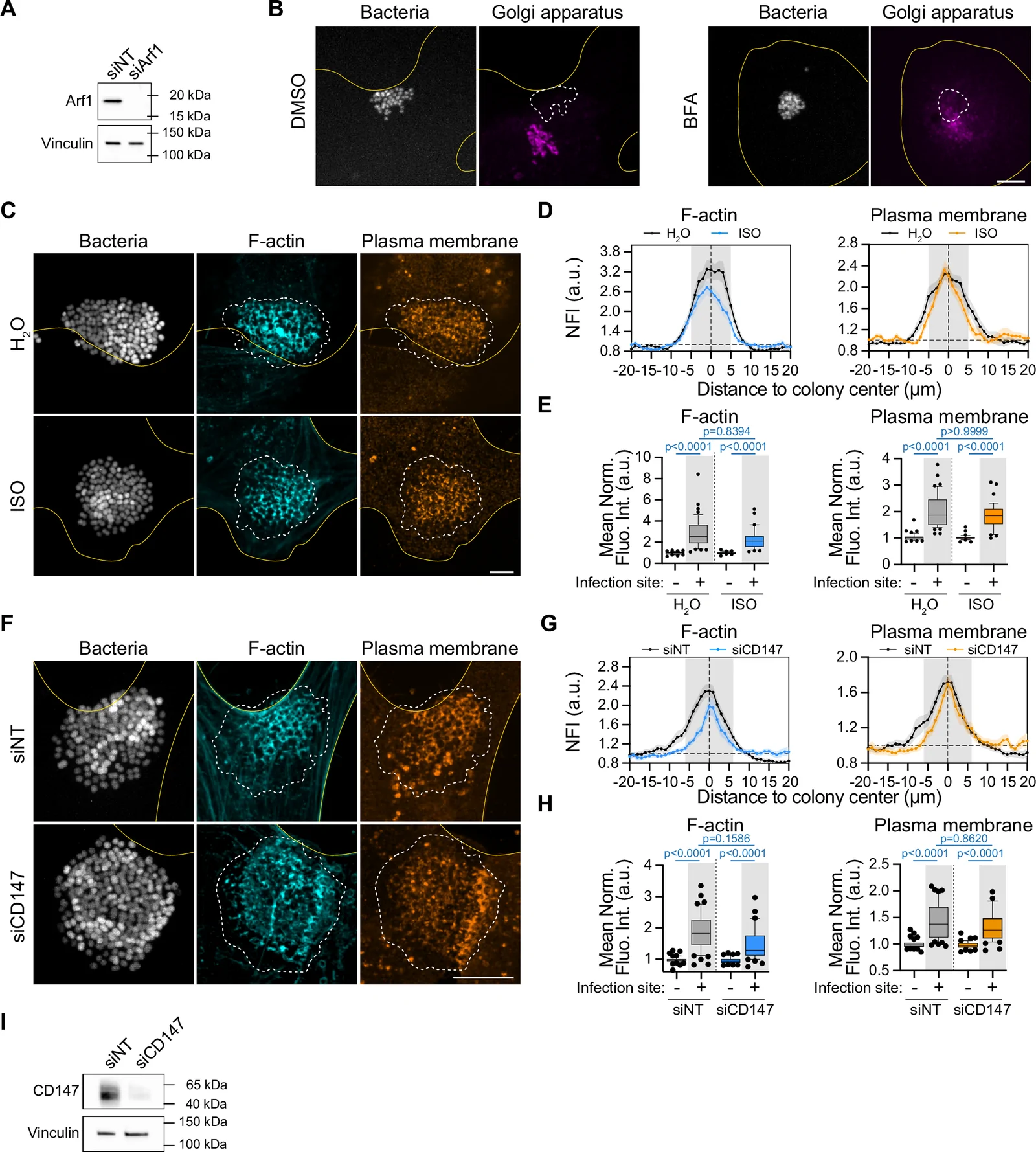
The Arf1-Cdc42-N-WASP-Arp2/3 axis is not induced upon the activation of the β2-AR and CD147 cell-surface receptors. (**A**) Western blot analysis of the efficiency of Arf1 silencing. Vinculin was used as a loading control. The immunoblots shown are representative of *N* = 2 biological replicates. (**B**) Representative images (maximum intensity projections) of endothelial cells infected with iRFP-expressing *N. meningitidis* (gray), treated prior and during infection with either control (DMSO) or Brefeldin A (BFA), and stained for the Golgi apparatus (GM130, magenta). The yellow lines delineate the cell contour. The white dashed lines delineate the contour of bacterial aggregates. Scale bar: 10 µm. Representative of *n* = 32 infected cells, pooled from *N* = 2 biological replicates. (**C**) Representative images (maximum intensity projections) of endothelial cells infected with iRFP-expressing *N. meningitidis* (gray), treated prior and during infection with either H_2_O (control) or Isoproterenol (ISO), and stained for F-actin (phalloidin, cyan) and the plasma membrane (CD31, orange). The yellow lines delineate the cell contour. The white dashed lines delineate the contour of bacterial aggregates. Scale bar: 5 µm. (**D**) Normalized fluorescence intensity profiles of F-actin and the plasma membrane in endothelial cells treated with either H_2_O or ISO and infected with iRFP-expressing *N. meningitidis*. The light gray areas show the average colony size. Data were shown as mean ± SEM. (**E**) Mean value per cell of the normalized fluorescence intensity of F-actin and the plasma membrane in the non-infected (−) and infected (+) areas in endothelial cells treated with either H_2_O or ISO and infected with iRFP-expressing *N. meningitidis*. The Kruskal–Wallis test was used to assess statistical significance. (**C**–**E**) *n* = 48 and 36 infected endothelial cells for H_2_O and ISO, respectively, pooled from *N* = 2 biological replicates. (**F**) Representative images (maximum intensity projections) of control (siNT) and CD147 (siCD147)-silenced endothelial cells infected with iRFP-expressing *N. meningitidis* (gray), and stained for F-actin (phalloidin, cyan) and the plasma membrane (CD31, orange). The yellow lines delineate the cell contour. The white dashed lines delineate the contour of bacterial aggregates. Scale bar: 5 µm. (**G**) Normalized fluorescence intensity profiles of F-actin and the plasma membrane in control (siNT) and CD147 (siCD147)-silenced endothelial cells infected with iRFP-expressing *N. meningitidis*. The light gray areas show the average colony size. Data were shown as mean ± SEM. (**H**) Mean value per cell of the normalized fluorescence intensity of F-actin and the plasma membrane in the non-infected (−) and infected (+) areas in control (siNT) and CD147 (siCD147)-silenced endothelial cells infected with iRFP-expressing *N. meningitidis*. Kruskal–Wallis test was used to assess statistical significance. (**F**–**H**) *n* = 51 and 42 infected cells for siNT and siCD147, respectively, pooled from *N* = 2 biological replicates. (**E**,** H**) Boxes in box plots extend from the 25th to the 75th percentile, with a line at the median, whiskers extend from the 10th to the 90th percentile, and dots show outlier values. *P* values are shown on the graphs. (**I**) Western blot analysis of the efficiency of CD147 silencing. Vinculin was used as a loading control. The immunoblots shown are representative of *N* = 2 biological replicates.

To assess the role of these three proteins (PDLIM1, ABL1, and Arf1) in locally regulating the Arp2/3-mediated reorganization of the actin cytoskeleton in response to the formation of tubular membrane protrusions in endothelial cells, we next sought to silence their expression individually using siRNA. We were not able to achieve efficient silencing of PDLIM1 and ABL1 in our primary endothelial cells, but the efficient silencing of Arf1 expression drastically impaired F-actin enrichment underneath bacterial microcolonies (Figs. 8A–C and EV6A). Importantly, according to its subcellular localization and function, Arf1 activity is differentially affected by cell treatment with the chemical inhibitor Brefeldin A (BFA). While sensitive to BFA at the Golgi apparatus, where it participates in vesicle exocytosis, its role in promoting CLIC/GEEC-mediated endocytosis at the plasma membrane is not (Kumari and Mayor, 2008; Sathe et al, 2021). In agreement with a direct role of Arf1 at the plasma membrane in our conditions, treating endothelial cells with BFA did not impair F-actin reorganization within *N. meningitidis*-induced plasma membrane protrusions (Fig. 8D–F), while inducing the disassembly of the Golgi apparatus (Fig. EV6B). Having shown that the pool of plasma membrane-associated Arf1 locally regulates F-actin reorganization within *N. meningitidis*-induced tubular protrusions in endothelial cells, we next sought to determine whether Arf1 acted upstream or downstream of Cdc42. We therefore performed rescue experiments by co-transfecting endothelial cells with a constitutive active mutant of Arf1 (Arf1-Q71L) (Furman et al, 2002) together with the dominant negative mutant version of Cdc42 (Cdc42-T17N) used in Fig. 5D–F. No rescue was observed, and F-actin levels remained low underneath bacterial microcolonies (Fig. 8G–I), supporting the notion that Arf1 acts upstream of Cdc42 in regulating the N-WASP-dependent activation of the Arp2/3 complex in *N. meningitidis*-induced plasma membrane tubular protrusions.

To further our understanding of the early sequence of events leading to Arf1 activation at the plasma membrane, we next assessed the involvement of the two previously described meningococcal receptors, the β2 adrenergic receptor (β2-AR) and CD147 (Bernard et al, 2014; Coureuil et al, 2010). Treatment of endothelial cells with the β2-AR agonist isoproterenol to induce its endocytosis (Coureuil et al, 2010; Soyer et al, 2014) and siRNA-mediated silencing of CD147 led to a slight decrease in F-actin enrichment in *N. meningitidis*-induced endothelial protrusions, although it did not reach statistical significance in the conditions used here. No difference in plasma membrane accumulation was observed (Fig. EV6C–I). The reason for the discrepancy with previous publications (Bernard et al, 2014; Coureuil et al, 2010) remains unknown at this stage, especially considering the high efficiency of CD147 silencing (Fig. EV6I), but it should be pointed out that different cell types were used.

Taken together, these results strongly suggest that the two small GTPases Arf1 and Cdc42 interact upon *N. meningitidis* adhesion to the surface of endothelial cells and cooperate to regulate the N-WASP-dependent activation of the Arp2/3 complex, hence supporting the local assembly of a branched F-actin network in endothelial tubular plasma membrane protrusions.

## Discussion

Cellular plasma membranes are frequently deformed to support a broad range of biological processes. In such deformations, the reorganization of the actin cytoskeleton can either promote membrane remodeling (Le Clainche and Carlier, 2008; Ridley and Heald, 2011) or occur subsequently (Charles-Orszag et al, 2018; Charras and Paluch, 2008; Datar et al, 2015). Here, we first show that deformation of the plasma membrane upon *N. meningitidis* adhesion at the surface of epithelial or endothelial cells triggers the formation of morphologically distinct tubular membrane protrusions that harbor cell type-specific F-actin networks. Epithelial protrusions were enriched in linear actin filaments, strongly suggesting a role for a member of the Formin family of actin nucleators in this cell type (Chesarone et al, 2010; Chesarone and Goode, 2009). Unexpectedly and in sharp contrast, we show that endothelial protrusions rather enclose an Arp2/3-mediated branched F-actin network, whose assembly relies on the local activation of the small GTPases Arf1 and Cdc42 and the nucleation-promoting factor N-WASP. Taken together, our results argue for the existence of cell type-specific mechanisms to regulate the reorganization of the cortical F-actin network in *N. meningitidis*-induced endothelial tubular membrane protrusions.

Although induced by external mechanical forces in both cell types, the assembly of F-actin networks of distinct ultrastructural organization in epithelial and endothelial cells sheds light on cell-intrinsic properties that might have important pathophysiological implications. First, this suggests that while the pool of cortical F-actin in both epithelial and endothelial cells mainly relies on Arp2/3 activity, these two cell types mobilize distinct F-actin nucleating machineries in response to mechanical membrane deformation by 1DW. Interestingly, these observations, although consistent with the literature (Lecuyer et al, 2012), might reflect a more general mechanism by which a given cell type will preferentially utilize either Arp2/3- or Formin-dependent actin nucleation machineries in response to membrane deformation. In agreement with this hypothesis, while epithelial cells form actin bundle-rich microvilli at their apical surface (Sauvanet et al, 2015), endothelial cells rather assemble branched F-actin-dependent lamellipodium-like structures, referred to as dactylopodia (Eilken and Adams, 2010; Figueiredo et al, 2021). Whether and how these intrinsic capacities of epithelial and endothelial cells to mobilize distinct actin nucleation machineries is linked to their ability to assemble cell type-specific F-actin networks following the formation of tubular plasma membrane protrusions by 1DW represents an interesting path for future investigations. Similarly, whether the assembly of cell type-specific F-actin networks confers distinct properties to such protrusions in epithelial versus endothelial cells, by differentially influencing the dynamics of protrusion elongation and/or stabilization, should now be addressed to fully appreciate the role of these membrane protrusions in various pathophysiological contexts.

We show that in *N. meningitidis*-induced endothelial tubular protrusions, the small GTPase Cdc42 and the nucleation-promoting factor N-WASP locally cooperate to promote Arp2/3 activation and the assembly of a branched F-actin network. In addition, we identified the membrane-binding small GTPase Arf1 (ADP-ribosylation factor 1) as an interactor of Cdc42 in *N. meningitidis*-infected endothelial cells. It should be pointed out that the interaction between Arf1 and Cdc42 is unlikely to be direct. These proteins have been shown to interact through intermediate proteins, coatomer proteins or through ARHGAP21 (Myers and Casanova, 2008). Further studies will be required to define the composition of the complex, but the COPIB protein is found in our pull-downs, making the coatomer an interesting candidate.

Several lines of evidence implicate Arf1 in meningococci-induced actin reorganization: (i) the pull-down experiment itself demonstrated that meningococcal infection triggers Cdc42/Arf1 interaction; (ii) Arf1 is localized under the colonies as demonstrated by high-resolution imaging, and (iii) siRNA-mediated silencing of Arf1 strongly reduces F-actin accumulation under meningococcal microcolonies. Arf1 is best known for its role at the Golgi apparatus (Adarska et al, 2021), where it regulates the dynamics of Arp2/3-dependent F-actin network through Cdc42 (Dubois et al, 2005). More importantly, in the context of our study, a small proportion of Arf1 has also been found to interact with the plasma membrane, where it locally regulates the Cdc42-dependent clathrin-independent (CLIC/GEEC) endocytic pathway (Kumari and Mayor, 2008). It is therefore conceivable that Arf1 enrichment at the plasma membrane underneath the bacterial microcolonies allows the local accumulation of Cdc42, which in turn regulates Arp2/3 activity through N-WASP activation. These results reveal a striking parallel between the F-actin reorganization elicited by meningococci and the one associated with the CLIC/GEEC clathrin-independent endocytic pathway. In both cases, the same signaling cascade, comprising Arf1, Cdc42, N-WASP, and the Arp2/3 complex, drives actin dynamics. A hallmark common to the two processes is their dependence on a plasma membrane pool of Arf1 that operates independently of Brefeldin A sensitive Golgi-associated Arf1.

Interestingly, our interactome analysis revealed ABL1 and PDLIM1 as other potential Cdc42-interacting proteins in infected endothelial cells and confirmed their local enrichment underneath bacterial microcolonies by immunofluorescence. However, for technical limitations, we could not explore their functional involvement in regulating the Arp2/3-dependent reorganization of F-actin in *N. meningitidis*-induced tubular membrane protrusions in endothelial cells. ABL1 is a non-receptor tyrosine kinase. Given its known involvement in many different processes, the role of this kinase in meningococci-induced actin reorganization is intriguing but will require specific investigation. Importantly, ABL1 phosphorylates numerous proteins that are involved in cytoskeletal dynamics, cell adhesion and motility, all of which could be at play here (Bradley and Koleske, 2009). PDLIM1 is also a modulator of the actin cytoskeleton through its activity as an adapter protein (Zhou et al, 2021). Interestingly, PDLIM1 binds to ACTN4 which was also found in the pull-downs. Our results thus reveal a complex signaling hub of actin dynamics modulators.

The formation of plasma membrane protrusions by 1DW is also likely to occur in conditions other than those of *N. meningitidis* infection, as we recently observed upon the interaction of endothelial cells with the extracellular matrix (Charles-Orszag et al, 2018). Indeed, the extracellular matrix is composed of a network of adhesive fibers of various sizes, reaching sizes down to 5 nm in diameter (Frantz et al, 2010; Vakonakis and Campbell, 2007), therefore fully compatible with the regime of membrane deformation by 1DW. It is therefore tempting to envision that the formation of membrane protrusions by 1DW might represent a general mechanism used by cells to generate thin membrane protrusions in response to their interaction with the microenvironment. Interestingly, it was recently shown that WASP, a N-WASP homolog specifically expressed in cells of the hematopoietic lineage, is a substrate topology sensor (Brunetti et al, 2022). WASP locally accumulates at sites of substrate-induced membrane deformations where it promotes the assembly of branched Arp2/3-dependent F-actin in migrating neutrophils. In this context, one could envision that the formation of plasma membrane protrusions by 1DW might represent the initial cellular response following the sensing of adhesive nanofibers. In the case of *N. meningitidis* infection, this would lead to the stabilization of the bacterial microcolony at the apical surface of the host cells, allowing bacteria to resist high levels of shear stress (Mikaty et al, 2009). Beyond infection, the formation of plasma membrane protrusions by 1DW in response to the interaction of cells with nanofiber-containing extracellular matrices might represent the early step leading to dactylopodia and/or filopodium/invadopodium establishment, hence promoting cell migration, adhesion, and space exploration. In this context, one could envision that, similarly to WASP in neutrophils (Brunetti et al, 2022), endothelial N-WASP might act as a substrate topology sensor that would be activated downstream of endothelial cell contacts with adhesive nanofibers, either meningococcal T4P or the ones found in the extracellular matrix.

Our results thus point to the existence of an intracellular pathway linking mechanical plasma membrane deformation and subsequent actin reorganization, which takes place during several biological processes, including CLIC/GEEC endocytosis, topology sensing and bacterial infection.

## Methods

**Table:** Reagents and tools table

Reagent/resource	Reference or source	Identifier or catalog number
**Experimental models**		
Primary human umbilical vein endothelial cells (HUVECs)	Lonza	#C2519A
hTERT-immortalized HUVECs	Kindly provided by Arnold Hayer and described in (Hayer et al, 2016)	
hTERT-immortalized HUVECs FTractin	Kindly provided by Arnold Hayer and described in (Hayer et al, 2016)	
Human nasal epithelial cells (HNEpCs)	PromoCell	#C-12620
*Neisseria meningitidis* strain 8013 clone 12 (serogroup C clinical isolate)	Described in (Nassif et al, 1993; Rusniok et al, 2009)	
BFP-expressing *Neisseria meningitidis* strain	Described in (Charles-Orszag et al, 2018)	
GFP-expressing *Neisseria meningitidis* strain	Described in (Charles-Orszag et al, 2018)	
iRFP-expressing *Neisseria meningitidis* strain	Described in (Charles-Orszag et al, 2018)	
**Recombinant DNA**		
PM-EGFP	Kindly provided by Barbara Baird and David Holowka and described in (Bag et al, 2021)	
ArpC5-GFP	Kindly provided by Philipe Chavrier	
pcDNA3-EGFP-Rac1(WT)	Addgene	#13719
pCDNA3-EGFP-Rac1(T17N)	Addgene	#13721
pcDNA3-EGFP-Cdc42(wt)	Addgene	#12599
pcDNA3-EGFP-Cdc42(T17N)	Addgene	#12601
mRFP-wGBD	Addgene	#26733
pMAG201-EGFP-Arf1	Kindly provided by Cathy Jackson	
pcDNA3 HA-Arf1	Addgene	#10830
pcDNA3 HA-Arf1 ActQ71L	Addgene	#10832
pEGFP-C3	Kindly provided by Sandrine Etienne-Manneville and described in (Pyenta et al, 2001)	
**Antibodies**		
Mouse anti-CD31/PECAM-1	BioLegend	#303102
Rabbit anti-CD326/EpCAM	Abcam	#ab223582
Rabbit anti-Arp2	Abcam	#ab47654
Mouse anti-Arp3	Sigma-Aldrich	#A5979
Rabbit anti-N-WASP	Abcam	#ab126626
Rabbit anti-WAVE1	Abcam	#ab272916
Rabbit anti-Moesin	Abcam	#ab52490
Rabbit anti-Spectrin β IV	Thermo Fisher Scientific	#MA5-27608
Rabbit anti-α-actinin-1	Proteintech	#11313-2-AP
Rabbit anti-α-actinin-4	Proteintech	#19096-1-AP
Mouse anti-c-Abl	Thermo Fisher Scientific	#MA5-14398
Rabbit anti-CSNK2A1	Proteintech	#10992-1-AP
Rabbit anti-PDLIM1/CLP36	Proteintech	#11674-1-AP
Mouse anti-GM130	BD Transduction Laboratories	#610822
Rabbit anti-GFP	Abcam	#ab290
Rabbit anti-GFP	Thermo Fisher Scientific	##50430-2-AP
Mouse anti-HA.11 Epitope Tag	BioLegend	#901501
Mouse anti-PilE 20D9	Described in (Pujol et al, 1999)	
Rabbit anti-Cdc42	Abcam	#ab187643
Rabbit anti-Cdc42	Cell Signaling Technology	#2462
Rabbit anti-Arf1	Proteintech	#20226-1-AP
Rabbit anti-CD147	Proteintech	#11989-1-AP
Mouse anti-Vinculin	Sigma-Aldrich	#V9264
Chicken anti-GFP	Abcam	#ab13970
AlexaFluor488-conjugated goat anti-rabbit	Thermo Fisher Scientific	#A11008
AlexaFluor568-conjugated goat anti-rabbit	Thermo Fisher Scientific	#A11011
AlexaFluor488-conjugated goat anti-mouse	Thermo Fisher Scientific	#A21121
AlexaFluor568-conjugated goat anti-mouse	Thermo Fisher Scientific	#A11004
STAR RED anti-rabbit	Abberior	#STRED-1002-500UG
HRP-conjugated donkey anti-rabbit	Jackson ImmunoResearch	#111-035-144
HRP-conjugated donkey anti-mouse	Jackson ImmunoResearch	#115-035-146
HRP-conjugated donkey anti-chicken	Jackson ImmunoResearch	#703-035-155
**Oligonucleotides and other sequence-based reagents**		
ON-TARGETplus Non-Targeting pool (NT)	Horizon Discovery	#D-001810-10-05
ON-TARGETplus Human SMARTpool ACTR2 (Arp2)	Horizon Discovery	#L-012076-02-0005
ON-TARGETplus Human SMARTpool ACTR3 (Arp3)	Horizon Discovery	#L-012077-00-0005
ON-TARGETplus Human SMARTpool WASL (N-WASP)	Horizon Discovery	#L-006444-00-0005
ON-TARGETplus Human SMARTpool Arf1	Horizon Discovery	#L-011580-00-0005
ON-TARGETplus Human SMARTpool BSG (CD147)	Horizon Discovery	#L-010737-00-0005
**Chemicals, enzymes, and other reagents**		
AlexaFluorPlus405-conjugated Phalloidin	Thermo Fisher Scientific	#A30104
AlexaFluor488-conjugated Phalloidin	Thermo Fisher Scientific	#A12379
AlexaFluor568-conjugated Phalloidin	Thermo Fisher Scientific	#A12380
AlexaFluor647-conjugated Phalloidin	Thermo Fisher Scientific	#A22287
DyLight649-labeled Ulex Europaeus Agglutinin I lectin (UEA-1)	Vector Laboratories	#DL-1068
BioTracker655 Red Cytoplasmic Membrane Dye	Sigma-Aldrich	#SCT108
OptiMEM I Reduced Serum Medium	Thermo Fisher Scientific	#10149832
Lipofectamine RNAiMAX	Thermo Fisher Scientific	#13778075
CK689	Sigma-Aldrich	#182517
CK666	Tocris	#3950
CK312	Sigma-Aldrich	#182518
CK869	Sigma-Aldrich	#182516
SMIFH2	Tocris	#4401
Brefeldin A	Sigma-Aldrich	#B6542
Isoproterenol	Sigma-Aldrich	#PHR2722
Hygromycin B	Euromedex	#EU3250
Paraformaldehyde 16%	EMS	#EM-15710
Fluoromont-G	Southern Biotech	#0100-01
Mowiol	Sigma-Aldrich	
Triton X-100	Sigma-Aldrich	#T8787
Fish Gelatin	Sigma-Aldrich	#G7041
RIPA		
cOmplete EDTA-free Protease Inhibitor Cocktail Tablets	Sigma-Aldrich	#11873580001
Tween 20	Thermo Fisher Scientific	#13464259
Rat Collagen Type I	Sigma-Aldrich	#122-20
Glutaraldehyde	EMS	#16220
Osmium tetroxide 2%	EMS	#19152
Ethanol	Sigma-Aldrich	#51976-500ML-F
Potassium Ferrocyanide	Sigma-Aldrich	
Tannic acid	Sigma-Aldrich	
Osmium	EMS	
Uranyl Acetate	EMS	
Epoxy resin	EMS	
Endothelial Cell Growth Medium (EGM)-2	Lonza	#CC-3162
Airway Epithelial Cell Growth Medium (AEGM)	PromoCell	#C-21060
Trypsine/EDTA	Sigma-Aldrich	#T3924
Sequencing Grade Modified Trypsin	Promega	
**Software**		
MetaMorph (version 7.10.4.407)	Molecular Device	
ZEN Black	ZEISS	
ZEN Blue	ZEISS	
Open-source Fiji (ImageJ)	Schindelin et al, 2012	
Open-source Icy	de Chaumont et al, 2012	
GraphPad Prism 11	GraphPad	
ATLAS 3D	ZEISS	
Amira (version 2020.2 to 2022.1)	Thermo Fisher Scientific	
MAPS (version 3.17)	Thermo Fisher Scientific	
AutoTEM (version 2.2.0)	Thermo Fisher Scientific	
SerialEM (version 3.7 to 4.1)	Described in (RRID:SCR_017293)	
IMOD (version 4.9.10)	Described in (RRID:SCR_003297)	
Xcalibur	Thermo Fisher Scientific	
MaxQuant (version 2.1.1.0)	Described in (Cox & Mann, 2008; Cox et al, 2011; Tyanova et al, 2016)	
SAINTexpress	Described in (Teo et al, 2014)	
Cytoscape	Described in (Shannon et al, 2003)	
stringApp	Described in (Doncheva et al, 2019)	
**Other**		
96-well µ-plate square wells	Ibidi	#89621
18-well µ-slide glass bottom	Ibidi	#81817
Duolink In Situ Red Starter Kit Mouse/Rabbit	Sigma-Aldrich	#DUO92101-1KT
DC Protein Assay Kit II	Bio-Rad	#5000112
4–20% Mini-PROTEAN® TGX Stain-Free™ Protein Gels, 15 well, 15 µl	Bio-Rad	#4568096
4–20% Mini-PROTEAN® TGX Stain-Free™ Protein Gels, 10 well, 30 µl	Bio-Rad	#4568093
Trans-Blot Turbo Mini 0.2 µm PVDF Transfer Packs	Bio-Rad	#1704156
Trans-Blot Turbo Transfer System	Bio-Rad	
Clarity Western ECL substrate, 500 ml	Bio-Rad	#1705061
ChemiDoc imager	Bio-Rad	
40x (0.95 NA) oil Plan Apo Lambda DM objective	Nikon	
100x (1.45 NA) oil Plan Apo Lambda DM objective	Nikon	
Spinning Disk confocal microscope	Nikon	
63x (1.46 NA) oil alpha Plan Apo objective	ZEISS	
Elyra 7 Lattice SIM microscope	ZEISS	
100x (1.4 NA) oil objective	Olympus	
STED expert line microscope	Abberior Instruments	
EM Critical Point Dryer (CPD300)	Leica	
Leit Adhesive Carbon Tabs 12 mm	Oxford Instruments	#AGG3347N
SEM Specimen Stubs for JEOL instruments. 12.5 mm dia, 10 mm high.	Oxford Instruments	#AGG3384
SEM IT700HR	JEOL	
35 mm Glass-bottom dishes	MatTek	#P35G-1.5-14-CGRD
40x (1.3 NA) oil EC plan-neofluar objective	ZEISS	
Axiovert 200 M light microscope	ZEISS	
Field emission scanning electron microscope (FESEM) Zeiss Auriga microscope equipped with a CrossBeam workstation	ZEISS	
QUANTIFOIL® R2/2 on AuH2 finder grids	Quantifoil	#N1-C16nAuH2-01
µ-Dish 35 mm, high glass bottom,	Ibidi	#81158
ELMO glow discharge system	Cordouan	
EM Protein-A/Gold, 10 nm	EMS	#25284
EMGP system	Leica	
Cryo-FIB Autogrids	Thermo Fisher Scientific	#1205101
Aquilos 2 dual-beam cryo-focused ion beam scanning electron microscope (cryo-FIB-SEM) instrument	Thermo Fisher Scientific	
300 kV Titan Krios G3	Thermo Fisher Scientific	
GFP-Trap Agarose Kit	Chromotek	#gtak-20
S-Trap micro column	Protifi	#C002‑MICRO‑0010PK
AssayMAP C18 cartridges on the AssayMAP Bravo platform	Agilent	
Q ExactiveTM Plus Mass Spectrometer	Thermo Fisher Scientific	

### Cell culture

Primary Human Umbilical Vein Endothelial Cells (HUVECs, pooled donors, Lonza, #C2519A) were cultured in complete Endothelial Cell Growth Medium (EGM)-2 (EBM-2 supplemented with EGM-2 SingleQuots Supplements Lonza, #CC-3162). hTERT-immortalized HUVECs stably expressing mCherry-FTractin or not (hTERT-HUVECs, both described in (Hayer et al, 2016) and kindly provided by Arnold Hayer, McGill University, Montreal) were cultured in complete EGM-2 medium supplemented with 50 µg/ml of Hygromycin B (Euromedex, #EU3250). Human Nasal Epithelial Cells (HNEpCs, PromoCell, #C-12620) were cultured in complete Airway Epithelial cell Growth Medium (AEGM) (Basal Medium supplemented with SupplementMix, PromoCell, #C-21060). For all *N. meningitidis* infection experiments, cells were cultured in antibiotic-free medium.

### Cell transfection and plasmids

In all, 0.5 × 10^6^ cells were electroporated in 100 µl of Ingenio Electroporation solution (MIRUS Bio, #MIR50114) with 2 µg of plasmid DNA using AMAXA Nucleofector device (U-001 program, Amaxa Biosystem, Lonza). Cells were then resuspended in 1.4 ml of antibiotic-free EGM-2 medium, seeded on collagen-coated coverslips in wells of 24-well plates (3 × 500 µl) and incubated overnight at 37 °C and 5% CO_2_ in a moist atmosphere prior to being infected. The following plasmids were used: PM-EGFP (palmitoylation and myristoylation signals from the Lyn protein, described in (Bag et al, 2021) and kindly provided by Barbara Baird and David Holowka, Cornell University, Ithaca), pEGFP-C3 (described in (Pyenta et al, 2001) and kindly provided by Sandrine Etienne-Manneville, Institut Pasteur, Paris), ArpC5-GFP (kindly provided by Phillipe Chavrier, Institut Curie, Paris), pcDNA3-EGFP-Rac1(WT) (Addgene, #13719), and pCDNA3-EGFP-Rac1(T17N) (Addgene, #13721), both described in (Kraynov et al, 2000); pcDNA3-EGFP-Cdc42(wt) (Addgene, #12599) and pcDNA3-EGFP-Cdc42(T17N) (Addgene, #12601), both described in (Nalbant et al, 2004); mRFP-wGBD (Addgene, #26733), described in (Benink and Bement, 2005), and pMAG201-EGFP-Arf1 (kindly provided by Cathy Jackson, Institut Jacques Monod, Paris); pcDNA3 HA-Arf1 (Addgene, #10830) and pcDNA3 HA-Arf1-ActQ71L (Addgene, #10832), both described in (Furman et al, 2002).

### siRNA-mediated protein silencing

The day before transfection, 0.5 × 10^6^ cells were seeded in petri dishes (100 mm × 20 mm, TPP, #193100) in antibiotic-free EGM-2 medium. The day after, cells were transfected with ON-TARGETplus Non-Targeting pool (NT, #D-001810-10-05), ON-TARGETplus Human SMARTpool Arp2 (ACTR2, #L-012076-02-0005), Arp3 (ACTR3, #L-012077-00-0005), N-WASP (WASL, #L-006444-00-0005), Arf1 (#L-011580-00-0005), or CD147 (BSG, #L-010737-00-0005) siRNAs (all purchased from Horizon Discovery) diluted in 2 ml of OptiMEM I Reduced Serum Medium (Thermo Fisher Scientific, #10149832) to a final quantity of 200 pmol (1 nmol for N-WASP-targeting SMARTPool siRNA) and using 25 µl of Lipofectamine RNAiMAX (Thermo Fisher Scientific, #13778075). Cells were incubated with the transfection mix for 2 days at 37 °C and 5% CO_2_ in a moist atmosphere prior to being used. Silencing efficiency was analyzed by Western blot.

### Chemical inhibitor treatments

The day before infection, 0.5 × 10^5^ cells were seeded on collagen-coated coverslips in wells of a 24-well plate and incubated overnight at 37 °C and 5% CO_2_ in a moist atmosphere. The day after, medium was removed and cells were pre-treated with either CK689 or CK666 (100 µM each, Sigma-Aldrich, #182517 and Tocris, #3950, respectively), CK312 or CK869 (20 µM each, Sigma-Aldrich, #182518 and #182516, respectively), DMSO (Sigma-Aldrich), SMIFH2 (1 µM, Tocris, #4401) or Brefeldin A (30 µM, Sigma-Aldrich, #B6542), and H_2_O or Isoproterenol (10 µM, Sigma-Aldrich, #PHR2722) diluted in cell culture medium for 30 min at 37 °C and 5% CO_2_ in a moist atmosphere, prior to be infected in presence of the corresponding drug.

### Bacterial strains and growth conditions

*Neisseria meningitidis* strain 8013 clone 12 used in this study is a serogroup C clinical isolate (Nassif et al, 1993; Rusniok et al, 2009) expressing either the blue (BFP), green (GFP), or near-infrared (iRFP) fluorescent protein, as previously described (Charles-Orszag et al, 2018). Bacterial frozen stock was grown overnight on GCB-Agar plates containing Kellogg’s supplements (Difco) and selection antibiotics when required (chloramphenicol, 5 µg/ml) at 37 °C and 5% CO_2_ in a moist atmosphere. For infection experiments, liquid precultures were started from GCB-Agar plates by adjusting bacterial OD_600nm_ to 0.05 in antibiotic-free cell culture medium. Precultures were incubated for 2 h at 37 °C, 5% CO_2_ and constant agitation (140 rpm) before being used for cell infection.

### Bacterial growth curves

Non-fluorescent bacteria grown overnight on GCB-agar plates were adjusted to an OD_600nm_ of 0.05 and incubated for 2 h at 37 °C and gentle agitation (140 rpm) in antibiotic-free cell culture medium. The bacterial precultures were then diluted to an OD_600nm_ of 0.005 and transferred into wells of a 96-well plate in the presence or not of either CK689 (100 µM), CK666 (100 µM), CK312 (20 µM), CK869 (20 µM), DMSO, or SMIFH2 (1 µM). Each condition was performed in triplicate. The bacteria-containing 96-well plate was incubated in a Cytation5 apparatus (BioTek) at 37 °C and 5% CO_2_ in a moist atmosphere. The OD_600nm_ was measured every 30 min for 18 h.

### Bacterial aggregation assay

GFP-expressing bacteria grown overnight on GCB-agar plates were adjusted to an OD_600nm_ of 0.05 and incubated for 2 h at 37 °C in RPMI medium supplemented with 10% FBS with constant agitation (140 rpm). The bacterial preculture was then concentrated to an OD_600nm_ of 0.3 by centrifugation at 775×*g* for 5 min, followed by resuspension in RPMI medium. Bacterial suspensions were briefly vortexed, transferred into wells of a 96-well µ-plate with square wells (Ibidi, #89621) and allowed to form aggregates for a period of 30 min at 37 °C and 5% CO_2_ in a moist atmosphere in presence of either CK689 (100 µM), CK666 (100 µM), CK312 (20 µM), CK869 (20 µM), DMSO, or SMIFH2 (1 µM). Four fluorescence images per well were captured using a 20x Plan Apo objective (NA 0.75, Nikon). The surface covered by bacterial aggregates was quantified in each field of view using a homemade macro in Fiji (Kennouche et al, 2019).

### Cell infection

Protocol optimizations were made to achieve a similar level of infection in both cell types, allowing to get ~1 bacterial colony per cell, with sizes of bacterial colonies within the same range, while minimizing cell death. No apparent sign of cell death (excessive blebbing, nuclear fragmentation, loss of membrane dynamics or organelle movement by live-cell imaging) was observed in our experimental conditions.

For endothelial cells, bacterial precultures were diluted in antibiotic-free EGM-2 medium to reach a MOI (Multiplicity Of Infection) of 100, added to the cells and incubated for 30 min at 37 °C and 5% CO_2_ in a moist atmosphere to allow bacterial adhesion. Endothelial cells were extensively washed with antibiotic-free EGM-2 medium to remove non-adherent bacteria and incubated for another 2 h period in fresh antibiotic-free EGM-2 medium at 37 °C and 5% CO_2_ in a moist atmosphere to allow bacterial proliferation and the formation of microcolonies. In conditions in which the function of meningococcal type IV pili was blocked, cells were treated with 0, 13, or 26 µg/ml of anti-PilE 20D9 antibody (Charles-Orszag et al, 2018) during both the bacterial adhesion phase (30 min) and the bacterial proliferation and microcolony formation phase (2 h).

For epithelial cells, bacterial precultures were diluted in antibiotic-free AEGM medium to reach a MOI of 200, added to the cells and incubated for 1 h at 37 °C and 5% CO_2_ in a moist atmosphere to allow bacterial adhesion. Epithelial cells were then extensively washed with antibiotic-free AEGM medium to remove non-adherent bacteria and incubated for another 3 h period in fresh antibiotic-free AEGM medium at 37 °C and 5% CO_2_ in a moist atmosphere to allow bacterial proliferation and the formation of microcolonies.

After infection, both endothelial and epithelial cells were washed once with 1X PBS, fixed 15 min at room temperature with 4% PFA diluted in 1X PBS, and extensively washed with 1X PBS prior to subsequent experiments.

### Live-cell imaging of infected cells with preformed bacterial aggregates

The day before infection, 0.5 × 10^6^ cells were electroporated with ArpC5-GFP or with both pcDNA3-EGFP-Cdc42(wt) and mRFP-wGBD as described above prior to being seeded in collagen-coated 18-well glass bottom µ-slide (Ibidi, #81817) and incubated overnight at 37 °C and 5% CO_2_ in a moist atmosphere. The day after, liquid precultures of iRFP-expressing *N*. *meningitidis* were started from GCB-Agar plates by adjusting bacterial OD_600nm_ to 0.05 in antibiotic-free EGM-2 medium. Precultures were incubated for 3 h at 37 °C, 5% CO_2_ and constant agitation (140 rpm) before being used for cell infection to allow the formation of bacterial aggregates. After 3 h, the µ-slides were set under the microscope at 37 °C and 5% CO_2_ prior to being infected using the liquid precultures (no vortex step was applied to avoid breaking bacterial aggregates). *N. meningitidis*-infected cells were imaged using a 100x (1.45 NA) oil Plan Apo Lambda DM objectives (Nikon) fitted on a Spinning Disk confocal microscope (Nikon) equipped with a 95B Prime camera (Photometrics). Multi-channel z-stack (0.2 µm z-step) time-lapse acquisitions (1 image/6 s during 2 min) were performed using MetaMorph software (version 7.10.4.407, Molecular Device).

### Immunofluorescence staining

Cells were permeabilized with PBS-Triton (0.1% Triton X-100 diluted in 1X PBS) for 10 min at room temperature, extensively washed with 1X PBS, blocked using PBS-Gelatin (0.2% fish gelatin diluted in 1X PBS) for 20 min at room temperature and then incubated with primary antibodies diluted in PBS-Gelatin for 1 h at room temperature. Cells were then extensively washed with 1X PBS and incubated with secondary antibodies diluted in PBS-Gelatin for 30 min at room temperature and protected from light. Samples were extensively washed with 1X PBS, mounted using Fluoromont-G (Southern Biotech) or Mowiol (Sigma-Aldrich) and stored at 4 °C protected from light until ready to proceed with image acquisition.

Protein–protein interaction between Arf1 and Cdc42 was assessed on endothelial cells transiently co-expressing Arf1-WT-HA and Cdc42-WT-GFP using the Proximity Ligation Assay (PLA) Duolink In Situ Red Starter Kit Mouse/Rabbit (Sigma-Aldrich, #DUO92101-1KT) according to the manufacturer’s instructions. The following primary antibodies were used: Rabbit anti-GFP (1/20, Thermo Fisher Scientific, #50430-2-AP) and Mouse anti-HA.11 Epitope Tag (1/500, BioLegend, #901501).

### Western blotting

Cells were lysed at 4 °C in RIPA buffer supplemented with 1X cOmplete EDTA-free Protease Inhibitor Cocktail Tablets (Sigma-Aldrich, #11873580001). Total protein concentrations in whole cell lysates were determined using the DC Protein Assay Kit II (#5000112) according to the manufacturer’s instructions. Proteins were then loaded onto 4–20% gradient mini-Protean TGX Stain-Free gels (Bio-Rad, #4568096 and #4586093 for 15- and 10-well gels, respectively) and transferred onto polyvinylidene fluoride membranes (Trans-Blot Turbo Mini 0.2 µm PVDF Transfer Packs, Bio-Rad, #1704156) using the Trans-Blot Turbo Transfer System (Bio-Rad). Membranes were blocked using 5% non-fat dry milk diluted in 1X TBS-0.05% Tween 20 and incubated overnight at 4 °C with primary antibodies, followed by 1 h incubation with secondary antibodies. Western blots were developed using Clarity Western ECL substrate (Bio-Rad, #1705061), and chemiluminescence was detected using the ChemiDoc imager.

### Antibodies

For immunofluorescence, the following primary antibodies were used: anti-CD31/PECAM-1 (1/200, mouse monoclonal, clone WM59, BioLegend, #303102), anti-CD326/EpCAM (1/1000, rabbit monoclonal, clone EPR20532-225, Abcam, #ab223582), anti-Arp2 (1/500, rabbit polyclonal, Abcam, #ab47654), anti-Arp3 (1/500, mouse monoclonal, clone FMS338, Sigma-Aldrich, #A5979), anti-N-WASP (1/300, rabbit monoclonal, clone EPR6959, Abcam, #ab126626), anti-WAVE1 (1/500, rabbit polyclonal, Abcam, #ab272916), anti-Moesin (1/1000, rabbit monoclonal, clone EP1863Y, Abcam, #ab52490), anti-Spectrin β IV (1/100, mouse monoclonal, clone S393-2, Thermo Fisher Scientific, #MA5-27608), anti-α-actinin-1 (1/100, rabbit polyclonal, Proteintech, #11313-2- AP), anti-α-actinin-4 (1/50, rabbit polyclonal, Proteintech, #19096-1-AP), anti-c-Abl (1/100, mouse monoclonal, clone 8E9, Thermo Fisher Scientific, #MA5-14398), anti-CSNK2A1 (1/200, rabbit polyclonal, Proteintech, #10992-1-AP), anti-PDLIM1/CLP36 (1/200, rabbit polyclonal, Proteintech, #11674-1-AP), anti-GM130 (1/500, mouse monoclonal, BD Transduction Laboratories, #610822), anti-GFP (rabbit polyclonal, Abcam, #ab290, 1/500 or Thermo Fisher Scientific, #A50430-2-AP, 1/20), and anti-HA.11 Epitope Tag (1/1000, mouse monoclonal, clone 16B12, BioLegend, #901501). The following secondary antibodies were used: AlexaFluor488- or AlexaFluor568-conjugated goat anti-rabbit (1/200, Thermo Fisher Scientific, #A11008 and #A11011, respectively), AlexaFluor488- or AlexaFluor568-conjugated goat anti-mouse (1/200, Thermo Fisher Scientific, #A21121 and #A11004, respectively), and STAR RED anti-rabbit (1/200, goat polyclonal, Abberior, #STRED-1002-500UG). F-actin was stained using AlexaFluor405-, AlexaFluor488-, AlexaFluor568-, or AlexaFluor647-conjugated phalloidin (1/1000, Thermo Fisher Scientific, #A30104, #A12379, #A12380, and #A22287, respectively).

For Western blotting, the following antibodies were used: anti-Arp2 (1/500, rabbit polyclonal, Abcam, #ab47654), anti-Arp3 (1/200, mouse monoclonal, clone FMS338, Sigma-Aldrich, #A5979), anti-Cdc42 (rabbit monoclonal, clone EPR15620, Abcam, #ab187643 or rabbit polyclonal, Cell Signaling, #2462, both at 1/1000), anti-N-WASP (1/1000, rabbit monoclonal, clone EPR6959, Abcam, #ab126626), anti-ARF1 (1/1000, rabbit polyclonal, Proteintech, #20226-1-AP), anti-CD147 (1/5000, rabbit polyclonal, Proteintech, #11989-1-AP), anti-Vinculin (1/1000, mouse monoclonal, clone hVIN-1, Sigma-Aldrich, #V9264), and anti-GFP (1/5000, chicken polyclonal, Abcam, #ab13970). The following secondary antibodies were used: HRP-conjugated goat anti-rabbit, HRP-conjugated goat anti-mouse and HRP-conjugated donkey anti-chicken (all at 1/5000, Jackson ImmunoResearch, #111-035-144, #115-035-146, and #703-035-155, respectively).

### Acquisition of fluorescence images

Immunofluorescence stainings were imaged using 40x (0.95 NA) or 100x (1.45 NA) oil Plan Apo Lambda DM objectives (Nikon) fitted on a Spinning Disk confocal microscope (Nikon) equipped with a 95B Prime camera (Photometrics). Multi-channel z-stack (0.5 or 0.2 µm z-step) images were acquired using MetaMorph software (version 7.10.4.407, Molecular Devices).

The super-resolved structured illumination microscopy (SIM) images were acquired using a 63x (1.46 NA) oil alpha Plan Apo objective with a 1.518 refractive index oil (ZEISS) fitted on an Elyra 7 Lattice SIM microscope (ZEISS) equipped with two sCMOS PCO Edge 4.2 camera for the detection. The alignment of the two cameras was assessed and corrected using fluorescent beads (Tetraspeck, Thermo Fisher Scientific). Thirteen images per plane and per channel were acquired with a spacing of 0.116 μm in z to perform the reconstruction of a 3D-SIM image using ZEN black software (ZEISS).

The super-resolved STimulated emission depletion (STED) images were acquired using a 100x (1.4 NA) oil objective (Olympus) fitted on a STED expert line microscope (Abberior Instruments) equipped with Avalanche Photo-Diodes (APDs, single point photon counting modules) detector (Excelitas). The wavelength of excitation was 640 nm for STAR RED, and the laser line for STED was 775 nm, pulsed. Pixel size of acquired images was 15 nm, and an elementary pixel dwell time of 1 μs was used with up to a few tens of line averages, depending on the acquisitions.

### Analysis and quantification of fluorescence images

The analysis pipeline detailed in Fig. EV1 was developed using the open-source software Fiji (ImageJ) (Schindelin et al, 2012). Following z-stack acquisition, images were reoriented along the xz-axis using the *Reslice* function. Based on the bacterial fluorescence, an average y-projection of the slices containing the bacterial microcolony was generated. A line was manually drawn along the cell apical surface using the channel of the plasma membrane signal. The fluorescence intensity profile of each channel was then measured along this line (raw fluorescence intensity profiles). Data per cell were then binned with a step of 1 µm and normalized using the median value of the fluorescence intensity profile. Normalized fluorescence profiles were then centered with respect to the center of each bacterial microcolony, allowing to average the profiles of many cells. This process was semi-automatized using the open community platform Icy (de Chaumont et al, 2012). Finally, the mean value per cell of the normalized fluorescence intensities in the non-infected and infected areas were calculated by averaging the binned fluorescence intensity values extracted from the respective areas. The size of each bacterial microcolony was manually determined by measuring the width of the bacterial aggregate on the average projection. Single-cell images shown in the figures were cropped from large fields, rotated, and their contrast and brightness manually adjusted.

### Scanning electron microscopy (SEM)

About 0.5 × 10^5^ cells were seeded on collagen-coated coverslips in antibiotic-free culture medium and incubated overnight at 37 °C and 5% CO_2_ in a moist atmosphere. The day after, cells were infected as described above. Cells were then gently washed once with 1X PHEM (60 mM PIPES, 25 mM HEPES, 10 mM EGTA, 2 mM MgCl_2_, and pH 7.3) and fixed with 4% PFA + 0.1% glutaraldehyde diluted in 1X PHEM for 1 h at room temperature. Samples were then washed once with 1X PHEM, postfixed in 1% osmium tetroxide (EMS, #19152) for 1 h, washed with water, and dehydrated in an ethanol (Sigma-Aldrich) series of 25, 50, 75, 95, and 100%. Samples were desiccated using a Critical Point Dryer (CPD300, Leica), mounted on SEM stubs (Oxford Instruments, #AGG3384), and sputtered with 20 nm gold/palladium. Infected cells were observed using a SEM IT700HR (JEOL) at 5 kV with a secondary electron detector.

### Focused ion beam scanning electron microscopy (FIB-SEM)

#### Sample preparation

About 0.9 × 10^5^ cells were seeded on 35 mm glass-bottom dishes with alphanumeric coordinates (MatTek, #P35G-1.5-14-CGRD) in antibiotic-free culture medium and incubated overnight at 37 °C and 5% CO_2_ in a moist atmosphere. The day after, cells were infected as described above. Twenty minutes before the end of the infection, the plasma membrane of endothelial and epithelial cells was respectively stained using DyLight649-labeled Ulex Europaeus Agglutinin I lectin (UEA-1, 1/200, Vector Laboratories, #DL-1068) or BioTracker655 Red Cytoplasmic Membrane Dye (1/200, Sigma-Aldrich, #SCT108). After labeling, cells were washed once with 1X PHEM and fixed 1 h at room temperature with 4% PFA + 0.1% glutaraldehyde diluted in 1X PHEM. An inverted widefield microscope was then used to map the fluorescently labeled cells in each dish and determine the alphanumeric coordinates of the cells of interest (infected by GFP-expressing bacteria). Maps were acquired using a 40x (1.3 NA) oil EC Plan-Neofluar objective (ZEISS) fitted on an Axiovert 200 M Light microscope (ZEISS) equipped with an ORCA-Flash4.0 LT3 Digital CMOS camera (Hamamatsu Photonics). Multi-channel z-stacks (0.5 z-step) of large tile regions (round area of 12 × 12) were acquired and processed/stitched using ZEN blue (version 2.6) software (ZEISS). Maps were used to retrieve the cells of interest under the FIB-SEM microscope. Following cell post-fixation with 1% osmium (EMS) and 1.5% potassium ferrocyanide (Sigma-Aldrich) diluted in 1X PHEM buffer for 1 h, samples were treated for 30 min with 1% tannic acid (Sigma-Aldrich) and 1 h with 1% osmium tetroxide (EMS), rinsed with water, and incubated in 1% uranyl acetate dissolved in 25% ethanol for 30 min. Cells were then dehydrated in an ethanol (Sigma-Aldrich) series of 25, 50, 75, and 95% (5 min each), and three times in 100% (10 min each). Cells were embedded in epoxy resin (EMS) after 48 h at 60 °C of polymerization. Embedded samples were mounted on aluminum stubs.

#### Tomographic datasets acquisition

Tomographic datasets were obtained using a field emission scanning electron microscope (FESEM) Zeiss Auriga microscope equipped with a CrossBeam workstation and acquired using ATLAS 3D software (all from ZEISS). The specimen stage was tilted at 54° with 5 mm working distance of the pole piece, at the coincidence point of the electron and the galion beams. The milling conditions for the trench that allowed the view of the cross-section were 20 nA at an accelerating voltage of 30 kV. The fine polishing of the surface block was performed with 10 nA at 30 kV. For the slice series, the conditions were as follows: 0.5 nA milling current of the Gallium emitter, leading to the removal of 10 nm at a time from the epoxy resin. Scanning EM images were recorded with an aperture of 60 µm in the high-current mode at 2 kV of the in-lens EsB detector with the EsB grid set to −1000 V. Voxel size was 10 nm in x/y and 10 nm in z. Alignment of image stacks was done with the open-source software Fiji (ImageJ).

#### Image processing

The Amira software (Amira 3D from version 2020.2 to 2022.1; Thermo Fisher Scientific) was used for segmentation, 3D reconstruction and volume visualization. Contrast of the images was inverted to conventional brightfield.

### Cryo-electron tomography (Cryo-ET)

#### Sample preparation

Carbon-coated gold grids (AuH2 finder, R2/2, Quantifoil, #N1-C16nAuH2-01) were placed into µ-Dish (35 mm, high glass bottom, Ibidi, #81158) and glow-discharged at 2 mA and 1.4–1.7 × 10^−1 ^mbar for 1 min in an ELMO glow discharge system (Cordouan). Then, 0.9 × 10^5^ cells were seeded on these grids in cell culture medium and incubated overnight at 37 °C and 5% CO_2_ in a moist atmosphere. The day after, cells were infected as described above and their plasma membrane labeled using DyLight649-labeled UEA-1 lectin (endothelial cells) or BioTracker655 Red Cytoplasmic Membrane Dye (epithelial cells), as described above. After labeling, cells were washed once with 1X PHEM and fixed 1 h at room temperature with 4% PFA + 0.1% glutaraldehyde diluted in 1X PHEM. Grids were then mapped using an inverted widefield microscope to locate the cells of interest (infected with GFP-expressing bacteria) on the alphanumeric coordinates of the grids. Maps were acquired using a 40x (1.3 NA) oil EC Plan-Neofluar objective (ZEISS) fitted on an Axiovert 200 M Light microscope (ZEISS) equipped with an ORCA-Flash4.0 LT3 Digital CMOS camera (Hamamatsu Photonics). Multi-channel z-stacks (0.5 z-step) of large tile regions (round area of 10 per 10) were acquired and processed/stitched using ZEN blue (version 2.6) software (ZEISS). For cell vitrification, 4 µl of Protein-A/Gold (10 nm gold fiducials, EMS, #25284) was added on top of fluorescently labeled cells, blotted from the back side of the grid for 9 s, then rapidly frozen in liquid ethane using an EMGP system (Leica) and clipped into cryo-FIB Autogrids (Thermo Fisher Scientific, #1205101). The grids were stored in liquid nitrogen until cryo-lamellae preparation and image acquisition.

#### Cryo-lamellae preparation

Grids were coated with a thin layer of organometallic platinum (~70 nm) after a map of the grid was acquired at 5 kV and 25 pA with the electron beam, prior to performing the milling. Fluorescent maps imported from the Axiovert 200 M Light microscope were used to correlate positions and target *Nm*-infected cells, through MAPS 3.17 software (Thermo Fisher Scientific). Cryo-lamellae were prepared on an Aquilos 2 dual-beam cryo-focused ion beam scanning electron microscope (cryo-FIB-SEM; Thermo Fisher Scientific) instrument equipped with a cryo-transfer system, a cryo-stage, where a Gallium ion beam, accelerated at 30 kV, has been milling the samples at a working distance of 7 mm on the vitrified grids loaded onto a 45° pretilted sample holder. Lamellae were premilled to 1 µm thickness in automated mode using Thermo Fisher AutoTEM 2.2.0 software overnight, and then manually thinned down to 100–200 nm, the day after. Ion beam current was modulated from 1 nA to 30 pA to thin down lamellae while using a 2 kV 25 pA Electron beam to monitor the milling process.

#### Tilt series acquisition

Tilt series were first collected on a 300 kV Titan Krios G3 (Thermo Fisher Scientific) transmission electron microscope, equipped with a Gatan BioQuantum LS Imaging Filter with slit width set to 20 eV, and a Gatan K3 direct electron detector. During these sessions, tomograms were acquired using a dose symmetric scheme, with an angular range of ±60°, increment of 2° and 3° tilt steps, defocus range from −3 to −8 µm, pixel size from 2.64 to 3.39 Å (26000x to 33000x), dose rate of 20e/pix/sec, while total electron dose varied from 120 to 150 e/Å² for optimization. Starting July 2022, the Titan Krios microscope was upgraded with a Cold Feg tip, a Selectris X energy filter, and a Falcon 4i direct electron detector (all from Thermo Fisher Scientific). Tilt series were then collected with the same dose symmetric scheme and angular range of ±60°, tilt increments of 2° and 3°, pixel size of 1.9 Å (64000x) and 3.1 Å (42000x), dose rate of 11-14e/pix/s, while total electron dose varied from 140 e/Å² to 200 e/Å². Tilt series were acquired with SerialEM software (from version 3.7 to 4.1, David Mastronade, University of Colorado, Boulder, RRID:SCR_017293). Dose symmetric tilt series were saved as separate stacks of frames, motion-corrected and restacked using the alignframes module in SerialEM.

#### Tomogram reconstruction and segmentation

Three-dimensional reconstructions were calculated in IMOD 4.9.10 software (David Mastronade, University of Colorado, Boulder, RRID:SCR_003297) with dose weighting and CTF correction by weighted back projection and SIRT-like filter with 15 iterations. An additional Gaussian filter was used to enhance contrast and facilitate subsequent segmentation analysis. The Amira software (Amira 3D from version 2020.2 to 2022.1; Thermo Fisher Scientific) was used for segmentation, 3D reconstructions and volume visualization. Following manual segmentation and 3D reconstruction, the *Angle* tool was used to manually draw a line along each of the two actin filaments forming the angle to measure. Angles were only measured on actin filaments that were on the same focal plane and that were fully connected to each other to ensure accurate measurements of actual actin branches.

### Affinity purification–mass spectrometry analysis of protein interactions

#### Samples preparation

The day before infection, 8x[0.5 × 10^6^] cells were electroporated with either EGFP-C3, ArpC5-GFP, or Cdc42-WT-GFP, as described above. For each condition, cells were pooled, seeded in four wells of a six-well plate in antibiotic-free EGM-2 medium and incubated overnight at 37 °C and 5% CO_2_ in a moist atmosphere. The day after, cells were infected as described above.

#### GFP-Trap immunoprecipitation

After infection, cells were washed once with 1X PBS, incubated in Trypsin/EDTA until detached, resuspended in antibiotic-free EGM-2 medium, and then centrifuged 5 min at 400×*g*. Supernatants were removed, and cell pellets were resuspended in 200 µl of ice-cold Lysis buffer (GFP-Trap Agarose Kit, Chromotek, #gtak-20) supplemented with cOmplete EDTA-free Protease Inhibitor Cocktail (Sigma-Aldrich) for 30 min on ice and extensively vortexed every 10 min. Cell lysates were then centrifuged at 17.000×*g* for 10 min at 4 °C on a benchtop microcentrifuge. The co-immunoprecipitation of GFP fusion proteins was then performed using the GFP-Trap Agarose Kit (ChromoTek) according to the manufacturer’s instructions.

#### Input digestion

Input samples (whole cell lysates) were processed with the S-Trap^TM^ micro column (Protifi, Huntington, USA) according to the manufacturer’s instructions. Briefly, SDS was added to a final concentration of 5%, then proteins were reduced with 5 mM TCEP for 15 min at 55 °C and alkylated with 500 mM MMTS for 10 min at room temperature. Phosphoric acid was then added to a final concentration of 2.5%, followed by the addition of binding/wash buffer (100 mM TEAB in 90% methanol). Mixtures were then loaded on S-Trap columns and washed four times for thorough SDS elimination. At the end, proteins were digested with 1 μg of Sequencing Grade Modified Trypsin (Promega) overnight at 37 °C. After elution, peptides were concentrated to dryness and resuspended in 2% acetonitrile (ACN)/0.1% formic acid (FA) just prior to LC-MS injection.

#### On-bead protein digestion

GFP-capture beads with the protein complexes were resuspended in 8 M urea, 100 mM Tris HCl, pH 8.5. Samples were reduced using 5 mM TCEP for 30 min at room temperature. Alkylation of the reduced disulfide bridges was performed using 10 mM iodoacetamide for 30 min at room temperature in the dark. Then, samples were diluted below 2 M urea with 100 mM Tris HCl, pH 8.5, and 500 ng Sequencing Grade Modified Trypsin (Promega) was added for the digestion overnight at 37 °C. Proteolysis was stopped by adding formic acid (FA) at a final concentration of 5%. The resulting peptides were cleaned using AssayMAP C18 cartridges on the AssayMAP Bravo platform (Agilent) according to the manufacturer’s instructions. Peptides were concentrated to dryness and resuspended in 2% acetonitrile (ACN)/0.1% FA just prior to LC-MS injection.

#### LC-MS/MS analysis

LC-MS/MS analysis was performed on a Q Exactive^TM^ Plus Mass Spectrometer (Thermo Fisher Scientific) coupled with a Proxeon EASY-nLC 1200 (Thermo Fisher Scientific). Half of the sample was injected onto a homemade 31 cm C18 column (1.9-μm particles, 100 Å pore size, ReproSil-Pur Basic C18, Dr. Maisch GmbH, Ammerbuch-Entringen, Germany). Column equilibration and peptide loading were done at 900 bars in buffer A (0.1% FA). Peptides were separated with a multi-step gradient from 3 to 6% buffer B (80% ACN and 0.1% FA) in 5 min, 6 to 31% buffer B in 80 min, 31 to 62% buffer B in 20 min at a flow rate of 250 nL/min. Column temperature was set to 60 °C. MS data were acquired using Xcalibur software (Thermo Fisher Scientific) using a data-dependent method. MS scans were acquired at a resolution of 70,000, and MS/MS scans (fixed first mass 100 m/z) at a resolution of 17,500. The AGC target and maximum injection time for the survey scans and the MS/MS scans were set to 3E6, 20 ms and 1E6, 60 ms, respectively. An automatic selection of the ten most intense precursor ions was activated (Top 10) with a 30 s dynamic exclusion. The isolation window was set to 1.6 m/z and the normalized collision energy was fixed to 27 for HCD fragmentation. We used an underfill ratio of 1.0% corresponding to an intensity threshold of 1.7E5. Unassigned precursor ion charge states as well as 1, 7, 8, and >8 charged states were rejected and peptide match was disabled.

#### Protein identification and quantification

Acquired Raw data were analyzed using MaxQuant software version 2.1.1.0 using the Andromeda search engine (Cox and Mann, 2008; Cox et al, 2011; Tyanova et al, 2016). The MS/MS spectra were searched against the *Nm* 8013 (1894 entries) and the *Homo sapiens* (20,391 entries) databases. All searches were performed with oxidation of methionine and protein N-terminal acetylation as variable modifications and cysteine carbamidomethylation as a fixed modification. Trypsin was selected as a protease allowing for up to two missed cleavages. The minimum peptide length was set to five amino acids and the peptide mass was limited to a maximum of 8000 Da. The false discovery rate (FDR) for peptide and protein identification was set to 0.01. The main search peptide tolerance was set to 4.5 ppm and to 20 ppm for the MS/MS match tolerance. The second peptides were enabled to identify co-fragmentation events. A false discovery rate cut-off of 1% was applied at the peptide and protein levels.

#### Pairwise differential analyses

To find the proteins more abundant in one condition than in another, the intensities quantified using MaxQuant were compared. Reverse protein hits, potential contaminants, and proteins not well identified with a 1% FDR (“Only identified by site”) were first removed from the analysis. Only proteins identified with at least one peptide that is not common to other proteins in the FASTA file used for the identification (at least one “unique” peptide) were kept. Additionally, only proteins with at least two quantified intensity values in one of the two compared conditions were kept for further statistics. After this filtering, intensities of the remaining proteins were first log-transformed (log2). Next, intensity values were normalized by median centering within each condition (section 3.5 in Giai Gianetto (2023). Proteins not quantified in a condition and quantified in another were put aside and have been assumed to be differentially abundant between the conditions. Missing values of the remaining proteins were imputed using the impute.slsa function of the R package imp4p (Giai Gianetto et al, 2020). Statistical testing was conducted using a limma *t*-test thanks to the R package limma (Ritchie et al, 2015). An adaptive Benjamini–Hochberg procedure was applied on the resulting *p* values thanks to the function adjust.p of the cp4p R package (Giai Gianetto et al, 2016) using the robust method described in (Pounds and Cheng, 2006) to estimate the proportion of true null hypotheses among the set of statistical tests. The proteins associated with an adjusted *p* value inferior to an FDR of 1% and with a log2(fold change) threshold superior to 1 have been considered as significantly differentially abundant proteins. Finally, the proteins of interest are therefore those that emerge from this statistical analysis, supplemented by those that are not quantified in one condition and quantified in another.

#### Scoring of protein–protein interactions

SAINTexpress (Teo et al, 2014) was used to score protein–protein interactions with bait proteins (AprC5 or Cdc42). The corresponding non-infected and infected samples of non-transfected and cytoplasmic GFP-expressing cells were used as controls. Posterior probabilities of true interactions (“AvgP”) superior to 10% means that the hypothesis of an interaction cannot be rejected with a 10% threshold: it can potentially be true. We combined the scores of infected and non-infected conditions to estimate two probabilities. The first is the probability of an interaction when there is infection and no interaction when there is no infection (CS1 = (AvgP in Inf)*(1-Avg in No inf)). The second is the probability of an interaction when there is no infection and no interaction when there is infection (CS2 = (AvgP in No Inf)*(1-Avg in Inf)).

#### Protein–protein interaction networks

Prey proteins of the protein–protein interaction networks have been determined using a 10% threshold on the combined scores of the protein–protein interactions with the bait proteins: proteins with scores CS1 >10% were selected (or resp. CS2 >10% in function of the displayed network). Already known interactions in the STRING database (Szklarczyk et al, 2023) between the shortlisted prey proteins were downloaded with the app stringApp (Doncheva et al, 2019) of Cytoscape (Shannon et al, 2003). Widths of the edges are a function of the probability that there is a true interaction with the bait proteins in the infected (or resp. non-infected in function of the displayed network) condition for the green edges, and they are a function of the combined score of STRING for the orange ones. The combined score of STRING reflects the confidence we can have in an interaction from known interactions listed in several databases.

### Statistical analysis

All graphs and statistical analysis were performed with GraphPad Prism 10 (GraphPad Software). No statistical method was used to predetermine sample size. The Kolmogorov–Smirnov test was used to assess the normality of all datasets. Boxes in box plots extend from the 25th to the 75th percentile, with a line at the median, whiskers extend from the 10th to the 90th percentile, and dots only show outlier values. Bar graphs show the mean ± sem.

## Supplementary information

Peer Review File

Dataset EV1

Dataset EV2

Dataset EV3

Movie EV1

Movie EV2

Movie EV3

Movie EV4

Movie EV5

Movie EV6

Source data Fig. 1

Source data Fig. 2

Source data Fig. 3

Source data Fig. 4

Source data Fig. 5

Source data Fig. 6

Source data Fig. 7

Source data Fig. 8

Expanded View Figures

## Data Availability

The LC-MS/MS proteomic data have been deposited to the ProteomeXchange Consortium via the PRIDE partner repository with the dataset identifier PXD045648 and are available following this link: https://www.ebi.ac.uk/pride/archive/projects/PXD045648. The source data of this paper are collected in the following database record: biostudies:S-SCDT-10_1038-S44319-026-00843-z.
