# Author Correction: Arf1 is involved in *Neisseria meningitidis*-induced cortical branched F-actin network reorganization

**DOI:** 10.1038/s44319-026-00935-w

**Published:** 2026-09-15

**Authors:** Margot Sahnine, Morgane Crochet, Stéphane Tachon, Sylvie Goussard, Esthel Pénard, Cyril Scandola, Magalie Duchateau, Quentin Giai Gianetto, Audrey Salles, Gaël Moneron, Léa Swistak, Arthur Charles-Orszag, Adeline Mallet, Mariette Matondo, Jean-Yves Tinevez, Daria Bonazzi, Anna Sartori-Rupp, Guillaume Duménil, Dorian Obino

**Affiliations:** 1 Institut Pasteur, Université Paris Cité, INSERM U1225, Pathogenesis of Vascular Infections Unit, F-75015 Paris, France; 2 Institut Pasteur, Université Paris Cité, NanoImaging Core Facility, F-75015 Paris, France; 3 Institut Pasteur, Université Paris Cité, Ultrastructural BioImaging Core Facility, F-75015 Paris, France; 4 Institut Pasteur, Université Paris Cité, Proteomics Core Facility, Mass Spectrometry for Biology Unit, UAR CNRS 2024, F-75015 Paris, France; 5 Institut Pasteur, Université Paris Cité, Bioinformatics and Biostatistics HUB, F-75015 Paris, France; 6 Institut Pasteur, Université Paris Cité, Photonic Bio-Imaging Unit, Centre de Ressources et Recherches Technologiques (UTechS-PBI, C2RT), F-75015 Paris, France; 7 Institut Pasteur, Université Paris Cité, CNRS UMR3571, Signaling and Receptors Dynamics Unit, F-75015 Paris, France; 8 Institut Pasteur, Université Paris Cité, CNRS UMR3691, Dynamics of Host-Pathogen Interactions Unit, F-75015 Paris, France; 9 Institut Pasteur, Université Paris Cité, Image Analysis Hub, F-75015 Paris, France; 10 Present Address: Department of Microbiology and Molecular Genetics, University of California, Davis, CA 95616 USA

## Abstract

**Figure:**
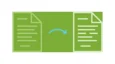

**Correction to:** *EMBO Reports* (2026) 27:4544–4582. https://doi.org/10.1038/s44319-026-00843-z | Published online 25 June 2026

**A citation is removed**.

**A citation is added**.

**Citation removed**

In the Results section “Cdc42 regulates Arp2/3 activity through N-WASP in endothelial protrusions induced by *N. meningitidis*”, the sentence:

“Strikingly, while previous reports have suggested the involvement of the small GTPase Cdc42, but not that of Rac1, in regulating F-actin reorganization in endothelial cells upon meningococcal infection (Coureuil et al, 2009; Eugene et al, 2002), others have rather shown a role for Rac1 (Coureuil et al, 2010; Lambotin et al, 2005)”.

Is corrected to: (changes in bold)

“Strikingly, while previous reports have suggested the involvement of the small GTPase Cdc42, but not that of Rac1, in regulating F-actin reorganization in endothelial cells upon meningococcal infection (Coureuil et al, 2009; Eugene et al, 2002), others have rather shown a role for Rac1 (**Lambotin et al, 2005**)”.

The citation of Coureuil et al, 2010 is removed, as this reference does not relate to the role of Rac1 in this context.

**Citation added**

In the Results section “F-actin reorganization in *N. meningitidis*-induced endothelial tubular protrusions relies on Arp2/3 activity”, the sentence:

“Equivalent results were obtained when assessing the local enrichment of endogenous Arp3 (Fig. EV2A-C) or that of the accessory subunit ArpC5-GFP transiently expressed in endothelial cells by live and fixed imaging experiments (Fig. 3D-G and Movie EV5)”.

Is corrected to: (changes in bold)

“Equivalent results were obtained when assessing the local enrichment of endogenous Arp3 (Fig. EV2A-C) or that of the accessory subunit ArpC5-GFP transiently expressed in endothelial cells by live and fixed imaging experiments (Fig. 3D-G and Movie EV5), **as previously reported for this subunit (Coureuil et al, 2009)**”.

The citation of Coureuil et al, 2009 was inadvertently omitted and is now added.

Both references remain cited elsewhere in the article; the reference list is unchanged. These changes do not affect the results or conclusions of the study.

