# Supplementary material for: Arf1 is involved in Neisseria meningitidis-induced cortical branched F-actin network reorganization
Source: EMBO Rep. 2026 Jun 25;27(15):4544–82. doi: 10.1038/s44319-026-00843-z (PMC13458072; doi:10.1038/s44319-026-00843-z)
Supplement: Supplementary file 1 [file 44319_2026_843_MOESM19_ESM.pdf]

Expanded View Figures

Figure EV1. Analysis pipeline used to quantify the enrichment of markers of interest underneath *N. meningitidis* aggregates.

To accurately quantify the local enrichment of the different markers of interest underneath bacterial aggregates on the apical surface of infected cells we developed a sequential approach. The multi-channel z-stack images (1) were resliced (2) and the focal planes containing bacteria-derived fluorescent signal were averaged projected (3). Following the manual drawing of the region of interest (ROI) along the apical surface of the cells (4), measurements of the raw fluorescence intensities in the different fluorescent channels were performed using Fiji (5). Data were then normalized with respect to the median value of the fluorescence intensity profile on the per cell basis (6) and centered according to the geometric center of the bacterial aggregate (7), hence allowing to average the profiles of various cells. Finally, the mean value of the normalized fluorescence intensities per channel and per cell in the non-infected and infected areas were extracted and plotted as paired observations (8).

© The Author(s)

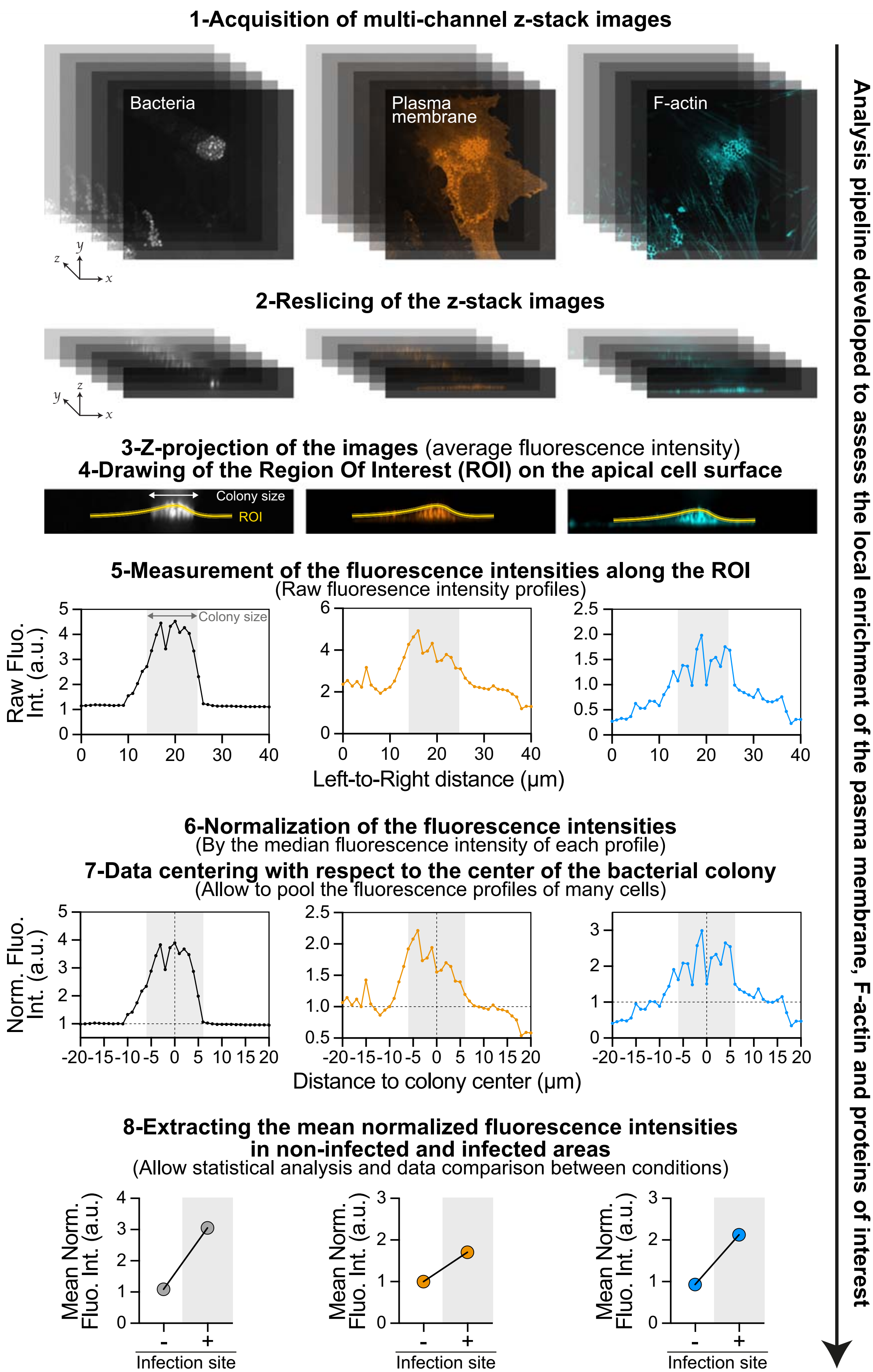
1-Acquisition of multi-channel z-stack images
Bacteria
Plasma membrane
F-actin
2-Reslicing of the z-stack images
3-Z-projection of the images (average fluorescence intensity)
4-Drawing of the Region Of Interest (ROI) on the apical cell surface
Colony size
ROI
5-Measurement of the fluorescence intensities along the ROI
(Raw fluoresence intensity profiles)
Raw Fluo. Int. (a.u.)
Colony size
Left-to-Right distance (µm)
6-Normalization of the fluorescence intensities
(By the median fluorescence intensity of each profile)
7-Data centering with respect to the center of the bacterial colony
(Allow to pool the fluorescence profiles of many cells)
Norm. Fluo. Int. (a.u.)
Distance to colony center (µm)
8-Extracting the mean normalized fluorescence intensities in non-infected and infected areas
(Allow statistical analysis and data comparison between conditions)
Mean Norm. Fluo. Int. (a.u.)
Infection site
Analysis pipeline developed to assess the local enrichment of the pasma membrane, F-actin and proteins of interest

© The Author(s)

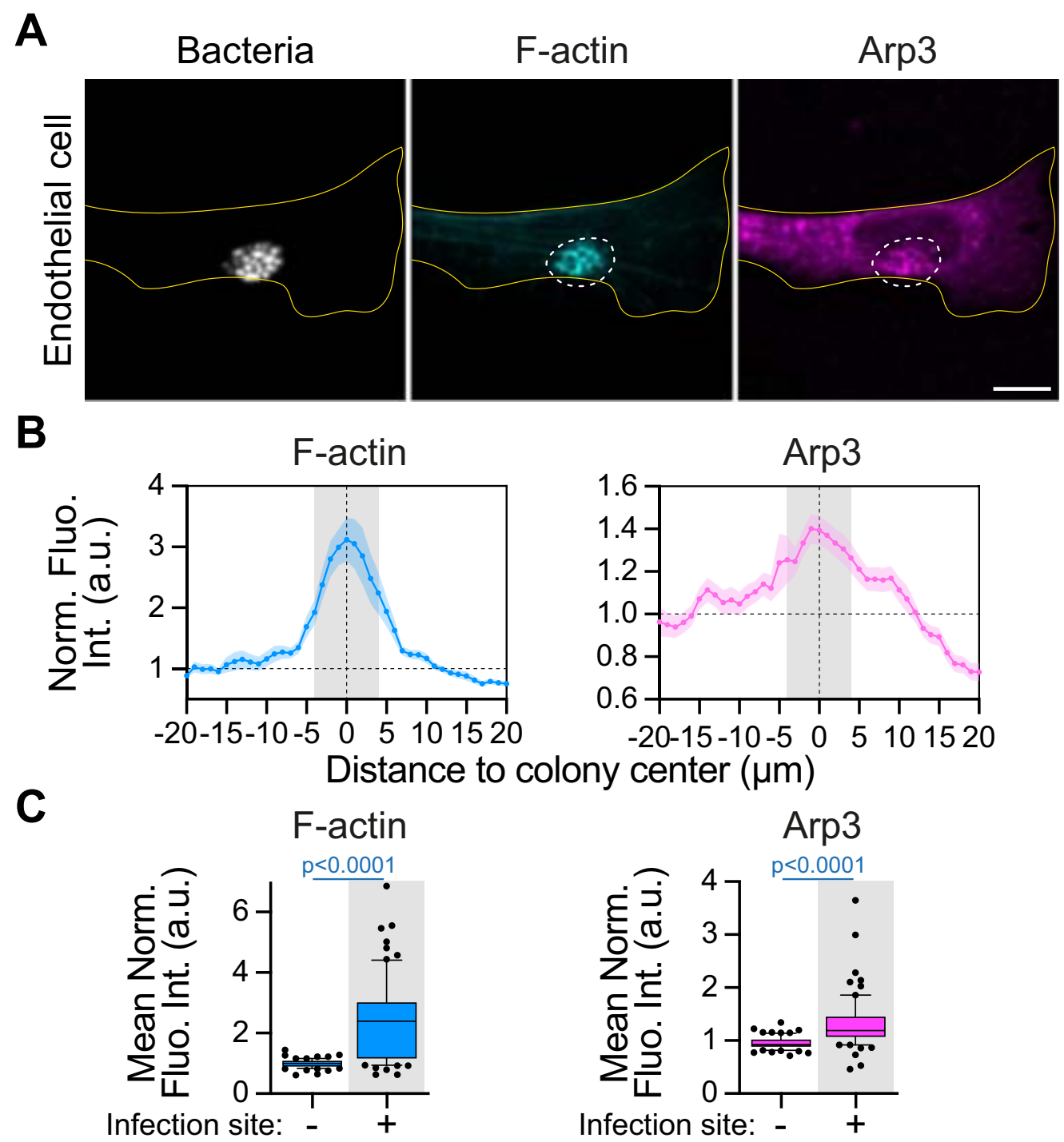

Figure EV2. Local enrichment of Arp2/3 in endothelial protrusions formed following *N. meningitidis*-induced plasma membrane deformation.

(**A**) Representative images (maximum intensity projections) of endothelial cells infected with BFP-expressing *N. meningitidis* (gray) and stained for F-actin (phalloidin, cyan) and endogenous Arp3 (specific antibody, magenta). The yellow lines delineate the cell contour. The white dashed lines delineate the contour of the bacterial aggregates. Scale bars: 10 µm. (**B**) Normalized fluorescence intensity profiles of F-actin and Arp3 in endothelial cells infected with BFP-expressing *N. meningitidis*. The light gray areas show the average colony size. Data are shown as mean ± SEM. (**C**) Mean value per cell of the normalized fluorescence intensity of F-actin and Arp3 in the non-infected (−) and infected (+) areas in endothelial cells infected with BFP-expressing *N. meningitidis*. Boxes in box plots extend from the 25th to the 75th percentile, with a line at the median, whiskers extend from the 10th to the 90th percentile, and dots show outlier values. The Wilcoxon matched-pairs signed-rank test was used to assess statistical significance. *P* values are shown on the graphs. (**A**–**C**) $n = 70$ infected endothelial cells, pooled from $N = 3$ biological replicates.

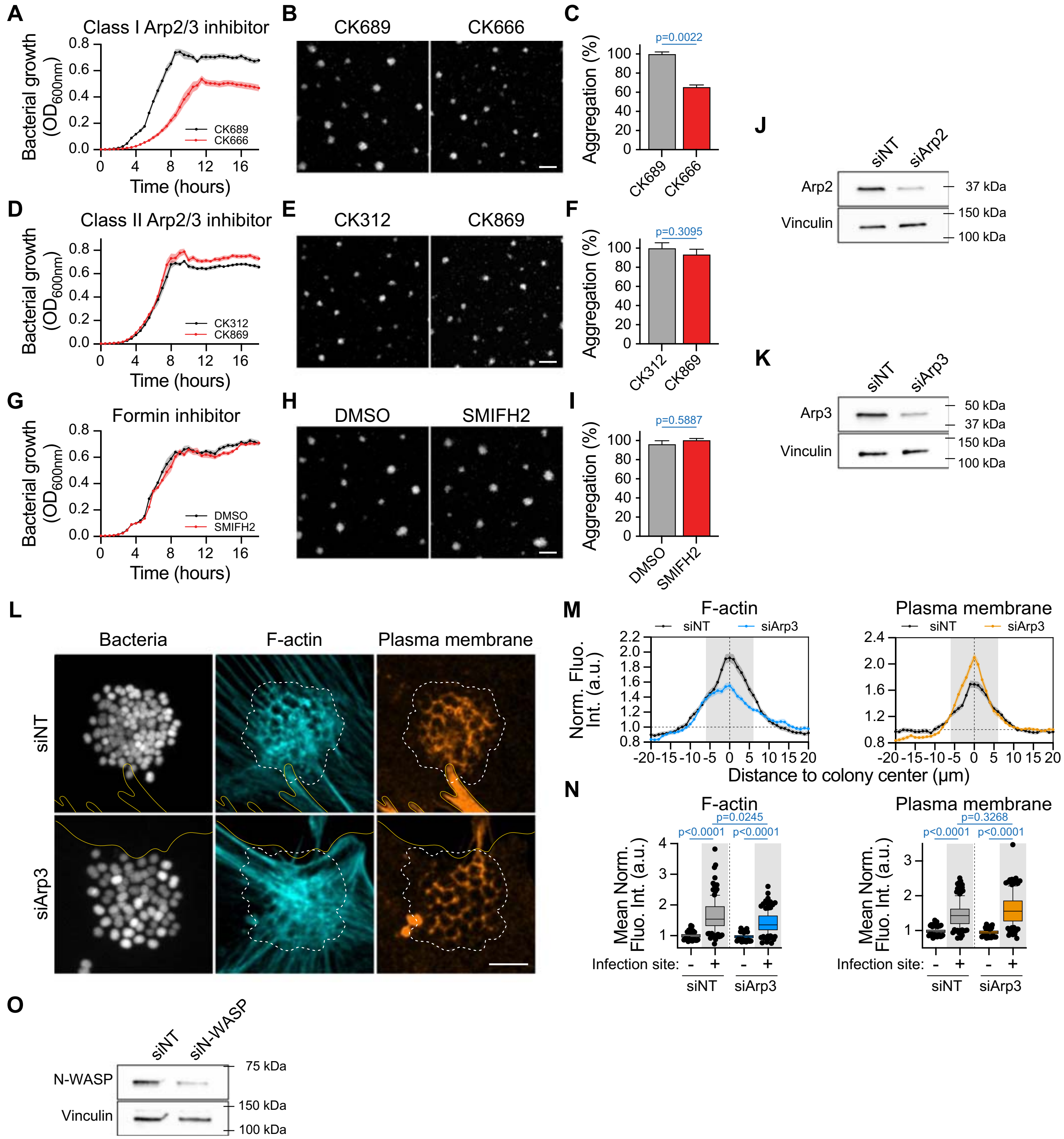
A
Class I Arp2/3 inhibitor
Bacterial growth (OD600nm)
CK689
CK666
Time (hours)
B
CK689
CK666
C
Aggregation (%)
p=0.0022
CK689
CK666
D
Class II Arp2/3 inhibitor
Bacterial growth (OD600nm)
CK312
CK869
Time (hours)
E
CK312
CK869
F
Aggregation (%)
p=0.3095
CK312
CK869
G
Formin inhibitor
Bacterial growth (OD600nm)
DMSO
SMIFH2
Time (hours)
H
DMSO
SMIFH2
I
Aggregation (%)
p=0.5887
DMSO
SMIFH2
J
siNT
siArp2
Arp2
Vinculin
37 kDa
150 kDa
100 kDa
K
siNT
siArp3
Arp3
Vinculin
50 kDa
37 kDa
150 kDa
100 kDa
L
Bacteria
F-actin
Plasma membrane
siNT
siArp3
M
F-actin
Plasma membrane
siNT
siArp3
Norm. Fluo. Int. (a.u.)
Distance to colony center (µm)
N
F-actin
Plasma membrane
p=0.0245
p<0.0001
p<0.0001
p=0.3268
p<0.0001
p<0.0001
Mean Norm. Fluo. Int. (a.u.)
Infection site: - + - +
siNT
siArp3
O
siNT
siN-WASP
N-WASP
Vinculin
75 kDa
150 kDa
100 kDa

© The Author(s)

Figure EV3. Silencing Arp2/3 expression impairs F-actin reorganization in endothelial tubular protrusions formed following *N. meningitidis*-induced plasma membrane deformation.

(**A**, **D**, **G**) Bacterial growth curves of *N. meningitidis* liquid cultures in the presence of (**A**) the Arp2/3 class I inhibitor CK666 or its corresponding inactive control compound CK689 (100 µM final); (**D**) the Arp2/3 class II inhibitor CK869 or its corresponding inactive control compound CK312 (20 µM final); or (**G**) the Formin inhibitor SMIFH2 and its vehicle control DMSO (1 µM final). The optical density at 600 nm of each culture was measured every 30 min for 18 h, each in technical triplicate. Data were pooled from $N = 3$ biological replicates and are displayed as the mean ± SEM. (**B**, **E**, **H**) Representative images of bacterial aggregates formed by GFP-expressing *N. meningitidis* following a 30 min incubation in the presence of either (**B**) CK689 or CK666 (100 µM final); (**E**) CK312 and CK869 (20 µM final); or (**H**) DMSO and SMIFH2 (1 µM final). One representative, out of $N = 2$ biological replicates, is shown. (**C**, **F**, **I**) The surface covered by bacterial aggregates was quantified in each field of view (three images per condition) using an in-house developed Fiji macro and normalized with respect to the mean value of the control condition (CK689, CK312, or DMSO, respectively). The Mann–Whitney test was used to assess statistical significance. Bar graphs show the mean±sem of $n = 6$ measurements per condition, pooled from $N = 2$ biological replicates. *P* values are shown on the graphs. (**J**, **K**) Western blot analysis of the efficiency of (**J**) Arp2 and (**K**) Arp3 silencing. Vinculin was used as a loading control. The immunoblots shown are representative of $N = 4$ biological replicates. (**L**) Representative images (maximum intensity projections) of control (siNT) and Arp3 (siArp3)-silenced endothelial cells infected with iRFP-expressing *N. meningitidis* (gray) and stained for F-actin (phalloidin, cyan) and the plasma membrane (CD31, orange). The yellow lines delineate the cell contour. The white dashed lines delineate the contour of bacterial aggregates. Note that pictures of control (siNT) infected endothelial cells shown here are the same as in Fig. 4G. Scale bar: 5 µm. (**M**) Normalized fluorescence intensity profiles of F-actin and the plasma membrane in control (siNT) and Arp3 (siArp3)-silenced endothelial cells infected with iRFP-expressing *N. meningitidis*. The light gray areas show the average colony size. Data are shown as mean ± SEM. (**N**) Mean value per cell of the normalized fluorescence intensity of F-actin and the plasma membrane in the non-infected (−) and infected (+) areas in control (siNT) and Arp3 (siArp3)-silenced endothelial cells infected with iRFP-expressing *N. meningitidis*. Boxes in box plots extend from the 25th to the 75th percentile, with a line at the median, whiskers extend from the 10th to the 90th percentile, and dots show outlier values. Kruskal–Wallis test was used to assess statistical significance. *P* values are shown on the graphs. (**L–N**) $n = 146$ and 138 infected endothelial cells for siNT and siArp3, respectively, pooled from $N = 4$ biological replicates. (**O**) Western blot analysis of the efficiency of N-WASP silencing. Vinculin was used as a loading control. The immunoblot shown is representative of $N = 2$ biological replicates.

© The Author(s)

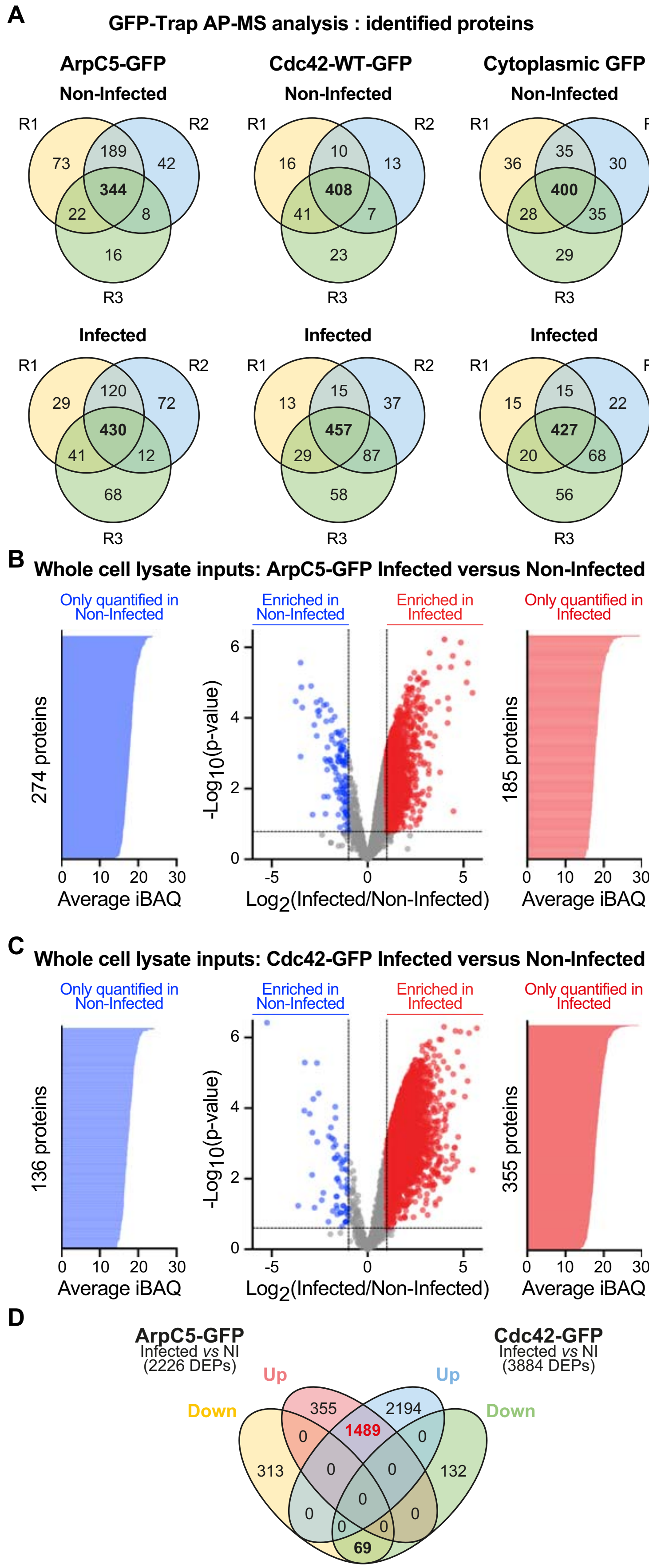
A
GFP-Trap AP-MS analysis : identified proteins
ArpC5-GFP
Cdc42-WT-GFP
Cytoplasmic GFP
Non-Infected
Infected
R1
R2
R3
73
189
42
344
22
8
16
16
10
13
408
41
7
23
36
35
30
400
28
35
29
29
120
72
430
41
12
68
13
15
37
457
29
87
58
15
15
22
427
20
68
56
B
Whole cell lysate inputs: ArpC5-GFP Infected versus Non-Infected
Only quantified in Non-Infected
Enriched in Non-Infected
Enriched in Infected
Only quantified in Infected
274 proteins
185 proteins
-Log10(p-value)
Log2(Infected/Non-Infected)
Average iBAQ
C
Whole cell lysate inputs: Cdc42-GFP Infected versus Non-Infected
136 proteins
355 proteins
D
ArpC5-GFP
Infected vs NI
(2226 DEPs)
Cdc42-GFP
Infected vs NI
(3884 DEPs)
Up
Down
355
2194
1489
313
132
69

 © The Author(s)

Figure EV4. ***N. meningitidis*** **infection modifies the proteomes of endothelial cells.**

(**A**) Venn diagrams showing the numbers and overlap of the proteins identified in each replicate (R1, R2, and R3) and condition following the GFP-Trap AP-MS analysis in non-infected (top) and *N. meningitidis*-infected (bottom) Arpc5-GFP-, Cdc42-WT-GFP-, or cytoplasmic GFP-expressing endothelial cells. (**B**, **C**) Pairwise comparisons of eukaryotic protein abundance in the whole cell lysates (inputs) of non-infected and *N. meningitidis*-infected ArpC5-GFP- (**B**) or Cdc42-WT-GFP- (**C**) expressing endothelial cells. *Left:* Bar graphs showing the proteins only quantified in the non-infected conditions (blue). Middle: Volcano plots showing the proteins differentially expressed in endothelial cells between non-infected and infected conditions. The horizontal dashed lines represent the threshold used for statistical significance (FDR ≤1%). The vertical dashed lines represent the biological threshold used to select differentially expressed proteins (absolute fold change ≥2). Blue and red dots show proteins whose expression is significantly increased in either the non-infected or the infected conditions, respectively. Right: Bar graphs showing the proteins only quantified in the infected conditions (red). Statistical testing was conducted using the limma *t*-test thanks to the R package limma and an adaptive Benjamini–Hochberg procedure was applied on the resulting *p* values thanks to the function adjust.p of the cp4p R package. (**D**) Venn diagram showing the overlap of the selected proteins (red + blue) among the two pairwise comparisons. (**A**–**D**) Data were obtained from three technical replicates per condition.

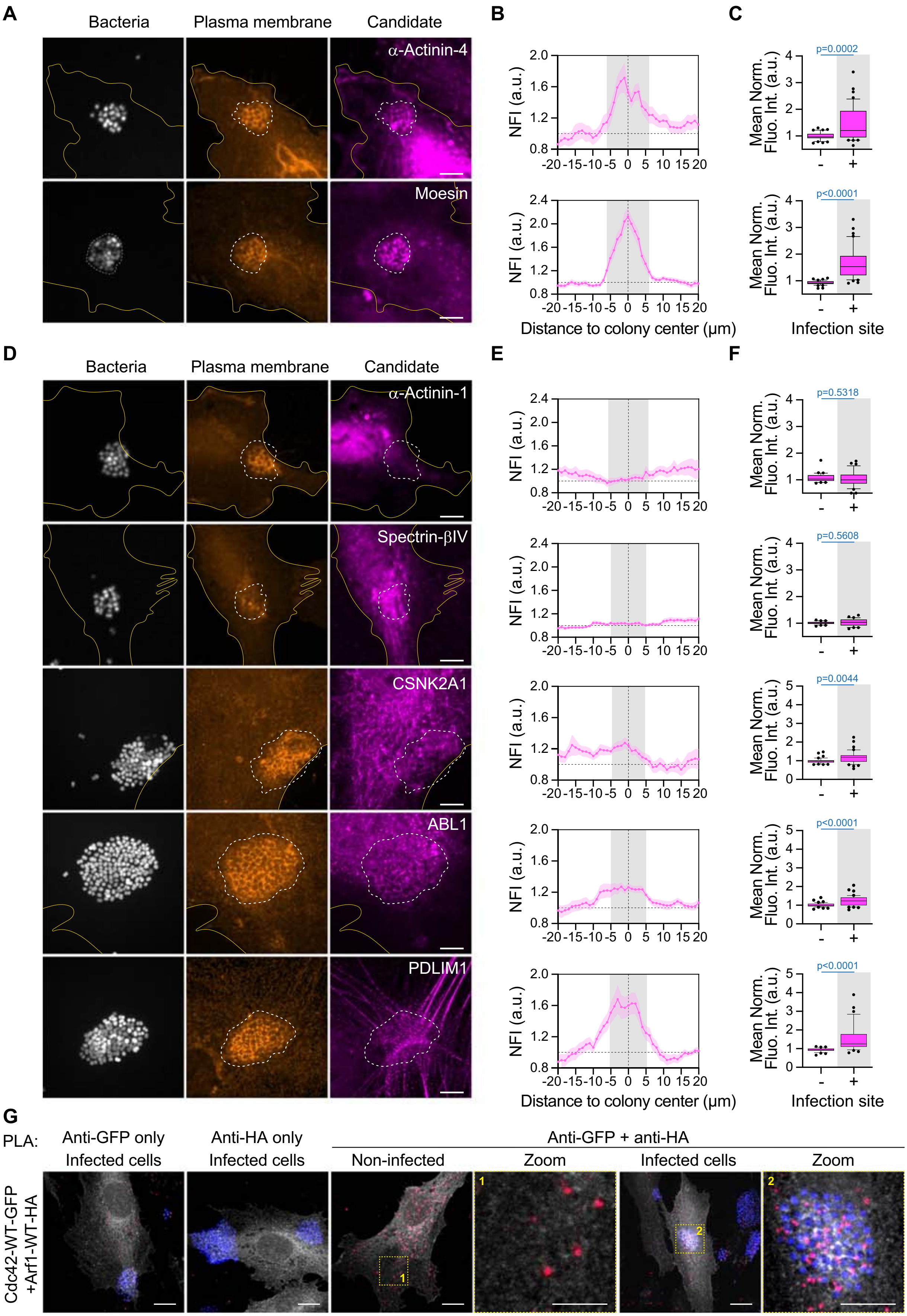
A
Bacteria
Plasma membrane
Candidate
α-Actinin-4
Moesin
B
NFI (a.u.)
Distance to colony center (µm)
C
Mean Norm. Fluo. Int. (a.u.)
p=0.0002
p<0.0001
Infection site
D
Bacteria
Plasma membrane
Candidate
α-Actinin-1
Spectrin-βIV
CSNK2A1
ABL1
PDLIM1
E
NFI (a.u.)
Distance to colony center (µm)
F
Mean Norm. Fluo. Int. (a.u.)
p=0.5318
p=0.5608
p=0.0044
p<0.0001
p<0.0001
Infection site
G
PLA:
Anti-GFP only
Infected cells
Anti-HA only
Infected cells
Anti-GFP + anti-HA
Non-infected
Zoom
Infected cells
Zoom
Cdc42-WT-GFP
+Arf1-WT-HA

© The Author(s)

Figure EV5. Immunofluorescence analysis of the local enrichment of ArpC5- and Cdc42-interacting protein candidates underneath bacterial microcolonies.

(**A**, **D**) Representative images (maximum intensity projections) of endothelial cells transiently expressing the plasma membrane probe PM-GFP (orange), infected with iRFP-expressing *N. meningitidis* (gray) and stained for either endogenous (**A**) α-Actinin-4 or Moesin, and (**D**) α-Actinin-1, Spectrin-βIV, CSNK2A1, ABL1, or PDLIM1 (each using specific antibodies, magenta). The yellow lines delineate the cell contour. The white dashed lines delineate the contour of bacterial aggregates. Scale bars: 5 µm. (**B**, **E**) Normalized fluorescence intensity profiles of (**B**) α-Actinin-4 and Moesin, and (**E**) α-Actinin-1, Spectrin-βIV, CSNK2A1, ABL1, and PDLIM1 in PM-GFP-expressing endothelial cells infected with iRFP-expressing *N. meningitidis*. The light gray areas show the average colony size. Data are shown as mean ± SEM. (**C**, **F**) Mean value per cell of the normalized fluorescence intensity of (**C**) α-Actinin-4 and Moesin, and (**F**) α-Actinin-1, Spectrin-βIV, CSNK2A1, ABL1, and PDLIM1 in the non-infected (−) and infected (+) areas in PM-GFP-expressing endothelial cells infected with iRFP-expressing *N. meningitidis*. Boxes in box plots extend from the 25th to the 75th percentile, with a line at the median, whiskers extend from the 10th to the 90th percentile, and dots show outlier values. The Wilcoxon matched-pairs signed-rank test was used to assess statistical significance. *P* values are shown on the graphs. (**A**–**F**) $n = 41$, 46, 34, 32, 40, 42, and 36 infected endothelial cells for α-Actinin-4, Moesin, α-Actinin-1, Spectrin-βIV, CSNK2A1, ABL1, and PDLIM1, respectively, each pooled from $N = 2$ biological replicates. (**G**) Representative images (maximum intensity projections) of a Proximity Ligation Assay (red) showing the interaction of Cdc42 and Arf1 in endothelial cells transiently co-expressing the wild-type versions of Cdc42 fused to GFP (gray) and Arf1 fused to HA (not displayed), and infected (right) or not (middle right) with iRFP-expressing *N. meningitidis* (blue). Control conditions using only one of the primary antibodies are shown on the middle left (anti-HA) and left (anti-GFP) panels, respectively. The yellow dashed squares delineate the zoomed areas in non-infected (1) and infected (2) cells shown on the right of the corresponding image. Scale bars: 10 and 5 µm, respectively. Representative of $n = 30$ cells per condition, pooled from $N = 2$ biological replicates.

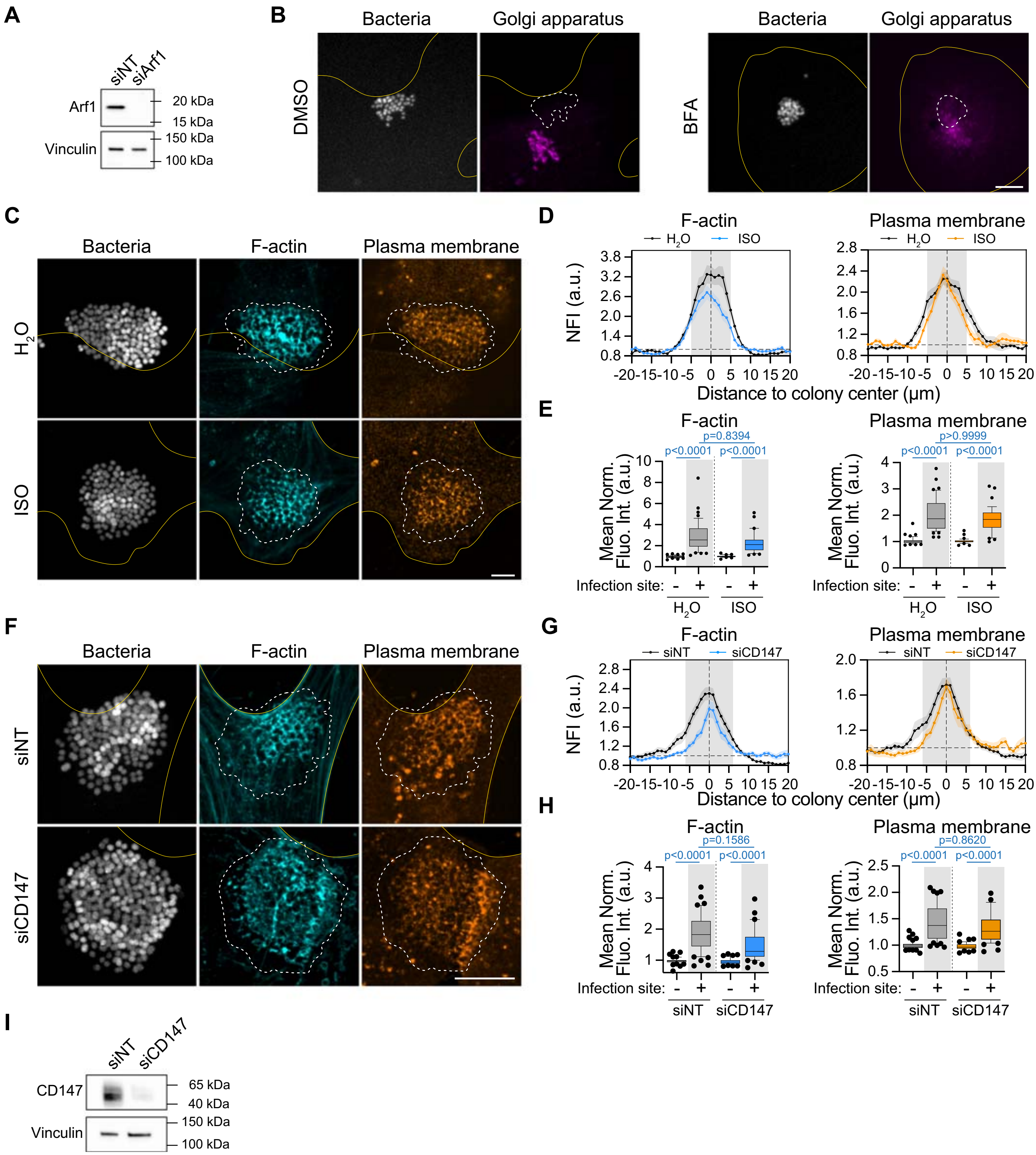
A
siNT
siArf1
Arf1
20 kDa
15 kDa
Vinculin
150 kDa
100 kDa
B
Bacteria
Golgi apparatus
DMSO
BFA
C
Bacteria
F-actin
Plasma membrane
H_2O
ISO
D
F-actin
H_2O
ISO
Plasma membrane
NFI (a.u.)
Distance to colony center (µm)
E
F-actin
p=0.8394
p<0.0001
p>0.9999
Plasma membrane
Mean Norm. Fluo. Int. (a.u.)
Infection site:
F
Bacteria
F-actin
Plasma membrane
siNT
siCD147
G
F-actin
Plasma membrane
NFI (a.u.)
Distance to colony center (µm)
H
F-actin
p=0.1586
p<0.0001
Plasma membrane
p=0.8620
Mean Norm. Fluo. Int. (a.u.)
Infection site:
I
siNT
siCD147
CD147
65 kDa
40 kDa
Vinculin
150 kDa
100 kDa

© The Author(s)

Figure EV6. The Arf1-Cdc42-N-WASP-Arp2/3 axis is not induced upon the activation of the β2-AR and CD147 cell-surface receptors.

(**A**) Western blot analysis of the efficiency of Arf1 silencing. Vinculin was used as a loading control. The immunoblots shown are representative of $N = 2$ biological replicates. (**B**) Representative images (maximum intensity projections) of endothelial cells infected with iRFP-expressing *N. meningitidis* (gray), treated prior and during infection with either control (DMSO) or Brefeldin A (BFA), and stained for the Golgi apparatus (GM130, magenta). The yellow lines delineate the cell contour. The white dashed lines delineate the contour of bacterial aggregates. Scale bar: 10 µm. Representative of $n = 32$ infected cells, pooled from $N = 2$ biological replicates. (**C**) Representative images (maximum intensity projections) of endothelial cells infected with iRFP-expressing *N. meningitidis* (gray), treated prior and during infection with either H_2O (control) or Isoproterenol (ISO), and stained for F-actin (phalloidin, cyan) and the plasma membrane (CD31, orange). The yellow lines delineate the cell contour. The white dashed lines delineate the contour of bacterial aggregates. Scale bar: 5 µm. (**D**) Normalized fluorescence intensity profiles of F-actin and the plasma membrane in endothelial cells treated with either H_2O or ISO and infected with iRFP-expressing *N. meningitidis*. The light gray areas show the average colony size. Data were shown as mean ± SEM. (**E**) Mean value per cell of the normalized fluorescence intensity of F-actin and the plasma membrane in the non-infected (−) and infected (+) areas in endothelial cells treated with either H_2O or ISO and infected with iRFP-expressing *N. meningitidis*. The Kruskal–Wallis test was used to assess statistical significance. (**C–E**) $n = 48$ and 36 infected endothelial cells for H_2O and ISO, respectively, pooled from $N = 2$ biological replicates. (**F**) Representative images (maximum intensity projections) of control (siNT) and CD147 (siCD147)-silenced endothelial cells infected with iRFP-expressing *N. meningitidis* (gray), and stained for F-actin (phalloidin, cyan) and the plasma membrane (CD31, orange). The yellow lines delineate the cell contour. The white dashed lines delineate the contour of bacterial aggregates. Scale bar: 5 µm. (**G**) Normalized fluorescence intensity profiles of F-actin and the plasma membrane in control (siNT) and CD147 (siCD147)-silenced endothelial cells infected with iRFP-expressing *N. meningitidis*. The light gray areas show the average colony size. Data were shown as mean ± SEM. (**H**) Mean value per cell of the normalized fluorescence intensity of F-actin and the plasma membrane in the non-infected (−) and infected (+) areas in control (siNT) and CD147 (siCD147)-silenced endothelial cells infected with iRFP-expressing *N. meningitidis*. Kruskal–Wallis test was used to assess statistical significance. (**F–H**) $n = 51$ and 42 infected cells for siNT and siCD147, respectively, pooled from $N = 2$ biological replicates. (**E**, **H**) Boxes in box plots extend from the 25th to the 75th percentile, with a line at the median, whiskers extend from the 10th to the 90th percentile, and dots show outlier values. *P* values are shown on the graphs. (**I**) Western blot analysis of the efficiency of CD147 silencing. Vinculin was used as a loading control. The immunoblots shown are representative of $N = 2$ biological replicates.
