# Supplementary material for: Arf1 is involved in Neisseria meningitidis-induced cortical branched F-actin network reorganization
Source: EMBO Rep. 2026 Jun 25;27(15):4544–82. doi: 10.1038/s44319-026-00843-z (PMC13458072; doi:10.1038/s44319-026-00843-z)
Supplement: Supplementary file 2 [file 44319_2026_843_MOESM1_ESM.pdf]

EMBO reports

Arf1 is involved in Neisseria meningitidis-induced cortical branched F-actin network reorganization

Margot Sahnine, Morgane Crochet, Stéphane Tachon, Sylvie GOUSSARD, Esthel Pénard, Cyril Scandola, Magalie Duchateau, Quentin Giai Gianetto, Audrey Salles, Gaël Moneron, Léa Swistak, Arthur Charles-Orszag, Adeline Mallet, Mariette Matondo, Jean-Yves Tinevez, Daria Bonazzi, Anna Sartori-Rupp, Guillaume Dumenil, and Dorian Obino

Corresponding author(s): Dorian Obino (dorian.obino@inserm.fr) , Guillaume Dumenil (guillaume.dumenil@pasteur.fr)

Review Timeline:

Submission Date:	15th Oct 25
Editorial Decision:	12th Nov 25
Revision Received:	27th Mar 26
Editorial Decision:	29th Apr 26
Revision Received:	22nd May 26
Accepted:	8th Jun 26

Editor: Achim Breiling

Transaction Report:

(Note: With the exception of the correction of typographical or spelling errors that could be a source of ambiguity, letters and reports are not edited. Depending on transfer agreements, referee reports obtained elsewhere may or may not be included in this compilation. Referee reports are anonymous unless the Referee chooses to sign their reports.)

1st Editorial Decision **12th Nov 2025**

Dear Dr. Obino,

Thank you for the transfer of your manuscript to EMBO reports. I have now received reports from the three referees that were asked to evaluate your study, which can be found at the end of this email. As you will see, the referees think that these findings are of interest. However, they have several comments, concerns, and suggestions, indicating that a major revision of the manuscript is necessary to allow publication of the study in EMBO reports. As the reports are below, and all the referee concerns need to be addressed, I will not detail them here.

Given the constructive referee comments, I would thus like to invite you to revise your manuscript with the understanding that the concerns of the referees must be addressed in the revised manuscript and/or in a detailed point-by-point response. Acceptance of your manuscript will depend on a positive outcome of a second round of review. It is EMBO reports policy to allow a single round of revision only and acceptance of the manuscript will therefore depend on the completeness of your responses included in the next, final version of the manuscript.

Revised manuscripts should be submitted within three months of a request for revision. Please contact me to discuss the revision (also by video chat) if you have questions or comments regarding the revision, or should you need additional time.

When submitting your revised manuscript, please also carefully review the instructions that follow below.

PLEASE NOTE THAT upon resubmission revised manuscripts are subjected to an initial quality control prior to exposition to re-review. Upon failure in the initial quality control, the manuscripts are sent back to the authors, which may lead to delays. Frequent reasons for such a failure are the lack of the data availability section (please see below) and the presence of statistics based on n=2 (the authors are then asked to present scatter plots or provide more data points).

When submitting your revised manuscript, we will require:

1) a .docx formatted version of the final manuscript text (including legends for main figures, EV figures and tables), but without the figures included. Figure legends should be compiled at the end of the manuscript text.

2) individual production quality figure files as .eps, .tif, .jpg (one file per figure), of main figures and EV figures. Please upload these as separate, individual files upon re-submission.

The Expanded View format, which will be displayed in the main HTML of the paper in a collapsible format, has replaced the Supplementary information. You can submit up to 6 images as Expanded View. Please follow the nomenclature Figure EV1, Figure EV2 etc. The figure legend for these should be included in the main manuscript document file in a section called Expanded View Figure Legends after the main Figure Legends section. Additional Supplementary material should be supplied as a single pdf file labeled Appendix. The Appendix should have page numbers and needs to include a table of content on the first page (with page numbers) and legends for all content. Please follow the nomenclature Appendix Figure Sx, Appendix Table Sx etc. throughout the text, and also label the figures and tables according to this nomenclature.

For more details, please refer to our guide to authors:
http://www.embopress.org/page/journal/14693178/authorguide#manuscriptpreparation

Please consult our guide for figure preparation:
http://wol-prod-cdn.literatumonline.com/pb-assets/embo-site/EMBOPress_Figure_Guidelines_061115-1561436025777.pdf

See also the guidelines for figure legend preparation:
https://www.embopress.org/page/journal/14693178/authorguide#figureformat

3) a .docx formatted letter INCLUDING the reviewers' reports and your detailed point-by-point responses to their comments. As part of the EMBO Press transparent editorial process, the point-by-point response is part of the Review Process File (RPF), which will be published alongside your paper.

4) a complete author checklist, which you can download from our author guidelines (https://www.embopress.org/page/journal/14693178/authorguide). Please insert page numbers in the checklist to indicate where the requested information can be found in the manuscript. The completed author checklist will also be part of the RPF.

Please also follow our guidelines for the use of living organisms, and the respective reporting guidelines:
http://www.embopress.org/page/journal/14693178/authorguide#livingorganisms

5) that primary datasets produced in this study (e.g. RNA-seq, ChIP-seq, structural and array data) are deposited in an appropriate public database. If no primary datasets have been deposited, please also state this in a dedicated section (e.g. 'No

primary datasets have been generated and deposited'), see below.

See also: http://embor.embopress.org/authorguide#datadeposition

Please remember to provide a reviewer password if the datasets are not yet public.

The accession numbers and database should be listed in a formal "Data Availability" section that follows the model below. This is now mandatory (like the COI statement). Please note that the Data Availability Section is restricted to new primary data that are part of this study. This section is mandatory. As indicated above, if no primary datasets have been deposited, please state this in this section

Data availability

The datasets produced in this study are available in the following databases:

- RNA-Seq data: Gene Expression Omnibus GSE46843 (https://www.ncbi.nlm.nih.gov/geo/query/acc.cgi?acc=GSE46843)
- [data type]: [name of the resource] [accession number/identifier/doi] ([URL or identifiers.org/DATABASE:ACCESSION])

*** Note - All links should resolve to a page where the data can be accessed. ***

Moreover, I have these editorial requests:

6) We now request the publication of original source data with the aim of making primary data more accessible and transparent to the reader. You will receive a separate email with instructions for providing source data with your revised manuscript, including information how to upload and organize the files.

7) Our journal encourages inclusion of *data citations in the reference list* to directly cite datasets that were re-used and obtained from public databases. Data citations in the article text are distinct from normal bibliographical citations and should directly link to the database records from which the data can be accessed. In the main text, data citations are formatted as follows: "Data ref: Smith et al, 2001" or "Data ref: NCBI Sequence Read Archive PRJNA342805, 2017". In the Reference list, data citations must be labeled with "[DATASET]". A data reference must provide the database name, accession number/identifiers and a resolvable link to the landing page from which the data can be accessed at the end of the reference. Further instructions are available at: http://www.embopress.org/page/journal/14693178/authorguide#referencesformat

8) Regarding data quantification and statistics, please make sure that the number "n" for how many independent experiments were performed, their nature (biological versus technical replicates), the bars and error bars (e.g. SEM, SD) and the test used to calculate p-values is indicated in the respective figure legends (also for EV and Appendix figures). Please also check that all the p-values are explained in the legend, and that these fit to those shown in the figure. Please provide statistical testing where applicable. Please avoid the phrase 'independent experiment' but clearly state if these were biological or technical replicates. Please also indicate (e.g. with n.s.) if testing was performed, but the differences are not significant. In case n=2, please show the data as separate datapoints without error bars and statistics. See also:
http://www.embopress.org/page/journal/14693178/authorguide#statisticalanalysis

If n<5, please show single datapoints for diagrams.

9) Please add scale bars of similar style and thickness to all microscopic images, using clearly visible black or white bars (depending on the background). Please place these in the lower right corner of the images themselves. Please do not write on or near the bars in the image but define the size in the respective figure legend.

10) Please also note our reference format:
http://www.embopress.org/page/journal/14693178/authorguide#referencesformat

11) We updated our journal's competing interests policy in January 2022 and request authors to consider both actual and perceived competing interests. Please review the policy https://www.embopress.org/competing-interests and update your competing interests if necessary. Please name this section 'Disclosure and Competing Interests Statement' and put it after the Acknowledgements section.

12) We now use CRediT to specify the contributions of each author in the journal submission system. CRediT replaces the author contribution section. Please use the free text box to provide more detailed descriptions and do NOT provide your final manuscript text file with an author contributions section. See also our guide to authors:
https://www.embopress.org/page/journal/14693178/authorguide#authorshipguidelines

13) All Materials and Methods need to be described in the main text using our 'Structured Methods' format, which is required for all research articles. According to this format, the Methods section should include a Reagents and Tools Table (listing key

reagents, experimental models, software, and relevant equipment and including their sources and relevant identifiers), uploaded as separate file, and a Methods section in which we encourage the authors to describe their methods using a step-by-step protocol format with bullet points, to facilitate the adoption of the methodologies across labs. More information on how to adhere to this format as well as downloadable templates (.doc) for the Reagents and Tools Table can be found in our author guidelines (section 'Structured Methods'):

https://www.embopress.org/page/journal/14693178/authorguide#manuscriptpreparation

14) Please add up to 5 keywords to the manuscript and order the manuscript sections like this, using only these names: Title page - Abstract - Keywords - Introduction - Results - Discussion - Methods - Data availability section - Acknowledgements (please include here all the funding information) - Disclosure and Competing Interests Statement - References - Figure legends - Expanded View Figure legends

15) Please make sure that all the funding information is also entered into the online submission system and that it is complete and similar to the one in the acknowledgement section of the manuscript text file.

We would also welcome the submission of cover suggestions, or motifs to be used by our Graphics Illustrator in designing a cover.

As part of the EMBO publication's Transparent Editorial Process, EMBO reports publishes online a Review Process File (RPF) to accompany accepted manuscripts. This File will be published in conjunction with your paper and will include the referee reports, your point-by-point response and all pertinent correspondence relating to the manuscript.

You are able to opt out of this by letting the editorial office know (emboreports@embo.org). If you do opt out, the Review Process File link will point to the following statement: "No Review Process File is available with this article, as the authors have chosen not to make the review process public in this case."

I look forward to seeing a revised form of your manuscript when it is ready.

Yours sincerely,

Achim Breiling
Senior Editor
EMBO Reports

Referee #1:

In this paper by Sahnine et al., the authors continue to explore the mechanism of plasma membrane protrusion formation during N. meningitidis adherence to endothelial cells, a process they uncovered in previous studies. While the premise of the paper and the importance of the subject are worth investigating, the paper only makes small incremental discoveries. The contribution of the small GTPase Arf1 to the subsequent reorganization of a cortical branched F-actin network in endothelial cells is not surprising, since the role of Arf1 in branched actin assembly is known.

Below are a few outstanding questions:

(1) The critical question is what is the role of branches actin polymerization underneath plasma remodeling and protrusion formation? The authors have previously reported and mentioned in this manuscript that the membrane remodeling itself is the key mechanism to bacteria resisting sheer stress as inhibition of actin polymerization doesn't have an effect on membrane reorganization or bacteria resistance to flow. A previous paper detailing the membrane wetting mechanism showed that plasma membrane protrusions are induced in significantly higher numbers by Nm in cytochalasin D-treated cells but have shorter lifetimes. Is the retraction than dependent on actin polymerization and is the Arf1/Cdc42-NWASP-Arp2/3 pathway important?
(2) As the wetting mechanism is dependent on T4P on the bacteria, is the subsequent actin polymerization and the assembly of the Arf1/Cdc42-NWASP-Arp2/3 pathway also dependent on T4P?
(3) Related to question 2, are bacteria used for infection piliated or a mixed population? I am assuming they are piliated, but the question then becomes why the graphs detailing the fluorescence intensity profiles of plasma membrane, F-actin and even Arp2, ArpC5, N-WASP show about 50% of bacteria with no changes. Since the remodeling was demonstrated to occur at the level of single bacteria is this due to piliation or stochastic, time-dependent events?
(4) The authors do not provide direct evidence for Arf1-Cdc42 interaction. They mentioned that specific antibodies are unavailable and that successive siRNA transfection and expression of GFP fusion proteins lead to excessive toxicity in primary endothelial cells. However, antibodies for endogenous Cdc42 are available (Cell Signaling) and they clearly showed that overexpression of GFP fusion proteins is not toxic. Therefore, co-immunoprecipitation experiments for Cdc42 and GFP-Arf1 under non-infection and infection conditions are possible.
(5) Overall, the paper is beautifully designed and very well written. Nevertheless, the issues raised above must be adequately

addressed prior to acceptance for publication.

Referee #2:

In this manuscript the authors investigate actin reorganization in epithelial (HNEpC) and endothelial (HUVEC) cells following mechanical membrane deformation caused by Neisseria meningitidis (Nm). Using fluorescence and electron microscopy techniques they show that adhesion of Nm leads to plasma membrane enrichment under bacterial aggregates in both endothelial and epithelial cells. These membrane protrusions were longer and thicker in endothelium compared to epithelium. Whereas epithelial protrusions contained parallel F-actin filaments (suggesting involvement of Formin family members), endothelial protrusions enclosed a branches F-actin network (suggesting a role for Arp2/3).

Subsequently the authors identify the cellular factors involved in branched actin filament formation in endothelia. Using high resolution photonic microscopy, genetic and drug perturbations, and proteomic analysis, they find that Arp2/3, N-WASP downstream of Cdc42, and the small GTPase Arf1 are involved, pointing to an Arf1/Cdc42-N-WASP-Arp2/3 pathway.

This is a very interesting manuscript addressing an important aspect of the host cell response to Nm. The performed experiments involve sophisticated methods, and are thoroughly conducted, interpreted, and discussed. The obtained results are of general interest and can initiate future analyses.

I have the following points to address:

- Infection experiments: Please provide an explanation why different MOIs and infection times were used for infection of endothelial and epithelial cells. Were endothelial and epithelial cells tested for viability following infection with Nm? Live cell imaging in Figure 3D and Figure 5G were performed with preformed bacterial aggregates, this is not mentioned in the legend to Figure 5G. Please include in the Materials and Methods how infection with preformed aggregates was done.

- The authors identified PDLIM and ABL1 during the AP-MS approach and could validate a local enrichment of PDLIM and ABL1 underneath bacterial microcolonies. Although they could not achieve efficient silencing of these proteins, they should briefly address the possible roles of these proteins in the discussion.

- I do not find a reference to Figure 9 in the manuscript text.

Referee #3:

This is a beautiful electron-microscopy description of how cell membranes and the underlying cytoskeleton form tubular protrusions, induced by Neisseria meningitidis. The main result of this study is that actin networks form completely differently in endothelial and epithelial cells.
I have some critics about the interpretation of some of the results, and the lack of clarity about some implicit statements.

One dimensional wetting (1DW) is an interpretation that has no relevance here, as none of the possible parameters of this model are used in analysing the results. The use of "membrane protrusions formed by 1DW" is obscure without challenging the model. "Tubular membranes" should be sufficiently explicit to understand what are those protrusions.

I do not understand the sense of this sentence: "our data highlight distinct mechanisms that support the formation of linear actin bundles in epithelial cell-derived protrusions, but an Arp2/3-dependent branched F-actin network".

About Fig 1A-C and Fig 2A-C about the enrichment of the plasma membrane, it is stated that the enrichment is "to a lesser extent in infected epithelial cells compared to infected endothelial cells (Fig. 1A-C)". Same in Figure 2 A-C. Is the total fluorescence intensity a good measure? The apparent difference of the distributions may be due to cluster size, the total plasma membrane or actin amount being constant. By the way, this cluster size difference would support the different organisation of actin that is further observed.

"Nm" should be defined

"deformation of the plasma membrane by 1DW upon N. meningitidis adhesion at the surface of epithelial or endothelial cells triggers the formation of..." I do not see how this "trigger" mechanism can be demonstrated. The authors do not show what precedes what. Actin polymerisation could very well deform the membrane, especially through the ARP2/3 branching mechanism, as has been shown in reconstituted systems (Simon, Nature Physics 2019)

"a given cell type will preferentially utilize either Arp2/3- or Formin-dependent actin nucleation machineries in response to membrane deformation" Again, the fact that membrane deformation precedes actin polymerisation is not clearly demonstrated. Is it important here to force this chronology that way?

Point-by-Point Response to the Referees

We thank the Referees for their constructive comments that have participated in increasing the quality of the revised version of our manuscript. Provided below in blue is our point-by-point response to the referees' comments.

Referee #1:

In this paper by Sahnine et al., the authors continue to explore the mechanism of plasma membrane protrusion formation during N. meningitidis adherence to endothelial cells, a process they uncovered in previous studies. While the premise of the paper and the importance of the subject are worth investigating, the paper only makes small incremental discoveries. The contribution of the small GTPase Arf1 to the subsequent reorganization of a cortical branched F-actin network in endothelial cells is not surprising, since the role of Arf1 in branched actin assembly is known.

We thank Referee #1 for his/her comments. While the signaling pathway leading to meningococci-induced actin reorganization has been the topic of several studies, our understanding of this process was still fragmentary. We here integrate signaling within the framework of the idea of 1D wetting and F-actin reorganization, defining the sequence of the events.
We would like to argue that the role of a plasma membrane pool of Arf1 in this process is not incremental but rather entirely unexpected. The identification (proteomics, immunofluorescence, siRNA-mediated silencing, chemical inhibition) of Arf1 as a central regulator of F-actin reorganization in endothelial protrusions upon meningococcal infection was not known or ever speculated in a manuscript. Although Arf1-mediated regulation of Arp2/3 activity through Cdc42 was described at the Golgi apparatus or at the plasma membrane in CLIC/GEEC endocytosis, the role of Arf1 is entirely new in the field of *N. meningitidis* infection. This opens new avenues of research by establishing links with other biological processes such as CLIC/GEEC endocytosis, a connection never before considered.
Responses to the specific points raised by Referee #1 are provided below.

Below are a few outstanding questions:
(1) The critical question is what is the role of branches actin polymerization underneath plasma remodeling and protrusion formation? The authors have previously reported and mentioned in this manuscript that the membrane remodeling itself is the key mechanism to bacteria resisting sheer stress as inhibition of actin polymerization doesn't have an effect on membrane reorganization or bacteria resistance to flow. A previous paper detailing the membrane wetting mechanism showed that plasma membrane protrusions are induced in significantly higher numbers by Nm in cytochalasin D-treated cells but have shorter lifetimes. Is the retraction than dependent on actin polymerization and is the Arf1/Cdc42-NWASP-Arp2/3 pathway important?

We thank Referee #1 for this insightful comment. As demonstrated in our previous publication (Charles-Orszag *et al.*, Nat Commun., 2018), the reorganization of the F-actin network in *Nm*-induced protrusions allows the stabilization of the newly formed protrusions. Treatment with Cytochalasin D to prevent actin assembly resulted in higher numbers of protrusions with shorter lifetimes, suggesting that

coalescence/retraction of the protrusions is independent of F-actin assembly within the protrusions. The role of actin reorganization following meningococcal adhesion was thus previously explored and does not warrant additional experiments in our view. **The goal of the current study is to better define the signaling pathway regulating F-actin reorganization within *Nm*-induced protrusions.**

(2) As the wetting mechanism is dependent on T4P on the bacteria, is the subsequent actin polymerization and the assembly of the Arf1/Cdc42-NWASP-Arp2/3 pathway also dependent on T4P?
We thank Referee #1 for bringing this important question to our attention. We now provide experimental evidence that meningococcal type IV pili are required to induce the local recruitment of Arf1, as revealed by the dose-dependent decrease of Arf1-positive bacterial microcolonies when endothelial cell infections were performed in the presence of increasing concentrations of a signaling-blocking anti-T4P antibody (clone 20D9, Charles-Orszag *et al.*, Nat Commun., 2018). **These results are now included in Revised Figure 7F-G (manuscript lines 398-403).**

In addition, to deepen our understanding of the signaling cascade, we performed rescue experiments by co-transfecting endothelial cells with a constitutive active mutant of Arf1 together with the dominant negative mutant version of Cdc42 used in Fig. 5D-F. No rescue was observed, strongly supporting that Arf1 acts upstream of Cdc42 in regulating the N-WASP-dependent activation of the Arp2/3 complex in *Nm*-induced plasma membrane protrusions. A similar order of involvement is seen in other instances such as CLIC-GEEC endocytosis (Kumari and Mayor, Nat Cell Biol, 2008) or Golgi-associated actin polymerization (Adarska et al, Front Cell Dev Biol, 2021). **These results are now included in Revised Fig. 8G-I (manuscript lines 420-430).**

(3) Related to question 2, are bacteria used for infection piliated or a mixed population? I am assuming they are piliated, but the question then becomes why the graphs detailing the fluorescence intensity profiles of plasma membrane, F-actin and even Arp2, ArpC5, N-WASP show about 50% of bacteria with no changes. Since the remodeling was demonstrated to occur at the level of single bacteria is this due to piliation or stochastic, time-dependent events?
Bacteria used for all experiments are indeed piliated. We apologize if the graphical representation we used was not sufficiently clear. Boxes in box plots extend from the 25th to the 75th percentile, with a line at the median, whiskers extend from the 10th to the 90th percentile, and individual dots show outlier values (below and above the 10th and 90th percentiles, respectively). **Explanation of the graphical representations used has been updated in the Methods section (Statistical analysis subheading) and the figure legends to enhance clarity.**

(4) The authors do not provide direct evidence for Arf1-Cdc42 interaction. They mentioned that specific antibodies are unavailable and that successive siRNA transfection and expression of GFP fusion proteins lead to excessive toxicity in primary endothelial cells. However, antibodies for endogenous Cdc42 are available (Cell Signaling) and they clearly showed that overexpression of GFP fusion proteins is not toxic. Therefore, co-immunoprecipitation experiments for Cdc42 and GFP-Arf1 under non-infection and infection conditions are possible.

Evidence for the interaction between Arf1 and Cdc42 is provided by our mass spectrometry analysis of Cdc42-interacting proteins following Cdc42-GFP pulldown and the subsequent SAINT analysis (Fig. 7 and Fig. EV6), which confidently and significantly identified Arf1 as interacting with Cdc42 in *Nm*-infected endothelial cells.

To confirm the interaction between both proteins as requested by Referee #1 and rather than using a co-immunoprecipitation approach that lacks spatial information and is redundant with our GFP pull down assays, we now provide data of immunofluorescence by Proximity Ligation Assay (Hegazy M, et al., Curr Protoc Cell Biol., 2021) to highlight Arf1-Cdc42 interaction at the subcellular level. Co-transfecting endothelial cells with Cdc42-GFP and Arf1-HA allows us to confirm that both proteins locally interact underneath *Nm* bacterial aggregates. **These data are now included in Revised Fig. EV7G (manuscript lines 387-390).**

We would like to point out that Cdc42 and Arf1 are unlikely to interact directly. They probably interact through intermediate proteins such as the coatomer or ARHGAP21/ARHGAP10 (Myers and Casanova, Trends in Cell Biol. 2008). Such proteins are seen in our pull down (COPBI) and could provide a bridge although this will need to be further explored in future studies. **This point is now clarified in the Discussion (manuscript lines 498-503).**

(5) Overall, the paper is beautifully designed and very well written. Nevertheless, the issues raised above must be adequately addressed prior to acceptance for publication. We thank Referee #1 for his/her supportive comment on the publication of our work and sincerely hope that the experimental work provided in the revised version of our manuscript adequately answers his/her concerns.

Referee #2:

In this manuscript the authors investigate actin reorganization in epithelial (HNEpC) and endothelial (HUVEC) cells following mechanical membrane deformation caused by Neisseria meningitidis (Nm). Using fluorescence and electron microscopy techniques they show that adhesion of Nm leads to plasma membrane enrichment under bacterial aggregates in both endothelial and epithelial cells. These membrane protrusions were longer and thicker in endothelium compared to epithelium. Whereas epithelial protrusions contained parallel F-actin filaments (suggesting involvement of Formin family members), endothelial protrusions enclosed a branches F-actin network (suggesting a role for Arp2/3).

Subsequently the authors identify the cellular factors involved in branched actin filament formation in endothelia. Using high resolution photonic microscopy, genetic and drug perturbations, and proteomic analysis, they find that Arp2/3, N-WASP downstream of Cdc42, and the small GTPase Arf1 are involved, pointing to an Arf1/Cdc42-N-WASP-Arp2/3 pathway.

This is a very interesting manuscript addressing an important aspect of the host cell response to Nm. The performed experiments involve sophisticated methods, and are thoroughly conducted, interpreted, and discussed. The obtained results are of general interest and can initiate future analyses.

We thank Referee #2 for the supportive comments. Responses to the specific points he/she raised are provided below.

I have the following points to address:

- Infection experiments: Please provide an explanation why different MOIs and infection times were used for infection of endothelial and epithelial cells. Were endothelial and epithelial cells tested for viability following infection with Nm? Live cell imaging in Figure 3D and Figure 5G were performed with preformed bacterial aggregates, this is not mentioned in the legend to Figure 5G. Please include in the Materials and Methods how infection with preformed aggregates was done.
Protocol optimizations were made to achieve a similar level of infection in both cell types, allowing to get approx. 1 bacterial colony per cell, with sizes of bacterial aggregated within the same range, while minimizing cell death. No apparent sign of cell death (excessive blebbing, nuclear fragmentation, loss of membrane dynamics and organelle movement by live-cell imaging) was observed in our experimental conditions. **This has now been clearly explained in the Methods section of the revised manuscript (lines 677-682).**

We are sorry for having omitted to mentioned that experiments in Fig. 5G were performed with preformed bacterial aggregates. **This information is now included in the revised version of the corresponding figure legends and the Methods section (lines 706-723).**

- The authors identified PDLIM and ABL1 during the AP-MS approach and could validate a local enrichment of PDLIM and ABL1 underneath bacterial microcolonies. Although they could not achieve efficient silencing of these proteins, they should briefly address the possible roles of these proteins in the discussion.
We now speculate on the possible roles of these proteins in the Discussion of the revised version of our manuscript (lines 525-539).

- I do not find a reference to Figure 9 in the manuscript text.
Reference to Fig. 9 was provided in the Discussion (ex-line 440, line 465 of the revised manuscript) in an attempt to draw a working model for our findings.

Referee #3:

This is a beautiful electron-microscopy description of how cell membranes and the underlying cytoskeleton form tubular protrusions, induced by Neisseria meningitidis. The main result of this study is that actin networks form completely differently in endothelial and epithelial cells.
I have some critics about the interpretation of some of the results, and the lack of clarity about some implicit statements.

We thank Referee #3 for the positive comments. Responses to the specific points he/she raised are provided below.

One dimensional wetting (1DW) is an interpretation that has no relevance here, as none of the possible parameters of this model are used in analysing the results. The use of "membrane protrusions formed by 1DW" is obscure without challenging the model. "Tubular membranes" should be sufficiently explicit to understand what are those protrusions.
We apologize for this lack of clarity. According to Referee #3 suggestion, **we have changed the use of "membrane protrusions formed by 1DW" for "tubular membrane protrusions" throughout the revised version of our manuscript.**

I do not understand the sense of this sentence: "our data highlight distinct mechanisms that support the formation of linear actin bundles in epithelial cell-derived protrusions, but an Arp2/3-dependent branched F-actin network".
We apologize if our wording was not sufficiently clear. Here, we intended to emphasize that tubular membrane protrusions induced by meningococci in epithelial cells lead to the assembly of linear F-actin filaments, whereas in endothelial cells it results in the polymerization of a branched F-actin network. **The sentence has been reworded to improve clarity.**

About Fig 1A-C and Fig 2A-C about the enrichment of the plasma membrane, it is stated that the enrichment is "to a lesser extent in infected epithelial cells compared to infected endothelial cells (Fig. 1A-C)". Same in Figure 2 A-C. Is the total fluorescence intensity a good measure? The apparent difference of the distributions may be due to cluster size, the total plasma membrane or actin amount being constant. By the way, this cluster size difference would support the different organisation of actin that is further observed.
We are not certain to understand fully the point raised here and we apologize in case we are not answering properly. Our image analysis pipeline was specifically developed to quantify fluorescence intensities in infected versus non infected areas of each cell, before being normalized by cell (see details in Fig. EV1) to allow averaging and relative comparisons. We are confident that this method, co-developed with the Image analysis Hub at Institut Pasteur, permits robust comparisons between conditions and cell types, regardless of the initial state of individual cells, such as amount of protein (F-actin for instance) or intensity of the fluorescent signal (GFP transient transfection) in the different cell types. In addition, the sole aim of this information was to substantiate the description of our quantifications, but do not influence any conclusions drawn in the manuscript.

To rule out a possible effect of the size of bacterial aggregates on driving the assembly of either linear F-actin filaments *versus* branched networks, we performed a correlation analysis per cell type between this parameter (colony size) and the normalized fluorescence intensity (as described in the Methods section and Fig. EV1) of F-actin or plasma membrane in each cell. No apparent correlation was found.

"Nm" should be defined
We apologize for this omission. "*Nm*" stands for "*Neisseria meningitidis*" that we have now rather abbreviated "*N. meningitidis*" for enhanced readability.

"deformation of the plasma membrane by 1DW upon N. meningitidis adhesion at the surface of epithelial or endothelial cells triggers the formation of..." I do not see how this "trigger" mechanism can be demonstrated. The authors do not show what precedes what. Actin polymerisation could very well deform the membrane, especially through the ARP2/3 branching mechanism, as has been shown in reconstituted systems (Simon, Nature Physics 2019)

"a given cell type will preferentially utilize either Arp2/3- or Formin-dependent actin nucleation machineries in response to membrane deformation" Again, the fact that membrane deformation precedes actin polymerisation is not clearly demonstrated. Is it important here to force this chronology that way?

We agree with Referee #3 that plasma membrane protrusions are generally supported by the reorganization of the actin cytoskeleton, which provides the forces required to push the membrane forward, as observed in filopodia or lamellipodia. This was the initial mechanism proposed by others to explain the formation of the plasma membrane protrusions induced by meningococcal infections (Bernard et al, 2014; Coureuil et al, 2010; Dos Santos Souza et al, 2020; Eugene et al, 2002).

However, in contrast to this initial hypothesis, our previous results have shown that bacteria-induced protrusions form in the absence of F-actin (Soyer et al., 2014; Charles-Orzsag et al., 2018). This was shown for instance by using actin polymerization inhibitors such as CytochalasinD or LatrunculinA. Furthermore, this study also shows that adhesive nanofibers can trigger the formation of similar cellular protrusions in reconstituted in vitro systems without actin. We thus have previously demonstrated this unexpected sequence of event, membrane deformation first, and then actin reorganization inside the protrusion.

The purpose of this manuscript is precisely to better understand the signaling cascade involved in the connection between plasma membrane deformation and the subsequent reorganization of the actin cytoskeleton. Importantly, we also confirm in this manuscript this sequence of event, as siRNA-mediated silencing or transfection of dominant-negative mutants of Arf1, Cdc42, N-WASP, and Arp2/3 all led to a decrease in F-actin enrichment underneath bacterial aggregates, without affecting the formation of plasma membrane tubular protrusions.

Dear Dr. Obino,

Thank you for the submission of your revised manuscript to our editorial offices. I have now received the reports from the three referees that were asked to re-evaluate the study, you will find below. As you will see, the referees now fully support the publication of the study in EMBO reports.

Before we can proceed with formal acceptance, I have the editorial requests below I ask you to address in a final revised manuscript. Please also provide a final p-b-p-response to the editorial requests.

Editorial requests:

- Please order the manuscript sections like this, using only these names:
Title page - Abstract - Keywords - Introduction - Results - Discussion - Methods - Data availability section - Acknowledgements - Disclosure and Competing Interests Statement - References - Figure legends - Expanded View Figure legends

Please remove the running title and merge the funding information with the Acknowledgements.

- We now use CRediT to specify the contributions of each author in the journal submission system. CRediT replaces the author contribution section. Please use the free text box to provide more detailed descriptions and do NOT provide your final manuscript text file with an author contributions section. See also our guide to authors (section 'Author contributions'): https://link.springer.com/journal/44319/submission-guidelines#cms-Revised-submissions

- Please remove the ORCID ID's from the title page of the manuscript. The IDs are already linked to the corresponding authors in our system and will be displayed directly in the online version of the paper.

- Please provide a current email address for co-author Léa Swistak. Upon re-submission, we have received an automatic reply indicating the address provided is not anymore valid ("The person you are trying to contact is no longer staff member of Institut Pasteur, this email address is no longer active.").

- Please combine some of the EV figures in order to have not more than 6 final EV figures. Please update the legends and the callouts accordingly.

- Please list all corresponding authors in the author checklist.

- Please check again that the number "n" for how many independent experiments were performed, their nature (biological versus technical replicates), the bars and error bars (e.g. SEM, SD) and the test used to calculate p-values is indicated in the respective figure legends (main and EV figures). Please also check that all the p-values are explained in the legend, and that these fit to those shown in the figure. Please provide statistical testing where applicable. Please avoid the phrase 'independent experiment' but clearly state if these were biological or technical replicates. Please also indicate (e.g. with n.s.) if testing was performed, but the differences are not significant. In case n=2, please show the data as separate datapoints without error bars and statistics. See also:

https://link.springer.com/journal/44319/submission-guidelines#cms-Figure-and-data-presentation

If n<5, please show single datapoints for diagrams. Presently, most diagrams lack statistics or the 'n.s.'. Please provide proper and complete statistical testing for all diagrams shown. Moreover:
- Please note that the exact p values are not provided in the legends of figures 1C, G; 2C, 3C, G; 4C, F, I; 5C, F; 6C, F, I; 7G, 8C, F, I; EV2 C, EV4 E, EV7 C, F; EV9 C, F.
- Please indicate the statistical test used for data analysis in the legends of figures EV6 B, C
- Please note that the yellow arrows are not defined in the legend of figure 1D. This needs to be rectified.

- Please remove now the referee access from the data availability section and make sure the dataset is public latest upon online publication of the paper. Please add here also any information (accession number, full link) for source data files deposited at external repositories.

- Please make sure that all the funding information is also entered into the online submission system and that it is complete and similar to the one in the acknowledgement section of the manuscript text file. Presently, these grants are missing from the submission system: École Doctorale Bio Sorbonne Paris Cité (BioSPC), Université Paris Cité and by Institut Pasteur "Programme Federateur de Recherche" (PFR-6-SI-COV2 project), Urgence COVID-19 Fundraising Campaign of Institut Pasteur; French National Research Agency (ANR-10-INBS-04; Investments for the Future), the Région Ile-de-France (program DIM1HEALTH), and the French Government Investissement d'Avenir Programme-Laboratoire d'Excellence 'Integrative Biology of Emerging Infectious Diseases' (ANR-10-LABX-62-IBEID); grant from the French Government's 'Investissements d'Avenir'

program (EQUIPEX CACSICE - "Centre d'analyse de systèmes complexes dans les environnements complexes", ANR-11-EQPX-0008); grant from Région Ile-de-France (DIM ELICIT2019).

- Please remove the instructions text and the example part from the reagents and tools table.

- Tables EV1-EV3 are datasets. Please upload these as Datasets with the nomenclature 'Dataset EVx'. Please provide their legends on the first TAB of the respective excel file. Moreover, please update all callouts in manuscript and remove the legend for EV tables from the manuscript text file.

- Presently, Movies EV5 and EV6 cannot be opened. Please check and make sure that they play. Moreover, please provide each movie legend as separate text file (one per movie), ZIPed together with the respective movie file and uploaded (as one item). Finally, please remove the movie legends from the manuscript text file.

In addition, I would need from you uploaded separately:
- a short, two-sentence summary of the manuscript (not more than 35 words).
- two to four short (!) bullet points highlighting the key findings of your study (two lines each).
- a schematic summary figure as separate file that provides a sketch of the major findings (not a data image) in jpeg or tiff format (with the exact width of 550 pixels and the exact height of 300 pixels) that can be used as a visual synopsis on our website.

I look forward to seeing the further revised version of your manuscript when it is ready. Please let me know if you have questions regarding the revision.

Best,

Achim Breiling
Senior Editor
EMBO Reports

Referee #1:

I want to thank the authors for their careful revision and for taking the time to address my comments in a thoughtful and rigorous manner. I believe the additional experiments and clarifications have significantly strengthened the manuscript.
In particular, the new data supporting T4P-dependent Arf1 recruitment and the ordering of the Arf1-Cdc42 pathway add important mechanistic depth. The inclusion of proximity ligation assay in Fig. EV7G is also a valuable addition, providing spatial support for the proposed interaction.

The manuscript overall is very well written and designed as I previously mentioned and now presents a more convincing framework linking bacterial adhesion to cytoskeletal remodeling. I have no further major concerns and support publication in its current form.

Referee #2:

In their revised version of the manuscript the authors have adequately dealt with all points of concern. I have no further points of criticism.

Referee #3:

This manuscript has been satisfactorily corrected, and all reviewers' comments have been addressed correctly in the last version.

Point-by-point response to the editorial requests

We thank you and the Referees for the positive comments that support the publication of the revised version of our manuscript in EMBO Reports. Provided below in blue is our point-by-point response to the editorial requests.

- Please order the manuscript sections like this, using only these names: Title page - Abstract - Keywords - Introduction - Results - Discussion - Methods - Data availability section - Acknowledgements - Disclosure and Competing Interests Statement - References - Figure legends - Expanded View Figure legends.

The sections in the final version of the manuscript have been reordered according to the above request.

Please remove the running title and merge the funding information with the Acknowledgements.

The running title has been removed and the funding information fused with the acknowledgements section.

- We now use CRediT to specify the contributions of each author in the journal submission system. CRediT replaces the author contribution section. Please use the free text box to provide more detailed descriptions and do NOT provide your final manuscript text file with an author contributions section. See also our guide to authors (section 'Author contributions'):https://link.springer.com/journal/44319/submission-guidelines#cms-Revised-submissions

The author contributions section has been removed from the final version of the manuscript file and contributions of each author have been specified in the journal submission system.

- Please remove the ORCID ID's from the title page of the manuscript. The IDs are already linked to the corresponding authors in our system and will be displayed directly in the online version of the paper.

The ORCID ID’s have been removed from the title page.

- Please provide a current email address for co-author Léa Swistak. Upon re-submission, we have received an automatic reply indicating the address provided is not anymore valid ("The person you are trying to contact is no longer staff member of Institut Pasteur, this email address is no longer active.").

We apologize for having provided the wrong email address for Léa Swistak. This has now been modified for a valid email address: lea.swistak@embl.de.

- Please combine some of the EV figures in order to have not more than 6 final EV figures. Please update the legends and the callouts accordingly.

EV Figures 1 and 2 have not been modified.
Former EV Figures 3, 4 and 5 have been fused in the new EV Figure 3.
Former EV Figure 6 is now EV Figure 4.
Former EV Figure 7 is now EV Figure 5.
Former EV Figures 8 and 9 have been fused in the new EV Figure 6.
All legends and callouts have been updated in the final version of the manuscript file.

- Please list all corresponding authors in the author checklist.

The two corresponding authors, Dorian Obino and Guillaume Duménil, have now been listed in the author checklist.

- Please check again that the number "n" for how many independent experiments were performed, their nature (biological versus technical replicates), the bars and error bars (e.g. SEM, SD) and the test used to calculate p-values is indicated in the respective figure legends (main and EV figures). Please also check that all the p-values are explained in the legend, and that these fit to those shown in the figure. Please provide statistical testing where applicable. Please avoid the phrase 'independent experiment' but clearly state if these were biological or technical replicates. Please also indicate (e.g. with n.s.) if testing was performed, but the differences are not significant. In case n=2, please show the data as separate datapoints without error bars and statistics.
If n<5, please show single datapoints for diagrams. Presently, most diagrams lack statistics or the 'n.s.'. Please provide proper and complete statistical testing for all diagrams shown.

All these points have been double-checked and are respected in the final version of the manuscript. Statistical differences were indicated by providing the exact value of the p-values (rather than n.s., *, **, ***, ****), considering differences are statistically significant when p-values were inferior at 0.05.

Moreover:

- Please note that the exact p values are not provided in the legends of figures 1C, G; 2C, 3C, G; 4C, F, I; 5C, F; 6C, F, I; 7G, 8C, F, I; EV2 C, EV4 E, EV7 C, F; EV9 C, F.

GraphPad Prism software (v.10 and v.11) was used to assess statistical significance. Exact p-values are provided until the 0.0001 threshold. P-values below 0.0001 are provided as “p<0.0001” and were displayed as such in the figure panels.

- Please indicate the statistical test used for data analysis in the legends of figures EV6 B, C

The statistical test used to analyze the data shown in former EV Figure 6B, C (now EV Figure 4B, C) was provided in the Method section and has now been added to the corresponding figure legend : *“Statistical testing was conducted using the limma t-test*

thanks to the R package limma and adaptive Benjamini-Hochberg procedure was applied on the resulting p-values thanks to the function adjust.p of the cp4p R package."

- Please note that the yellow arrows are not defined in the legend of figure 1D. This needs to be rectified.

The yellow arrows have been defined in the legend of figure 1D.

- Please remove now the referee access from the data availability section and make sure the dataset is public latest upon online publication of the paper. Please add here also any information (accession number, full link) for source data files deposited at external repositories.

The dataset deposited on the ProteomeXchange Consortium via the PRIDE partner repository is now publicly available and the referee access information have been removed from the data availability section. The dataset is available at https://www.ebi.ac.uk/pride/archive/projects/PXD045648 under the dataset identifier PXD045648. No other source data files have been deposited elsewhere.

- Please make sure that all the funding information is also entered into the online submission system and that it is complete and similar to the one in the acknowledgement section of the manuscript text file. Presently, these grants are missing from the submission system: École Doctorale Bio Sorbonne Paris Cité (BioSPC), Université Paris Cité and by Institut Pasteur "Programme Federateur de Recherche" (PFR-6-SI-COV2 project), Urgence COVID-19 Fundraising Campaign of Institut Pasteur; French National Research Agency (ANR-10-INBS-04; Investments for the Future), the Région Ile-de-France (program DIM1HEALTH), and the French Government Investissement d'Avenir Programme-Laboratoire d'Excellence 'Integrative Biology of Emerging Infectious Diseases' (ANR-10-LABX-62-IBEID); grant from the French Government's 'Investissements d'Avenir' program (EQUIPEX CACSICE - "Centre d'analyse de systèmes complexes dans les environnements complexes", ANR-11-EQPX-0008); grant from Région Ile-de-France (DIM ELICIT2019).

All funding information and missing grants have been entered into the online submission system.

- Please remove the instructions text and the example part from the reagents and tools table.

The instructions text and the example part from the reagents and tools table have been removed.

- Tables EV1-EV3 are datasets. Please upload these as Datasets with the nomenclature 'Dataset EVx'. Please provide their legends on the first TAB of the

respective excel file. Moreover, please update all callouts in manuscript and remove the legend for EV tables from the manuscript text file.

Fomer Tables EV1-EV3 have been modified according to the request, their legend included in the first tab of each file (and removed from the manuscript file) and callouts throughout the manuscript file updated accordingly.

- Presently, Movies EV5 and EV6 cannot be opened. Please check and make sure that they play. Moreover, please provide each movie legend as separate text file (one per movie), ZIPed together with the respective movie file and uploaded (as one item). Finally, please remove the movie legends from the manuscript text file.

We are surprised that you could not open Movies EV5 and EV6, as they played properly on different computers on our side. We have nevertheless converted both movies in a MP4 format, hoping you will be able to play them (of note, both converted movies played properly using the VLC free player).
Movie legends are now provided alongside each movie within an individual zipped item and have been removed from the final version of the manuscript.

In addition, I would need from you uploaded separately:

- a short, two-sentence summary of the manuscript (not more than 35 words).

A short, two-sentence summary of the study is now provided alongside the final version of the manuscript.

- two to four short (!) bullet points highlighting the key findings of your study (two lines each).

Bullet points highlighting the key findings of our study are now provided alongside the final version of the manuscript.

- a schematic summary figure as separate file that provides a sketch of the major findings (not a data image) in jpeg or tiff format (with the exact width of 550 pixels and the exact height of 300 pixels) that can be used as a visual synopsis on our website.

A schematic summary figure is now provided as jpeg format and replaces former Figure 9.

Dr. Dorian Obino
Institut Pasteur
INSERM U1225
Paris 75015
France

Dear Dr. Obino,

I am very pleased to accept your manuscript for publication in the next available issue of EMBO reports. Thank you for your contribution to our journal.

Your manuscript will be processed for publication by EMBO Press. It will be copy edited and you will receive page proofs prior to publication. Please note that you will be contacted by Springer Nature Author Services to complete licensing and payment information.

You may qualify for financial assistance for your publication charges - either via a Springer Nature fully open access agreement or an EMBO initiative. Check your eligibility: https://link.springer.com/journal/44319/how-to-publish-with-us

Should you be planning a Press Release on your article, please notify the production team via this form in order to coordinate publication and release dates: https://forms.cloud.microsoft/e/eH4A8JVP6g

If you have any questions, please do not hesitate to contact the Editorial Office. Thank you for your contribution to EMBO Reports.

Yours sincerely,

Achim Breiling
Senior Editor
EMBO Reports

>>> Please note that it is EMBO Reports policy for the transcript of the editorial process (containing referee reports and your response letter) to be published as an online supplement to each paper. If you do NOT want this, you will need to inform the Editorial Office via email immediately. More information is available here: https://link.springer.com/partners/embo-press/editorial-policies#Peer%20review

EMBO Press Author Checklist

Corresponding Author Name: OBINO Dorian & DUMENIL Guillaume	
Journal Submitted to: EMBO Reports	
Manuscript Number: EMBOR-2025-62972-T	

USEFUL LINKS FOR COMPLETING THIS FORM
The EMBO Journal - Author Guidelines
EMBO Reports - Author Guidelines
Molecular Systems Biology - Author Guidelines
EMBO Molecular Medicine - Author Guidelines

Reporting Checklist for Life Science Articles (updated January
This checklist is adapted from Materials Design Analysis Reporting (MDAR) Checklist for Authors. MDAR establishes a minimum set of requirements in transparent reporting in the life sciences (see Statement of Task: 10.31222/osf.io/9sm4x). Please follow the journal's guidelines in preparing your
Please note that a copy of this checklist will be published alongside your article.

Abridged guidelines for figures
1. Data
The data shown in figures should satisfy the following conditions:
- the data were obtained and processed according to the field's best practice and are presented to reflect the results of the experiments in an accurate and unbiased manner.
- ideally, figure panels should include only measurements that are directly comparable to each other and obtained with the same assay.
- plots include clearly labeled error bars for independent experiments and sample sizes. Unless justified, error bars should not be shown for technical
- if n<5, the individual data points from each experiment should be plotted. Any statistical test employed should be justified.
- Source Data should be included to report the data underlying figures according to the guidelines set out in the authorship guidelines on Data

2. Captions
Each figure caption should contain the following information, for each panel where they are relevant:
- a specification of the experimental system investigated (eg cell line, species name).
- the assay(s) and method(s) used to carry out the reported observations and measurements.
- an explicit mention of the biological and chemical entity(ies) that are being measured.
- an explicit mention of the biological and chemical entity(ies) that are altered/varied/perturbed in a controlled manner.
- the exact sample size (n) for each experimental group/condition, given as a number, not a range;
- a description of the sample collection allowing the reader to understand whether the samples represent technical or biological replicates (including how many animals, litters, cultures, etc.).
- a statement of how many times the experiment shown was independently replicated in the laboratory.
- definitions of statistical methods and measures:
  - common tests, such as t-test (please specify whether paired vs. unpaired), simple χ2 tests, Wilcoxon and Mann-Whitney tests, can be unambiguously identified by name only, but more complex techniques should be described in the methods section;
  - are tests one-sided or two-sided?
  - are there adjustments for multiple comparisons?
  - exact statistical test results, e.g., P values = x but not P values < x;
  - definition of 'center values' as median or average;
  - definition of error bars as s.d. or s.e.m.

Please complete ALL of the questions below.
Select "Not Applicable" only when the requested information is not relevant for your study.

Materials

Newly Created Materials	Information included in the manuscript?	In which section is the information available? (Reagents and Tools Table, Materials and Methods, Figures, Data Availability Section)
New materials and reagents need to be available; do any restrictions apply?	Yes	Materials and Methods + Reagents and Tools Table

Antibodies	Information included in the manuscript?	In which section is the information available? (Reagents and Tools Table, Materials and Methods, Figures, Data Availability Section)
For **antibodies** provide the following information:<br>- Commercial antibodies: RRID (if possible) or supplier name, catalogue number and or/clone number<br>- Non-commercial: RRID or citation	Yes	Materials and Methodes section "Antibodies" + Reagents and Tools Table section '"Antibodies"

DNA and RNA sequences	Information included in the manuscript?	In which section is the information available? (Reagents and Tools Table, Materials and Methods, Figures, Data Availability Section)
**Short novel DNA or RNA including primers, probes**: provide the sequences.	Yes	Materials and Methodes section "siRNA-mediated protein silencing" + Reagents and Tools Table "Oligonucleotides and other sequence-based reagents"

Cell materials	Information included in the manuscript?	In which section is the information available? (Reagents and Tools Table, Materials and Methods, Figures, Data Availability Section)
**Cell lines:** Provide species information, strain. Provide accession number in repository **OR** supplier name, catalog number, clone number, and/**OR** RRID.	Yes	Materials and Methodes section "Cell Culture" + Reagents and Tools Table section "Experimental Models"
**Primary cultures:** Provide species, strain, sex of origin, genetic modification status.	Yes	Materials and Methodes section "Cell Culture" + Reagents and Tools Table section "Experimental Models"
Report if the cell lines were recently **authenticated** (e.g., by STR profiling) and tested for mycoplasma contamination.	Not Applicable	/

Experimental animals	Information included in the manuscript?	In which section is the information available? (Reagents and Tools Table, Materials and Methods, Figures, Data Availability Section)
**Laboratory animals or Model organisms:** Provide species, strain, sex, age, genetic modification status. Provide accession number in repository **OR** supplier name, catalog number, clone number, **OR** RRID.	Not Applicable	/
**Animal observed in or captured from the field:** Provide species, sex, and age where possible.	Not Applicable	/
Please detail **housing and husbandry conditions**.	Not Applicable	/

Plants and microbes	Information included in the manuscript?	In which section is the information available? (Reagents and Tools Table, Materials and Methods, Figures, Data Availability Section)
**Plants:** provide species and strain, ecotype and cultivar where relevant, unique accession number if available, and source (including location for collected wild specimens).	Not Applicable	/
**Microbes:** provide species and strain, unique accession number if available, and source.	Yes	Materiel and Methods section "Bacterial Strains and Growth Conditions" + Reagents and Tools Table section "Experimental Models"

Human research participants	Information included in the manuscript?	In which section is the information available? (Reagents and Tools Table, Materials and Methods, Figures, Data Availability Section)
If collected and within the bounds of privacy constraints report on age, sex and gender or ethnicity for all study participants.	Not Applicable	/

Core facilities	Information included in the manuscript?	In which section is the information available? (Reagents and Tools Table, Materials and Methods, Figures, Data Availability Section)
If your work benefited from core facilities, was their service mentioned in the acknowledgments section?	Yes	Authors + Acknowledgments

Design

Study protocol	Information included in the manuscript?	In which section is the information available? (Reagents and Tools Table, Materials and Methods, Figures, Data Availability Section)
If study protocol has been **pre-registered, provide DOI in the manuscript**. For clinical trials, provide the trial registration number **OR** cite DOI.	Not Applicable	
Report the **clinical trial registration number** (at ClinicalTrials.gov or equivalent), where applicable.	Not Applicable	/

Laboratory protocol	Information included in the manuscript?	In which section is the information available? (Reagents and Tools Table, Materials and Methods, Figures, Data Availability Section)
Provide DOI OR other citation details if **external detailed step-by-step protocols** are available.	Not Applicable	

Experimental study design and statistics	Information included in the manuscript?	In which section is the information available? (Reagents and Tools Table, Materials and Methods, Figures, Data Availability Section)
Include a statement about **sample size** estimate even if no statistical methods were used.	Yes	Materiel and Methods section "statistiacl analysis"
Were any steps taken to minimize the effects of subjective bias when allocating animals/samples to treatment (e.g. **randomization procedure**)? If yes, have they been described?	Not Applicable	/
Include a statement about **blinding** even if no blinding was done.	Not Applicable	
Describe **inclusion/exclusion criteria** if samples or animals were excluded from the analysis. Were the criteria pre-established?<br><br>If sample or data points were omitted from analysis, report if this was due to attrition or intentional exclusion and provide justification.	Yes	Materiel and Methods section "Analysis and quantification of fluorescence images"
For every figure, are **statistical tests** justified as appropriate? Do the data meet the assumptions of the tests (e.g., normal distribution)? Describe any methods used to assess it. Is there an estimate of variation within each group of data? Is the variance similar between the groups that are being statistically compared?	Yes	Legends of Figures + Legends of Expanded View Figures

Sample definition and in-laboratory replication	Information included in the manuscript?	In which section is the information available? (Reagents and Tools Table, Materials and Methods, Figures, Data Availability Section)
In the figure legends: state number of times the experiment was **replicated** in laboratory.	Yes	Legends of Figures + Legends of Expanded View Figures
In the figure legends: define whether data describe **technical or biological replicates**.	Yes	Legends of Figures + Legends of Expanded View Figures

Ethics

Ethics	Information included in the manuscript?	In which section is the information available? (Reagents and Tools Table, Materials and Methods, Figures, Data Availability Section)
Studies involving **human participants**: State details of **authority granting ethics approval** (IRB or equivalent committee(s), provide reference number for approval.	Not Applicable	/
Studies involving **human participants**: Include a statement confirming that **informed consent** was obtained from all subjects and that the experiments conformed to the principles set out in the WMA Declaration of Helsinki and the Department of Health and Human Services Belmont Report.	Not Applicable	/
Studies involving **human participants:** For publication of **patient photos**, include a statement confirming that consent to publish was obtained.	Not Applicable	/
Studies involving experimental **animals**: State details of **authority granting ethics approval** (IRB or equivalent committee(s), provide reference number for approval. Include a statement of compliance with ethical regulations.	Not Applicable	/
Studies involving **specimen and field samples:** State if relevant **permits** obtained, provide details of authority approving study; if none were required, explain why.	Not Applicable	/

Dual Use Research of Concern (DURC)	Information included in the manuscript?	In which section is the information available? (Reagents and Tools Table, Materials and Methods, Figures, Data Availability Section)
Could your study fall under dual use research restrictions? Please check biosecurity documents and list of **select agents and toxins** (CDC): https://www.selectagents.gov/sat/list.htm	Not Applicable	/
If you used a select agent, is the security level of the lab appropriate and reported in the manuscript?	Not Applicable	/
If a study is subject to dual use research of concern regulations, is the name of the **authority granting approval and reference number** for the regulatory approval provided in the manuscript?	Not Applicable	/

Reporting

The MDAR framework recommends adoption of discipline-specific guidelines, established and endorsed through community initiatives. Journals have their own policy about requiring specific guidelines and recommendations to complement MDAR.

Adherence to community standards	Information included in the manuscript?	In which section is the information available? (Reagents and Tools Table, Materials and Methods, Figures, Data Availability Section)
State if relevant guidelines or checklists (e.g., **ICMJE, MIBBI, ARRIVE, PRISMA**) have been followed or provided.	Not Applicable	/
For **tumor marker prognostic studies,** we recommend that you follow the **REMARK** reporting guidelines (see link list at top right). See author guidelines, under 'Reporting Guidelines'. Please confirm you have followed these guidelines.	Not Applicable	/
For **phase II and III randomized controlled trials**, please refer to the **CONSORT** flow diagram (see link list at top right) and submit the CONSORT checklist (see link list at top right) with your submission. See author guidelines, under 'Reporting Guidelines'. Please confirm you have submitted this list.	Not Applicable	/

Data Availability

Data availability	Information included in the manuscript?	In which section is the information available? (Reagents and Tools Table, Materials and Methods, Figures, Data Availability Section)
Have **primary datasets** been deposited according to the journal's guidelines (see 'Data Deposition' section) and the respective accession numbers provided in the Data Availability Section?	Yes	Data Availability Statement
Were **human clinical and genomic datasets** deposited in a public access-controlled repository in accordance to ethical obligations to the patients and to the applicable consent agreement?	Not Applicable	/
Are **computational models** that are central and integral to a study available without restrictions in a machine-readable form? Were the relevant accession numbers or links provided?	Not Applicable	/
If publicly available data were reused, provide the respective **data citations in the reference list**.	Not Applicable	/
