# Supplementary material for: Arf1 is involved in Neisseria meningitidis-induced cortical branched F-actin network reorganization
Source: EMBO Rep. 2026 Jun 25;27(15):4544–82. doi: 10.1038/s44319-026-00843-z (PMC13458072; doi:10.1038/s44319-026-00843-z)
Supplement: Supplementary file 3 [file 44319_2026_843_MOESM10_ESM.zip › Movie_EV6_Legend.docx]

**Movie EV6. RFP-wGBD is recruited in *N. meningitidis*-induced endothelial tubular protrusions together with Cdc42-WT-GFP.** Live imaging of human hTERT-immortalized endothelial cells transiently co-expressing the active GTP-bound Cdc42 reporter RFP-wGBD and Cdc42-WT-GFP and infected with preformed aggregates of iRFP-expressing *N. meningitidis*. Scale bar: 5 µm. Time is shown as mm:ss.
