# Supplementary material for: Arf1 is involved in Neisseria meningitidis-induced cortical branched F-actin network reorganization
Source: EMBO Rep. 2026 Jun 25;27(15):4544–82. doi: 10.1038/s44319-026-00843-z (PMC13458072; doi:10.1038/s44319-026-00843-z)
Supplement: Supplementary file 4 [file 44319_2026_843_MOESM11_ESM.zip › EMBOR-2025-62972V2_SourceData-Figure1/Fig.1_Panel_A/README.rtf]

Figure 1A - High-resolution microscopic images (maximum intensity z-projection):
Endothelial cell:
Channel1: PM-eGFP (plasma membrane)
Channel2: CD31 detected with a specific antibody (plasma membrane, not displayed on the figure panel)
Channel3: Phalloidine (F-Actin, not displayed on the figure panel) 
Channel4: iRFFP_expressing Neisseria meningitidis

Epithelial cell:
Channel1: DAPI (not displayed on the figure panel)
Channel2: CD326/EpCAM detected with a specific antibody (plasma membrane)
Channel3: Phalloidine (F-Actin, not displayed on the figure panel) 
Channel4: iRFFP_expressing Neisseria meningitidis
