# Supplementary material for: Arf1 is involved in Neisseria meningitidis-induced cortical branched F-actin network reorganization
Source: EMBO Rep. 2026 Jun 25;27(15):4544–82. doi: 10.1038/s44319-026-00843-z (PMC13458072; doi:10.1038/s44319-026-00843-z)
Supplement: Supplementary file 6 [file 44319_2026_843_MOESM13_ESM.zip › EMBOR-2025-62972V2_SourceData-Figure3/Fig.3_Panel_A/README.rtf]

Figure 3A - High-resolution microscopic images (maximum intensity z-projection):
Channel1: PM-eGFP (plasma membrane)
Channel2: Arp2 detected with a specific antibody
Channel3: Phalloidine (F-Actin) 
Channel4: iRFFP_expressing Neisseria meningitidis
