# Supplementary material for: Arf1 is involved in Neisseria meningitidis-induced cortical branched F-actin network reorganization
Source: EMBO Rep. 2026 Jun 25;27(15):4544–82. doi: 10.1038/s44319-026-00843-z (PMC13458072; doi:10.1038/s44319-026-00843-z)
Supplement: Supplementary file 7 [file 44319_2026_843_MOESM14_ESM.zip › EMBOR-2025-62972V2_SourceData-Figure4/Fig.4_Panel_D/README.rtf]

Figure 4D - High-resolution microscopic images (maximum intensity z-projection):
Channel1: DAPI (not shown)
Channel2: PM-eGFP (Plasma membrane)
Channel3: Phalloidine (F-Actin)
Channel4: iRFP-expressing Neisseria meningitidis
