# Supplementary material for: Arf1 is involved in Neisseria meningitidis-induced cortical branched F-actin network reorganization
Source: EMBO Rep. 2026 Jun 25;27(15):4544–82. doi: 10.1038/s44319-026-00843-z (PMC13458072; doi:10.1038/s44319-026-00843-z)
Supplement: Supplementary file 8 [file 44319_2026_843_MOESM15_ESM.zip › EMBOR-2025-62972V2_SourceData-Figure5/Fig.5_Panel_A/README.rtf]

Figure 5A - High-resolution microscopic images (maximum intensity z-projection):
Channel1: GFP signal (Rac1-WT or Rac1-DN)
Channel2: Phalloidine (F-Actin)
Channel3: iRFP-expressing Neisseria meningitidis
Channel4: DAPI (not shown)
