# Supplementary material for: Arf1 is involved in Neisseria meningitidis-induced cortical branched F-actin network reorganization
Source: EMBO Rep. 2026 Jun 25;27(15):4544–82. doi: 10.1038/s44319-026-00843-z (PMC13458072; doi:10.1038/s44319-026-00843-z)
Supplement: Supplementary file 10 [file 44319_2026_843_MOESM17_ESM.zip › EMBOR-2025-62972V2_SourceData-Figure7/Fig.7_Panel_D/README.rtf]

Figure 7D - High-resolution microscopic images (average intensity z-projection):
Channel1: Arf1-GFP 
Channel2: GM130 detected by specific antibody (Golgi apparatus)
Channel3: iRFP-expressing Neisseria meningitidis
