# Supplementary material for: Arf1 is involved in Neisseria meningitidis-induced cortical branched F-actin network reorganization
Source: EMBO Rep. 2026 Jun 25;27(15):4544–82. doi: 10.1038/s44319-026-00843-z (PMC13458072; doi:10.1038/s44319-026-00843-z)
Supplement: Supplementary file 11 [file 44319_2026_843_MOESM18_ESM.zip › EMBOR-2025-62972V2_SourceData-Figure8/Fig.8_Panel_D/README.rtf]

Figure 8D - High-resolution microscopic images (maximum intensity z-projection):
Channel1: PM-eGFP (plasma membrane)
Channel2: Phalloidine (F-Actin)
Channel3: iRFP-expressing Neisseria meningitidis
