# Supplementary material for: Arf1 is involved in Neisseria meningitidis-induced cortical branched F-actin network reorganization
Source: EMBO Rep. 2026 Jun 25;27(15):4544–82. doi: 10.1038/s44319-026-00843-z (PMC13458072; doi:10.1038/s44319-026-00843-z)
Supplement: Supplementary file 13 [file 44319_2026_843_MOESM6_ESM.zip › Movie_EV2_Legend.docx]

**Movie EV2. FIB-SEM imaging of endothelial-derived membrane tubular protrusions formed upon *N. meningitidis* adhesion at the cell surface.** FIB-SEM volume and 3D segmentation of a human primary endothelial cell infected with meningococci and forming membrane tubular protrusions. Scale bar: 1000 nm.
