# Supplementary material for: Arf1 is involved in Neisseria meningitidis-induced cortical branched F-actin network reorganization
Source: EMBO Rep. 2026 Jun 25;27(15):4544–82. doi: 10.1038/s44319-026-00843-z (PMC13458072; doi:10.1038/s44319-026-00843-z)
Supplement: Supplementary file 14 [file 44319_2026_843_MOESM7_ESM.zip › Movie_EV3_Legend.docx]

**Movie EV3. Ultrastructural organization of F-actin networks in epithelial-derived membrane tubular protrusions formed upon *N. meningitidis* adhesion at the cell surface.** Cryo-tomogram and 3D segmentation of actin filaments within *N. meningitidis*-induced membrane tubular protrusions in human primary epithelial cells. Scale bar: 500 nm
